# Benchmarking tools for deciphering cellular crosstalk in spatially-resolved transcriptomics

**DOI:** 10.1186/s13059-026-04063-5

**Published:** 2026-04-08

**Authors:** Li-Ting Ku, Vincent Bernard, Jimin Min, Ying Yuan, Eugene Jon Koay, Anirban Maitra, Liang Li, Ziyi Li

**Affiliations:** 1 Department of Biostatistics, The University of Texas MD Anderson Cancer Center, Houston, TX USA; 2 Graduate School of Biomedical Sciences, The University of Texas MD Anderson Cancer Center, Houston, TX USA; 3 Department of Gastrointestinal Radiation Oncology, The University of Texas MD Anderson Cancer Center, Houston, TX USA; 4 Perlmutter Cancer Center, Department of Medicine, New York University Grossman School of Medicine, NYU Langone Health, New York, NY USA; 5 Department of Pathology, New York University Grossman School of Medicine, NYU Langone Health, New York, NY USA

## Abstract

**Background:**

Cell-cell communication via ligand–receptor signaling is a fundamental mechanism shaping multicellular organization and functional heterogeneity within tissue microenvironments. Recent advances in spatial transcriptomics (ST) have enabled unprecedented opportunities to systematically infer such interactions under the native spatial context. While prior studies have summarized or compared existing cell–cell interaction (CCI) inference methods, comprehensive benchmarking of tools specifically developed for ST applications remains limited.

**Results:**

Here, we present a comprehensive evaluation of nine computational CCI inference methods on a series of realistic simulation settings and nine real ST datasets from three independent studies, spanning Visium, Stereo-seq, and Xenium platforms. Method performance was assessed based on ligand-receptor prediction accuracy, spatial coherence of interactions, biological relevance via pathway enrichment, and computational efficiency.

**Conclusions:**

Our results demonstrate substantial variability in tool performance across spatial resolutions, tissue contexts, and platforms, offering practical guidance for tool selection. This study also highlights key challenges in applying existing tools to real ST data and provides insights to inform future advances in spatially resolved cell–cell interaction analysis.

**Supplementary Information:**

The online version contains supplementary material available at 10.1186/s13059-026-04063-5.

## Background

Multicellular organisms maintain complex tissue and organ systems through interactions among diverse cell types [1, 2], which coordinates biological processes such as development [3], homeostasis [4, 5], and immune responses [6, 7]. Although cells utilize multiple communication modalities including direct physical contact, soluble signaling molecules, and extracellular vesicles, a substantial proportion of cellular crosstalk is mediated through ligand-receptor (L-R) interactions [8–10]. This form of cell-cell interaction (CCI) involves the exchange of context-specific biochemical signals between sender and receiver cells, guiding gene expression and functional behavior [11, 12].

The advent of spatial transcriptomics (ST) technology [13], which maps the gene expression onto spatial tissue architecture, enables researchers to investigate how CCIs are spatially organized. Various methods have been developed to infer these interactions by integrating both molecular profiles with spatial proximity. For example, CellChat v2 [14] applies a probabilistic framework incorporating spatial context; CellPhoneDB v3 [3, 15, 16] identifies interacting cell-type pairs using mean expression levels within user-defined microenvironments. COMMOT [17] and SCOTIA [18] leverage optimal transport theory to model signaling fluxes, while SpaTalk [19] constructs graph-based neighborhoods and SpatialDM [20] use spatial autocorrelation to detect ligand–receptor co-expression. NicheDE [21] infers cell-cell communication through differential expression analysis between sender and receiver cell-type niches. A recent approach, CellNEST [22], models localized cellular interactions at a single-cell resolution using graph neural networks and contrastive learning on spatial neighborhood graphs. We recently developed SpaCCI [23], which employs non-negative least squares regression and probabilistic modeling to quantify directed, cell-type-specific interactions in spatially mixed spots. Together, these approaches highlight the growing methodological diversity and innovation in spatially resolved CCI inference.

Despite the increasing emergence of spatial CCI inference methods, no systematic study has comprehensively evaluated computational CCI inference methods across diverse spatial transcriptomics platforms. Existing benchmarking efforts have largely focused on non-spatial single-cell RNA-seq data. For example, Wang et al. and Xie et al. performed systematic and comparative evaluations of L-R–based CCI methods using single-cell RNA-seq datasets, assessing method performance in terms of precision, sensitivity, robustness, and dependence on L-R databases, but without incorporating spatial information [24, 25]. Other studies have primarily taken a review-oriented perspective. Dimitrov et al. compared different L-R resources and evaluated the robustness of early computational frameworks for CCI inference in single-cell RNA-seq data [26], while more recent work by Cesaro et al. provided a comprehensive review summarizing methodological advances, databases, and open challenges in the field without performing quantitative benchmarking [27].

More recent work has begun to incorporate spatial context, but typically with a different focus or limited scope. Zeng et al. reviewed statistical and machine learning methods for spatially resolved transcriptomics data analysis more broadly, with an emphasis on spatial gene expression patterns rather than benchmarking spatial CCI inference tools [28]. Liu et al. evaluated CCI methods by integrating single-cell RNA-seq data with spatial information, but focused on a limited number of methods spanning both non-spatial and spatial settings and did not perform a systematic benchmark of CCI inference methods specifically designed for spatial transcriptomics platforms [29]. As a result, a systematic and quantitative benchmarking of spatial CCI inference methods across multiple ST platforms remains lacking.

Here, we present a comprehensive evaluation of nine recent computational-based spatial CCI approaches using both simulated and real ST datasets, spanning single-cell and spot-level resolutions. We assess performance in detecting simulation-defined truth interactions and recovering biologically supported signaling pathways. Our analysis highlights trade-offs in sensitivity, spatial resolution, and computational scalability, providing practical guidance for method selection. To promote transparency and broader adoption, we provide an open-access resource with annotated datasets and reproducible benchmarking code (https://benchmarking-cci.readthedocs.io/en/latest/index.html).

## Results

### Diverse strategies to quantify CCIs

**Fig. 1:**
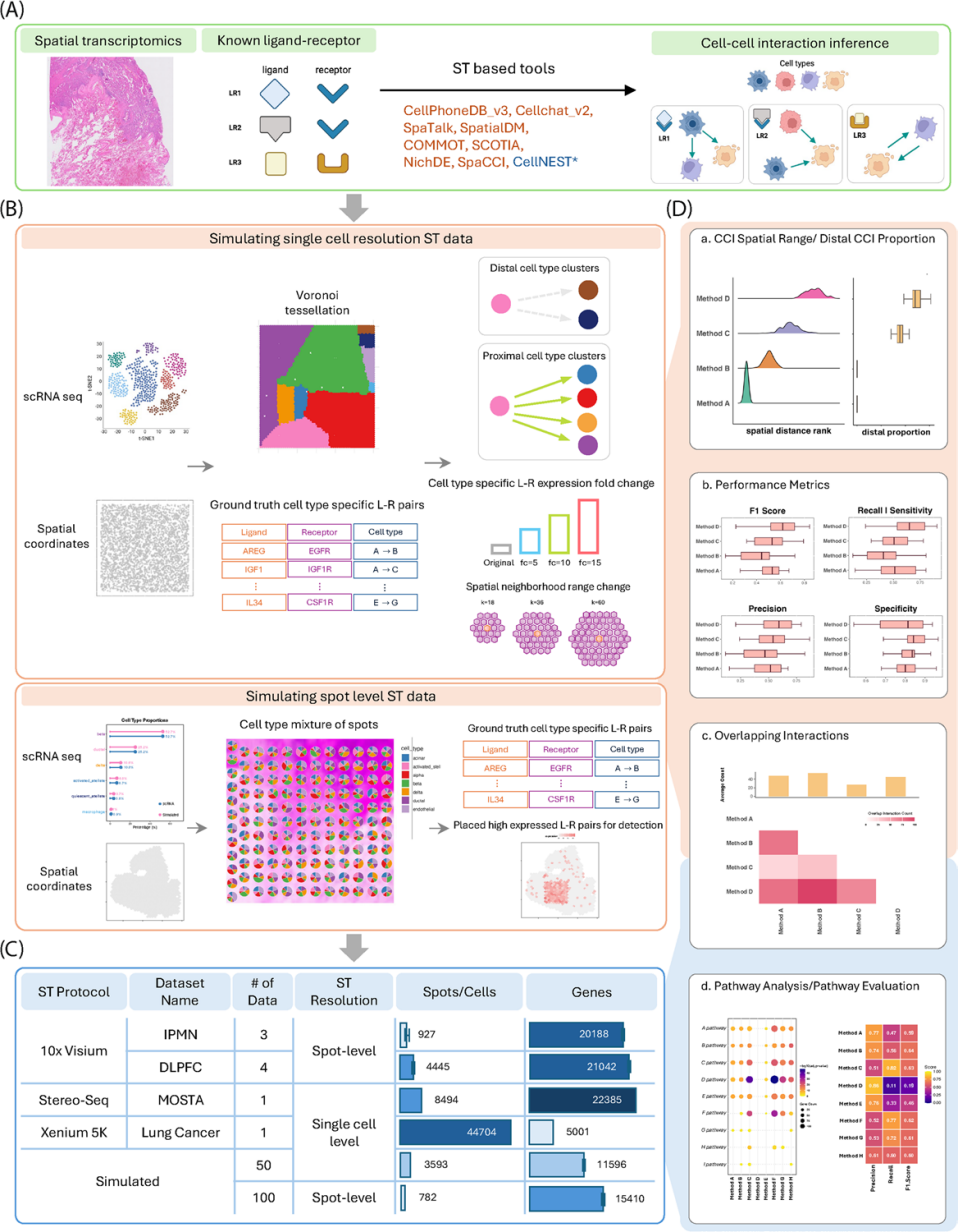
Benchmarking pipeline and summary characteristics of the datasets examined. **A** Overview of the benchmarking design, including the data types, reference knowledge, and the nine tools evaluated for cell–cell interaction performance in this study. **B** Schematic illustration of the simulation framework used to generate spatial transcriptomics data at both single-cell and spot-level resolutions. **C** Summary of the real datasets used in this study, detailing the spatial technology, number of spots or cells, and number of genes; bar plots represent mean values. **D** Overview of the performance evaluation strategies. For simulated data, we assess spatial interaction range distribution, ground truth recovery, and overlapping interactions. For real datasets, we evaluate overlapping interactions and biologically meaningful pathway support. (*CellNEST was not included in the simulation benchmark because it does not infer cell-type-specific ligand–receptor interactions; therefore, it was excluded from simulation-based comparisons but included in real-data analyses)

To evaluate the performance of CCI inference methods for analyzing ST data, we designed a comprehensive benchmarking framework that incorporates both simulated and real datasets (Fig. 1). We assessed nine CCI inference methods including CellPhoneDB v3, CellChat v2, SpaTalk, SpatialDM, COMMOT, SCOTIA, NicheDE, SpaCCI and CellNEST, which represent a range of statistical modeling and computational strategies (Table 1; Fig. 1A). All of the benchmarked spatial methods were developed on or after 2022, and most of them primarily rely on statistical testing frameworks to detect significant cell-type-specific L-R interactions.

**Table 1:** Representative computational tools for inferring cell-cell interactions

Tool	Year	Platform	Data input	Inference	Method	Scale	Strength score	Knowledge	Handling single-cell level	Handling spot level	Result output
CellChat v2	2024	R	scRNA ST	Cell type to cell type	Communication probability from L-R expression; permutation test	Global	Interaction strength	LR database	V	Treat spot as a single cell type	Chord diagram, Heatmap, Dotplot, Riverplot, Signalling network output
CellPhoneDB v3	2023	Python	scRNA ST	Cell type to cell type	Mean L-R expression; permutation test	Global	Interaction score	LR database	V	Treat spot as a single cell type	Interaction table, Heatmap, Dot plot
SpaTalk	2022	R	ST	Cell type to cell type	Graphical network with weighted L-R expression; permutation test	Local	Interaction score	LR database	V	Deconvolute spots, but treat a spot as a single cell type	Interaction table, LR expression, Pathway analysis
SpatialDM	2023	Python	ST	Gene to gene (cluster to cell type)	Global/Local Bi-Variate Moran’s I from the expression of the L-R; permutation test/normal approximation	Global, Local	X	LR database	V	Treat spot as a single cell type	LR expression, Pathway analysis, Chord diagram
COMMOT	2023	Python	ST	Gene to gene/Cell type to cell type	Collective optimal transport; permutation test	Global, Local	Interaction score	LR database	V	Treat spot as a single cell type	Spatial direction plot, Cell type interaction network plot, DE genes detection
SCOTIA	2024	Python	ST	Gene to gene (cluster to cell type)	Unbalanced optimal transport; permutation test	Local	Interaction likelihood	LR database	V	Treat spot as a single cell type	Interaction likelihood table, Interaction plot
Niche-DE	2024	R	ST	Cell type to cell type	First identify differential genes, then use Niche-Net to identify significant L-R pairs	Local	X	LR database	V	Consider cell type mixture for the average expression, find DE genes in niches	DE genes table, Interaction table
SpaCCI	2025	R	ST	Cell type to cell type	Interaction probability from the expression of the L-R; permutation test	Global, Local, Region	Interaction strength	LR database	Could do one-hot encoding for cell types	Directly incorporate cell type mixture into modeling	Interaction table, Heatmap, Chord diagram, Interaction strength plot
CellNEST	2025	Python	ST	Cell to cell	Graph attention network (GAT) with Deep Graph Infomax (DGI) - contrastive learning	Local	Interaction score rank	LR database	V	Treat spot as a single cell type	Local direction plot, Component plot, histogram, interactive web interface

To control for heterogeneity in L-R resources across methods, we conducted benchmarking under two database configurations. In the native-DB setting, each method was evaluated using its original, method-specific L–R database, reflecting typical real-world usage. In parallel, we constructed a harmonized common-DB by taking the intersection of widely used L–R databases (Additional file 1: Fig. S32), ensuring that all evaluated methods operated on an identical L–R universe. All simulation experiments were performed exclusively under the common-DB setting to fully eliminate database-driven confounding. For real-data analyses, results are reported under both native-DB and common-DB configurations to assess the robustness of method performance to database choice.

Simulated ST datasets were constructed at two resolutions–spot-level and single-cell level (Fig. 1B)–to mimic the two primary types of ST technologies. Spot-level simulations utilized tissue grids based on real Visium (10x Genomics) [30] layouts, with mixtures of cell types assigned to each spot using matched reference scRNA-seq data [31] from the same tissue. Single-cell resolution simulations involved spatially arranging individual cells and embedding predefined sender-receiver cell-type interactions. Ligand and receptor expressions were simulated using scRNA-seq derived profiles with variable expression fold changes to model heterogeneity. The spatial distribution of inferred interactions was also evaluated to assess distance-dependent characteristics. Details of simulation procedures and expression settings are provided in the Methods section.

As the simulation framework defines the truth at the level of sender–receiver cell-type pairs, methods that infer interactions exclusively at the level of individual cell–cell edges without explicit cell-type–resolved interaction modeling are not directly comparable in this setting. For this reason, CellNEST, which focuses on learning interaction patterns between individual cells using deep learning and graph-based representations, was excluded from simulation-based evaluations. Instead, CellNEST was included in real-data analyses, where ground truth is unknown and biological relevance is assessed through pathway enrichment, cross-sample consistency, and reproducibility rather than direct recovery of predefined cell-type–specific interactions.

Nine ST datasets were collected from published studies. These datasets were generated generated using three widely adopted and technically distinct platforms: 10x Genomics Visium [13] (whole transcriptome, spot-level resolution), Stereo-seq [32] (whole transcriptome, single-cell resolution), and 10x Genomics Xenium Prime [33] (5K gene panel, single-cell resolution) (Fig. 1C). These platforms differ in spatial resolution, transcript detection modalities, and gene coverage, offering a diverse testbed for evaluating method performance across various biological contexts. The datasets encompass several tissue types, including pancreas, brain, embryos and lung, with cell-type annotations obtained either directly from the original studies or inferred via marker-based clustering. For Visium ST datasets, the cell-type proportion estimates were extracted from the original publications, enabling cell type aware interaction inference without requiring additional deconvolution.

Two complementary strategies were employed to evaluate method performance (Fig. 1D). In simulated datasets, performance was quantified by comparing inferred interactions with known simulation-defined truth using specificity, precision, recall, and F1 scores, with F1 scores penalizing extreme precision–recall trade-offs and remaining robust when true interacting pairs are rare, thereby providing a single summary across varying significance thresholds. In real data applications, where ground truth CCIs are not available, method performance was evaluated along two orthogonal dimensions: biological relevance and robustness across samples. Biological relevance was assessed by comparing inferred interactions to curated, literature-supported signaling pathways using pathway-level precision, recall, and F1 scores. Robustness was further evaluated by assessing cross-sample consistency of inferred interactions across biological replicates and tissue sections using overlap-based metrics. Together, these complementary evaluation strategies enable a balanced comparison of CCI methods in terms of accuracy, biological plausibility, and reproducibility. Additional details on evaluation procedures are provided in the Methods section.

The diversity of ST technologies and computational approaches is critical to advancing the field. This study embraces that principle by incorporating datasets from widely adopted platforms such as Visium ST, as well as newer, higher-resolution technologies like Stereo-seq and Xenium. While Visium ST remains a popular platform due to its commercial accessibility and broad usage in public datasets, Stereo-seq and Xenium Prime 5 K have attracted increasing attention for their ability to capture tissue architecture at substantially higher spatial resolution [34, 35]. Likewise, the evaluated CCI inference methods vary in their underlying statistical frameworks, modeling assumptions, and interpretations of L-R biology. By acknowledging the heterogeneity across both experimental platforms and analytical tools, this study highlights key limitations of current methods and offers practical guidance for tool selection and future methodological development.

### Evaluation of CCI inference methods using in silico simulation studies

We first systematically assessed the performance of CCI inference tools using synthetic ST data with predefined simulated truth interactions. Specifically, we simulated 50 single-cell-resolution ST datasets and 100 spot-level-resolution datasets, embedding known L-R interactions by increasing the expression of selected L-R gene pairs in spatially adjacent cell pairs.

**Fig. 2:**
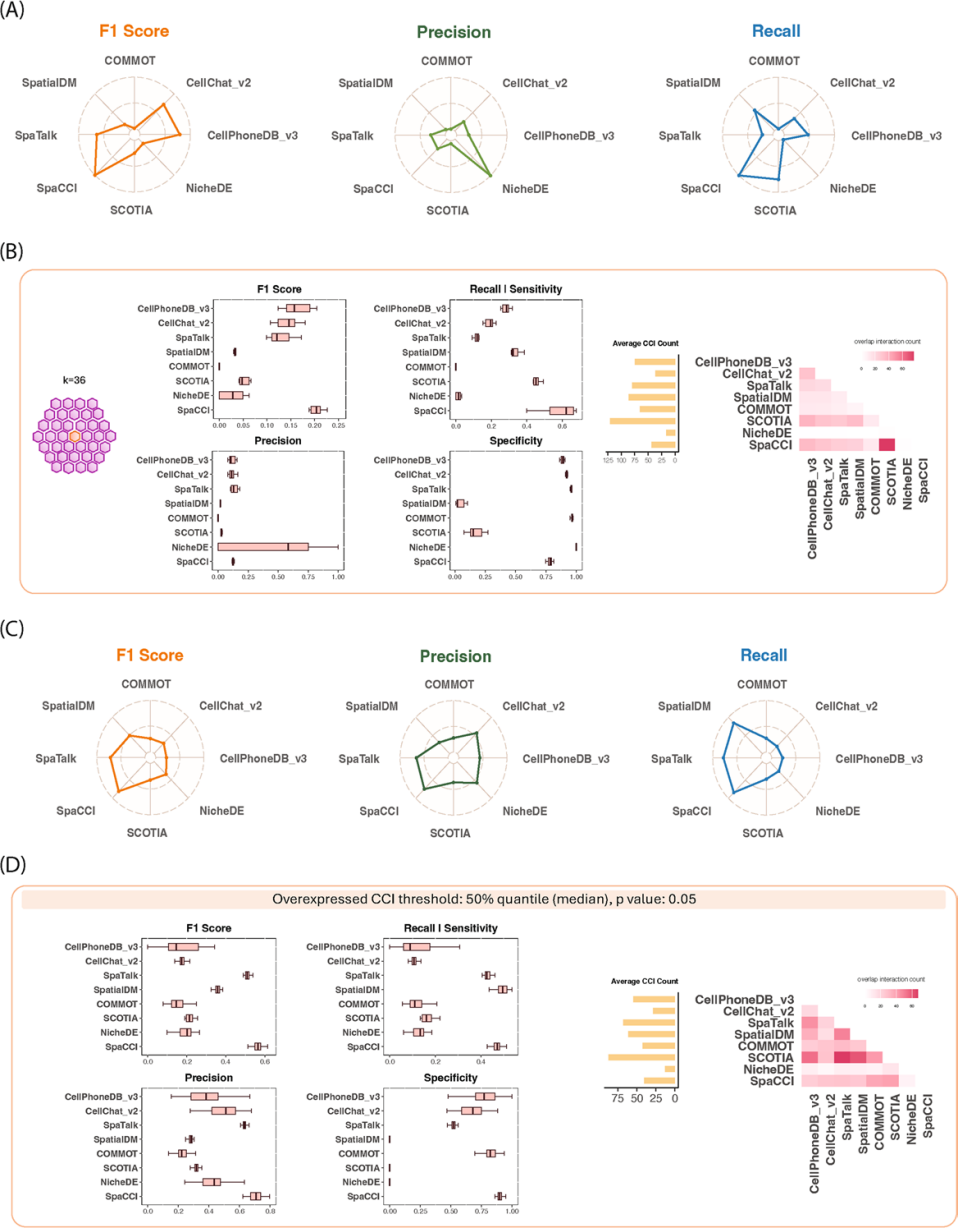
Evaluation of cell–cell interaction inference on simulated datasets. **A** Radar plots summarizing normalized mean F1 score, precision, and recall for each method based on simulations using single-cell-resolution spatial transcriptomics data across scenarios. **B** Performance under a simulation scenario with a spatial interaction range of $k=36$, where interacting cell pairs exhibit a 10-fold increase in ligand-receptor expression, serving as the simulation-defined truth. Left: boxplots showing the distribution of F1 score, recall (sensitivity), precision, and specificity for each method. Right: bar plot showing the average number of detected interactions and a heatmap of overlap counts between methods. **C** Radar plots showing normalized mean F1 scores, precision, and recall for each method on spot-level simulated data across scenarios. **D** Performance under a simulation scenario where ground truth interactions are defined as ligand-receptor pairs overexpressed above the median (50th percentile) relative to global expression, with a significance threshold of $p<0.05$ applied during analysis. Left: boxplots of F1 score, recall, precision, and specificity. Right: average number of detected interactions and overlap counts between methods

For single-cell-resolution simulation studies, interacting cell pairs were defined based on spatial proximity using a nearest-neighbors approach (k = 18, 36, 60), and L-R gene pairs were simulated with overexpression levels at 5, 10, or 15 fold relative to their original expression. Tool performance was evaluated using F1 score, precision, recall, and specificity under various statistical thresholds (*p* < 0.05, 0.1, 0.2) to assess detection sensitivity and robustness (Fig. 2A, B; Additional file 1: Figs. S2-5). To facilitate comparability across methods and thresholds, performance metrics were normalized (Fig. 2A). SpaCCI consistently achieved the highest mean normalized F1 scores (92.7%; range 86–94%), followed by CellPhoneDB v3 (77.4%; range 76–79%), CellChat v2 (69.6%; range 68–71%), and SpaTalk (62.2%; range 60–64%). In contrast, NicheDE, COMMOT, SCOTIA, and SpatialDM showed markedly lower scores ($\le 26\%$). Although absolute F1 scores were low due to signal sparsity (Additional file 1: Figs. S2–5), normalized values highlighted consistent relative performance differences among methods. Notably, NicheDE and COMMOT exhibited higher specificity (Fig. 2B) but at the cost of reduced true positives, underscoring a trade-off between minimizing false positives and recovering true interactions. Furthermore, we examine the ability of each tool to capture the spatial characteristics of interactions by ranking sender–receiver cell type pairs according to their average spatial proximity, where proximity serves as a proxy for different signaling modalities. Biologically, CCI can occur through autocrine (self-signaling) and paracrine (short-range) processes that are inherently proximal, as well as endocrine signaling that acts over long distances. Tools such as CellChat v2, SpaTalk, SpatialDM, NicheDE, and SpaCCI predominantly detected proximal interactions, whereas CellPhoneDB v3, COMMOT, and SCOTIA more frequently identified interactions across distal cell populations (Additional file 1: Fig. S1).

In the spot-level simulations, simulation-defined truth interactions were defined by joint overexpression of L-R pairs exceeding global quantile thresholds (25th, 50th, and 75th percentiles), mimicking realistic levels of sparsity in spot-level ST signals. Performance was similarly evaluated across multiple significance thresholds(0.05, 0.1, 0.15 and 0.2), and while detection results fluctuated depending on the quantile and significance cut-off used, SpaCCI and SpaTalk consistently outperformed other methods. SpaCCI achieved a mean normalized F1 score of 82.1% (range 54–97%) and SpaTalk reached 72.4% (range 41–90%), representing more than a twofold increase compared to other methods. SpatialDM achieved moderate performance with a mean normalized F1 score of 49.6% (range 21–67%), while all remaining methods were substantially lower ($\le 35\%$). Both SpaCCI and SpaTalk also attained the highest precision and recall across all tested scenarios (Fig. 2C, D; Additional file 1: Figs. S6-7).

As spot-level real ST data often contain mixtures of multiple cell types (Additional file 1: Fig. S39), we additionally conducted a dedicated set of spot-level simulations to evaluate how spot purity influences CCI inference performance. These simulations were designed to systematically vary cell-type composition while preserving the same interaction-generation principles used in the spot-level framework. Details of the simulation design and evaluation procedures are also provided in the Methods section. We compared a high-purity regime, in which the dominant cell type accounted for over 80% of cells within the interacting spots, with a mixed(low)-purity regime in which the dominant cell type represented less than 80% of cells. Across both purity regimes, the relative performance ordering of methods remained highly consistent (Additional file 1: Fig. S40), indicating that performance differences were largely preserved across purity conditions. Notably, methods that explicitly model cell-type mixtures or incorporate deconvolution, including SpaCCI and SpaTalk, showed slightly improved performance under mixed-purity conditions, whereas methods that assign each spot to a single dominant cell type exhibited modestly higher performance under high-purity conditions. Importantly, no method displayed artificial performance inflation with increasing purity, and methods that failed to recover simulation-defined interactions under mixed-purity conditions also failed under high-purity settings.

Resolution-dependent differences were evident when comparing single-cell and spot-level simulations. The heatmaps (Fig. 2B, D) illustrate the degree of concordance in detected interactions between methods, where higher overlap indicates greater agreement and lower overlap indicates divergence; NicheDE consistently showed the lowest concordance across both resolutions. The accompanying barplots show that single-cell simulations yielded a larger number of detected CCIs than spot-level simulations, reflecting the richer information content at higher resolution. Comparing radar plots (Fig. 2A vs. C), CellPhoneDB v3 and CellChat v2 performed better in single-cell simulations, whereas SpaCCI and SpaTalk maintained consistently high performance across both resolutions.

### Evaluation of comparison methods on Visium ST studies

As Visium ST remains one of the most widely adopted ST profiling technologies to date, we assessed method performance using two publicly available Visium ST datasets. The first dataset comprises tissue slices from pancreatic preneoplastic lesions, specifically intraductal papillary mucinous neoplasms (IPMNs) and IPMN-associated pancreatic cancer [36]. We utilized three tissue slides (one low-grade IPMN, one high-grade IPMN, and one IPMN-associated pancreatic cancer). Each tissue slice contains approximately 930 spots and over 20,000 detected genes. The second dataset includes four tissue slices from the dorsolateral prefrontal cortex [37, 38] (DLPFC) of a healthy donor, with each slice comprising over 4,400 spots and more than 21,000 detected genes.

**Fig. 3:**
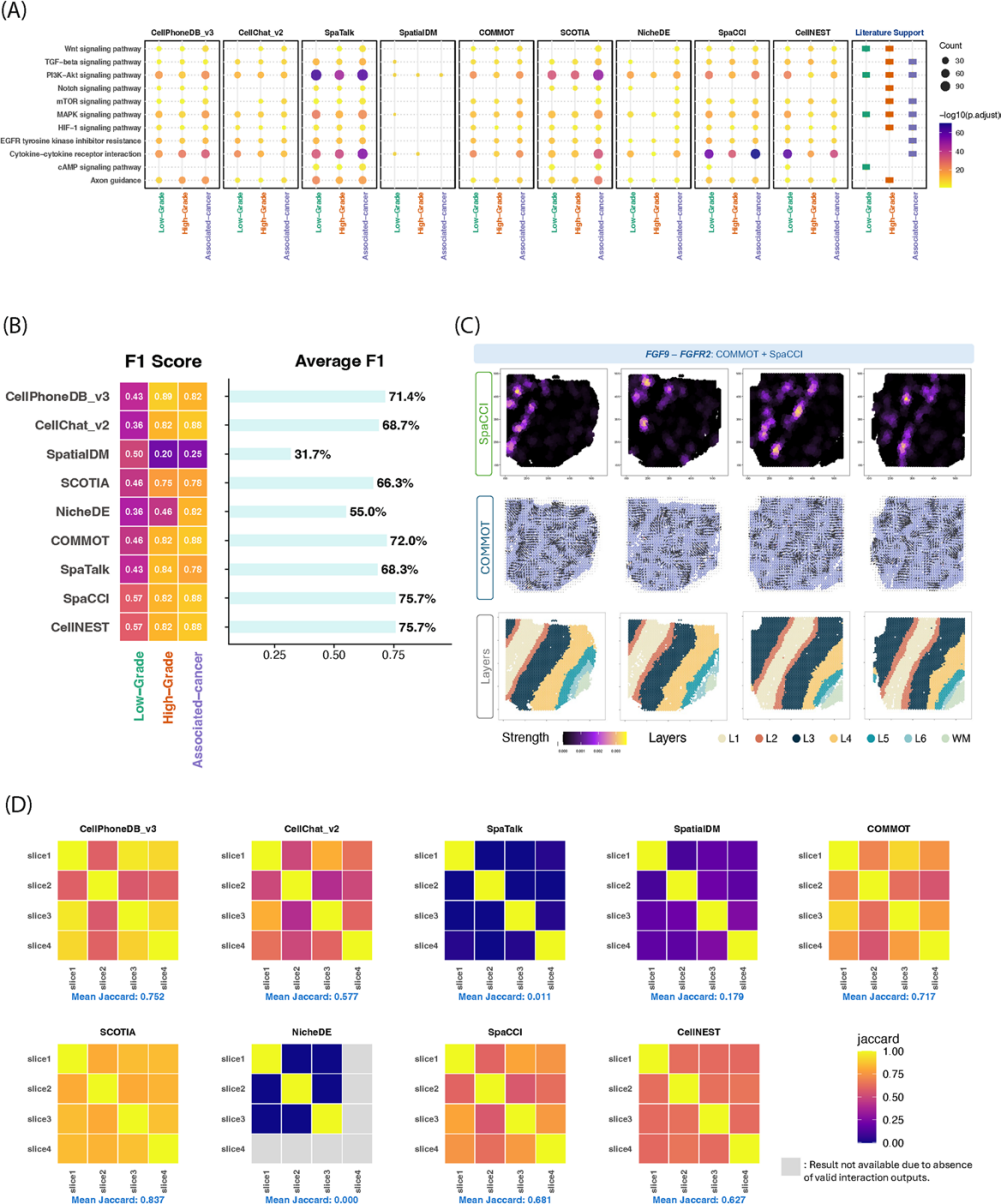
Evaluation of cell–cell interaction inference on 10x Visium datasets. **A** Pathway enrichment analysis of inferred interactions across IPMN progression stages, as identified by eight computational CCI tools. The panel on the right indicates literature-supported pathways for each condition, providing a reference for biological relevance. **B** Heatmap showing the F1 score for each condition and tool, based on pathway enrichment analysis on the IPMN dataset. F1 scores reflect the agreement between inferred and literature-supported pathways. The accompanying bar plot summarizes the average F1 score across conditions for each method. **C** Local signaling strength (SpaCCI) and inferred directionality (COMMOT) for the FGF9 - FGFR2 interaction in the DLPFC dataset across four slices. **D** Consistency evaluation across four DLPFC slices for each tool, measured by pairwise Jaccard indices of detected interactions. Mean Jaccard index is reported for each tool. The absence of valid CCI result was annotated as grey (i.e. Slice 4 by NicheDE). (Note: All four DLPFC slices originate from the same donor sample)

No existing publication provides a comprehensive list of CCIs present in the studied tissues. To evaluate biological relevance, we compiled literature-supported signaling pathways specific to each tissue context as a silver standard and compared them with the biological processes associated with the inferred CCIs (Additional file 2: Tables S1-S2). In the IPMN dataset, for instance, the cAMP signaling pathway has been previously reported to be active predominantly in low-grade lesions [39], while TGFB signaling has been found to be activated in both high-grade [40] and associated pancreatic cancer lesions [41, 42] (Fig. 3A). In addition, interactions involving T cells or B cells are known to be enriched in IPMN tissue [40, 42]; however, not all comparison methods can successfully recover them (Additional file 1: Fig. S15B). Under native-DB settings, methods showed variability in agreement with literature-supported pathways (Fig. 3B). Based on average F1 scores across conditions, CellNEST (75.7%), SpaCCI (75.7%), COMMOT (72.0%), and CellPhoneDB v3 (71.4%) achieved the highest concordance, whereas SpatialDM showed markedly lower performance (31.7%). Other methods displayed intermediate performance, reflecting differences in sensitivity and pathway coverage. When restricting analyses to a harmonized common-DB, most methods exhibited reduced average F1 scores, consistent with the smaller interaction space (Additional file 1: Fig. S35B). In the IPMN dataset, SpaCCI and SpaTalk achieved the highest average F1 scores (both 61.0%), whereas COMMOT and SpatialDM returned no valid predictions under this setting (Additional file 1: Figs. S34B, S35B).

In the DLPFC dataset, we also assessed agreement with literature-supported pathways across multiple slices from the same donor. Under native-DB settings, CellPhoneDB v3 (49.4%), SpaCCI (49.2%), CellNEST (43.6%), and SCOTIA (40.9%) showed the strongest agreement with literature-supported pathways (Additional file 1: Fig. S36B). In common-DB analyses, average F1 scores decreased across most methods, but SpaCCI (48.3%), SCOTIA (51.3%), and CellNEST (43.4%) retained relatively higher pathway-level F1 scores across slices compared with other approaches (Additional file 1: Fig. S36D). For some method–dataset combinations, no true positives were detected under the defined silver-standard criteria, resulting in zero precision and recall and consequently an F1 score of zero. These cases are shown as blank entries in the corresponding heatmaps and reflect limitations in sensitivity under restricted interaction settings rather than numerical artifacts.

To address robustness for each method, we evaluated the consistency of inferred CCIs across biological replicates and tissue sections independent of pathway enrichment. Consistency was quantified using pairwise Jaccard indices, which measure the overlap of inferred L-R interactions between samples. In the DLPFC dataset, four spatial transcriptomic slices were derived from the same donor, with two slices sampled from the same anatomical location and the remaining two separated by approximately 300 $\upmu$m. We therefore assessed cross-slice reproducibility of inferred CCIs by computing pairwise Jaccard overlap across all slice pairs (Fig. 3D; Additional file 1: Fig. S36A, B). Under native-DB settings, spatially aware methods, including SCOTIA (mean Jaccard: 83.7%), CellPhoneDB v3 (75.2%), COMMOT (71.7%), SpaCCI (68.1%), and CellNEST (62.7%), exhibited higher cross-slice overlap compared with other approaches, indicating more reproducible interaction inference across adjacent tissue sections. When restricting analyses to the harmonized common-DB, several methods, including SpaCCI, SCOTIA, and CellNEST, maintained comparable or improved relative Jaccard consistency after controlling for database size and interaction count, whereas CellPhoneDB v3 showed a reduction in overlap (Additional file 1: Fig. S36B). In contrast, methods that failed to produce valid interaction calls in multiple slices yielded zero or undefined overlap values.

We performed an analogous consistency analysis in the IPMN dataset by evaluating interaction overlap and pathway-level F1 scores across multiple samples within the same pathological grade (LG1–3, HG1–3, PDAC1–3; Additional file 1: Figs. S33–34). This analysis tests whether methods consistently recover similar interaction patterns within the same disease state. Under native-DB settings, spatially informed methods generally showed stronger within-grade overlap. In low-grade lesions, SpaCCI and SpaTalk achieved the highest mean Jaccard overlap (36.2% and 34.7%, respectively), while in high-grade samples SpaCCI (33.7%) and SCOTIA (37.5%) showed the strongest consistency. In associated cancer samples, SCOTIA demonstrated the highest overlap (67.5%), followed by SpaTalk (60.8%) and SpaCCI (49.8%) (Additional file 1: Fig. S33A). Pathway-level agreement further supported these patterns. Across pathological grades, SpaCCI consistently achieved among the highest average F1 scores (49.8% in low-grade, 79.9% in high-grade, and 87.5% in associated cancer), indicating stable recovery of biologically relevant interactions across samples within the same disease state. SpaTalk and CellNEST similarly maintained high and relatively stable F1 scores across grades, whereas several methods exhibited greater grade- or sample-specific variability or no detected CCIs in certain samples, leading to undefined overlap or F1 scores (Additional file 1: Fig. S33A, B). When analyses were restricted to the harmonized common-DB, interaction overlap and pathway-level F1 scores were often reduced due to the smaller interaction space and increased sparsity imposed by database intersection. However, relative consistency patterns were largely preserved among methods that produced valid interaction inferences, and in some cases overlap or F1 scores were maintained or increased, reflecting reduced database-driven variability and increased emphasis on interactions consistently supported across methods. In particular, SpaCCI remained comparatively robust, achieving mean Jaccard overlaps of 35.1% in low-grade lesions and 45.6% in associated cancer, together with average F1 scores of 53.9% and 79.1%, respectively (Additional file 1: Fig. S34). Several approaches produced insufficient interactions for evaluation in specific grades under the intersected database, resulting in undefined overlap or F1 scores (shown as blank entries), which reflect method-specific sensitivity to LR sparsity rather than numerical artifacts.

To illustrate method-specific capabilities for various downstream analysis tasks, we visualized localized interaction hotspots and directional signaling flows. In low-grade IPMN tissue, SpaCCI identified distinct epithelial-directed SPP1-CD44 interaction, including epithelial to T cell, consistent with the known role of SPP1-CD44 signaling in immune modulation and tumor-associated macrophage recruitment [43]. Meanwhile, COMMOT captured a broad epithelial-originating directional pattern, highlighting consistent communication from epithelial cells to surrounding compartments (Additional file 1: Fig. S15A). In the DLPFC dataset, the FGF9-FGFR2 interaction, which is important for cortical development [44, 45], was examined across four tissue slices (Fig. 3C). COMMOT revealed a consistent global flow of directional signaling, while SpaCCI detected highly localized interaction hotspots, particularly enriched between cortical layers L2 and L3, highlighting the complementary strengths of the two approaches.

### Evaluation of comparison methods using a Stereo-seq study

**Fig. 4:**
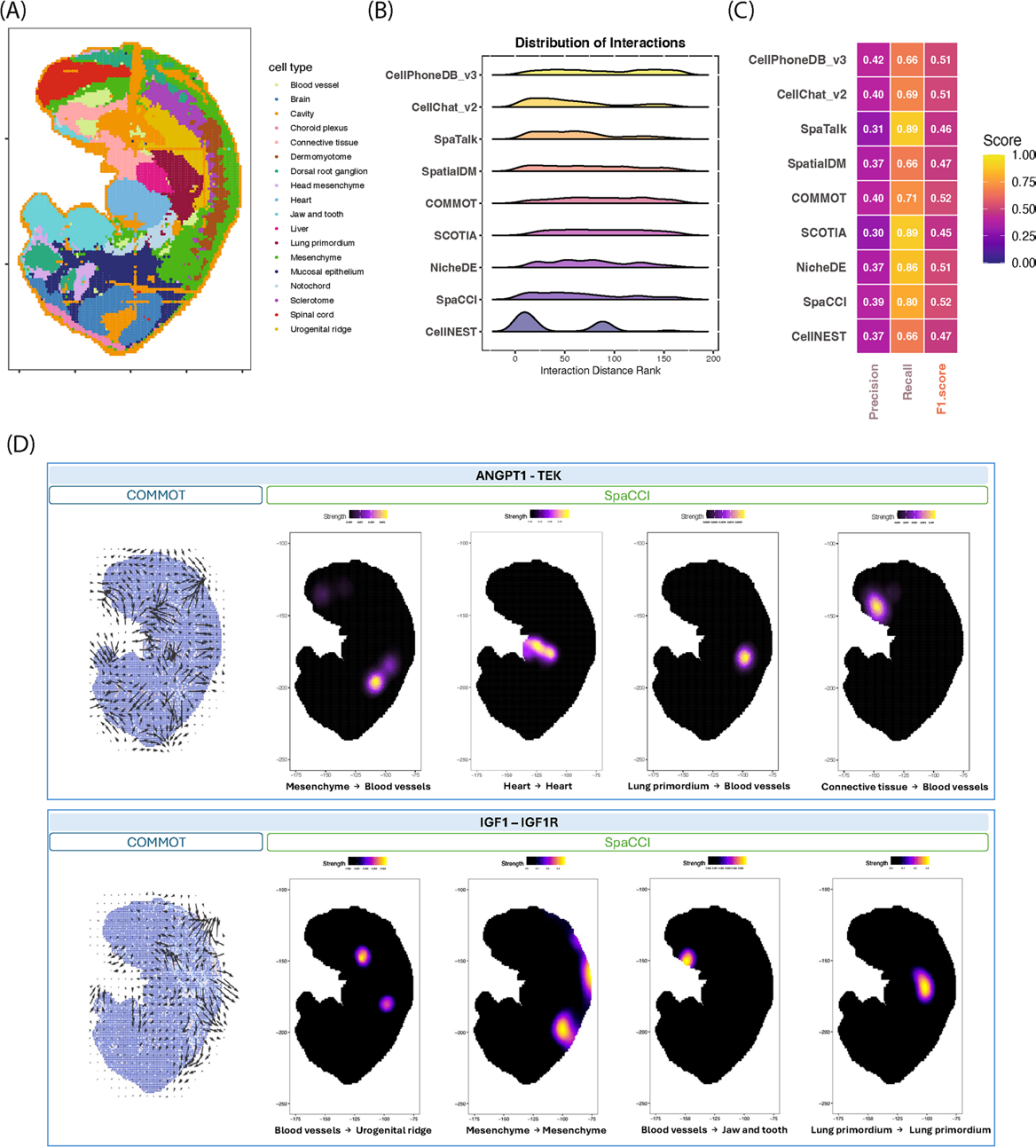
Evaluation of cell–cell interaction inference on the Stereo-Seq dataset. **A** Cell type annotation of the Mouse Organogenesis Spatiotemporal Transcriptomic Atlas (MOSTA) at E10.5. **B** Ridge plots showing the distribution of interaction distances inferred by each tool. On the x-axis, lower ranks indicate proximal interactions between cell types, while higher ranks reflect distal interactions. **C** Heatmap summarizing performance metrics (precision, recall, and F1 score) for each tool based on pathway enrichment analysis. F1 scores reflect the agreement between pathways inferred by each tool and literature-supported pathways in the E10.5 mouse embryo. **D** Spatial visualization of selected interactions. Top panel: ANGPT1 - TEK interaction, showing global directionality (COMMOT) and local cell-type-specific signaling strength (SpaCCI). Bottom panel: IGF1 - IGF1R interaction, with similar visualization of directionality and strength

All methods were applied to high-resolution spatial transcriptomics data collected on mouse embryo (MOSTA) at E10.5 from Stereo-seq [32], where the cell type annotation is provided from the previous publication [32]. This dataset comprises over 8,000 cells and 18 annotated cell types (Fig. 4A), with expression measured in more than 20,000 genes.

We assessed the interaction distance distributions by ranking sender–receiver cell type pairs based on their average spatial proximity (Additional file 1: Fig. S25B). Although all methods yielded a broad range of interacting distances (Fig. 4B), CellNEST, CellChat v2, SpaTalk, NicheDE, and SpaCCI showed a tendency to prioritize more proximal interactions.

To evaluate the biological concordance between the tool-identified CCI signals and the published evidence, we compared the detected interactions against literature-supported pathways known to be active during mouse embryonic development at E10.5 (Additional file 2: Table S3). Based on F1 score performance (Fig. 4C; Additional file 1: Fig. S37A), SpaCCI (0.52) and COMMOT (0.52) showed the highest agreement with known developmental signaling pathways under native-DB settings, followed closely by NicheDE (0.51) and CellPhoneDB v3 (0.51). SpaCCI and COMMOT achieved recall values of 0.80 and 0.71, respectively, whereas SpaTalk and SCOTIA reached higher recall values approaching 0.89. However, SpaTalk and SCOTIA exhibited substantially lower precision (approximately 0.30), which reduced their overall F1 scores despite high recall. In contrast, SpaCCI and COMMOT maintained higher precision (approximately 0.39–0.40), leading to improved F1 performance even though their recall values were lower than those of SpaTalk and SCOTIA. When analyses were restricted to common-DB (Additional file 1: Fig. S37C), the total number of detected interactions decreased across all methods, reflecting the reduced interaction space. However, F1 score performance did not uniformly decline. Instead, several methods, including SpaCCI (0.53), NicheDE (0.53), SpaTalk (0.52), and CellNEST (0.52), maintained or modestly improved F1 scores, indicating robustness of pathway recovery under database harmonization through reduced false-positive expansion, rather than reliance on database-specific interaction expansion. For CellNEST, which outputs ranked interaction confidence scores, we additionally evaluated multiple confidence thresholds to assess robustness of performance to threshold selection. Across both native-DB and common-DB settings, F1 scores remained stable over a wide range of thresholds (Additional file 1: Fig. S37B, D), indicating that relative performance was not sensitive to the specific cutoff chosen.

Beyond global performance metrics, method-specific downstream analyses were illustrated through directional flow and localized interacting hotspot visualizations (Fig. 4D). For the ANGPT1-TEK interaction, known to regulate vascular morphogenesis [46, 47], COMMOT effectively captured broad signaling directionality across the embryonic body axis. In contrast, SpaCCI identified focused interaction hotspots between specific cell types, such as mesenchyme and blood vessels. Similarly, for IGF1-IGF1R signaling, which is a key pathway in developmental growth, COMMOT highlighted global signaling directional patterns, while SpaCCI revealed cell-type specific interaction zones in lung primordium [48] and developing tissues [49, 50]. These complementary visualizations underscore the distinct analytical strengths of each method.

### Evaluation of comparison methods on Xenium Prime 5 K data

**Fig. 5:**
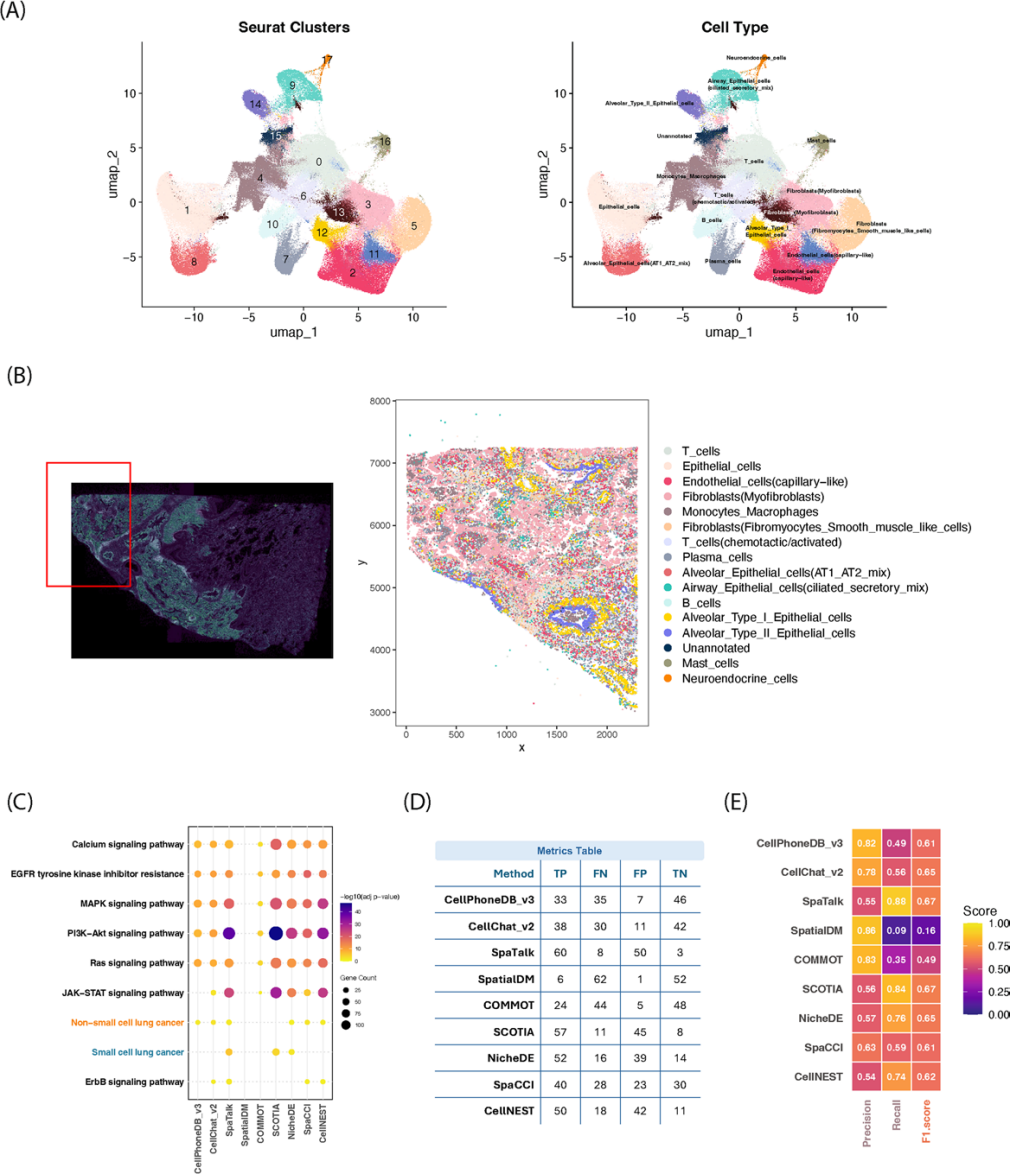
Evaluation of cell–cell interaction inference on the Xenium Prime 5 K FFPE lung cancer dataset. **A** UMAP visualization of the Xenium Prime 5 K lung cancer FFPE dataset. The left panel shows Seurat cluster assignments, while the right panel displays cell type annotations. **B** Visualization of the analyzed tissue region. Due to tool-specific limitations with large-scale data, a cropped region (highlighted in red) was selected to ensure compatibility across all nine CCI tools. **C** Pathway enrichment analysis of inferred interactions. Cancer-relevant pathways, including non–small cell lung cancer (NSCLC) and small cell lung cancer (SCLC), are highlighted. NSCLC is emphasized due to its relevance to the Xenium lung cancer sample. **D** Performance summary table reporting true positives (TP), false negatives (FN), false positives (FP), and true negatives (TN) for each method, based on comparison to literature-supported pathways. **E** Heatmap summarizing precision, recall, and F1 score for each tool, derived from pathway enrichment evaluation against literature-supported pathways

Xenium is a newly developed image-based, high-plex in situ platform capable of capturing transcriptomic signals at subcellular resolution. Compared to earlier image-based technologies with limited gene panels, the Xenium Prime 5 K platform enables quantification of up to five thousands of genes simultaneously, making it well-suited for high-resolution cellular analysis and studying complex tissues, including tumors. To evaluate method performance on this emerging platform, a Xenium Prime 5 K dataset of formalin-fixed paraffin-embedded (FFPE) human lung cancer tissue [51] was analyzed. The dataset includes expression profiles for 5,001 genes across more than 250,000 spatially resolved cells. Cell types were annotated based on marker gene expression within Seurat-defined clusters, resulting in the identification of 15 distinct cell types (Fig. 5A, Additional file 1: Fig. S28B).

Large-scale ST dataset, such as this Xenium Prime 5 K sample, presents unique computational challenges, particularly for methods that are not optimized for high cell counts. For example, SpaTalk failed to process the full dataset due to scalability limitations in its implementation, which currently restricts analyses to fewer than 50,000 cells. To allow consistent evaluation between tools, a subregion with various cellular compositions was cropped from the original tissue based on tumor location inferred from fluorescence imaging (Fig. 5B). Distance rank profiles were computed for each method to evaluate whether tools preferentially detected proximal versus distal interactions. In the Xenium Prime 5 K dataset, however, these profiles were nearly identical across methods, indicating little difference in how interaction distances were prioritized (Additional file 1: Fig. S30A, B).

To assess biological relevance, detected interactions were compared against curated pathways relevant to non-small cell lung cancer (NSCLC) (Additional file 2: Table S4). Notably, only CellPhoneDB v3, CellChat v2, SpaCCI and CellNEST independently identified NSCLC-associated pathway through their CCI signaling lists, whereas SpaTalk detected signaling pathways of both NSCLC and small cell lung cancers (Fig. 5C). Under native-DB settings, SpaTalk and SCOTIA achieved the highest F1 scores (both 0.67), reflecting strong recovery of literature-supported NSCLC signaling pathways (Fig. 5D, E; Additional file 1: Fig. S38A). These high F1 scores were driven primarily by elevated recall (0.88 and 0.84, respectively), whereas methods such as SpatialDM and COMMOT exhibited substantially lower recall (0.09 and 0.35, respectively), resulting in reduced overall F1 performance (0.16 and 0.49, respectively). SpaCCI and CellNEST achieved intermediate F1 scores (0.61–0.62), maintaining moderate precision (0.63–0.54) and recall (0.59–0.74) without the strongly recall-dominated behavior observed in SpaTalk and SCOTIA. When analyses were restricted to the harmonized common-DB, the total number of detected interactions decreased across all methods (Additional file 1: Fig. S38C), consistent with the reduced interaction space. Despite this reduction, relative F1 score rankings were largely preserved. Several methods, including SpaCCI (0.80), SCOTIA (0.79), CellNEST (0.76) and NicheDE (0.74), achieved high F1 scores by simultaneously retaining both precision and recall. Notably, SpaCCI attained the highest F1 score among all evaluated approaches, reflecting robust recovery of NSCLC-associated signaling under stringent database harmonization. For CellNEST, which outputs ranked interaction confidence scores, we further examined sensitivity to confidence thresholding in the large-scale Xenium setting. Across both native-DB and common-DB settings, F1 scores remained stable over a wide range of thresholds (Additional file 1: Fig. S38B, D), indicating that relative performance was not sensitive to threshold choice.

### Summary of performance and guidelines for method selection

**Fig. 6:**
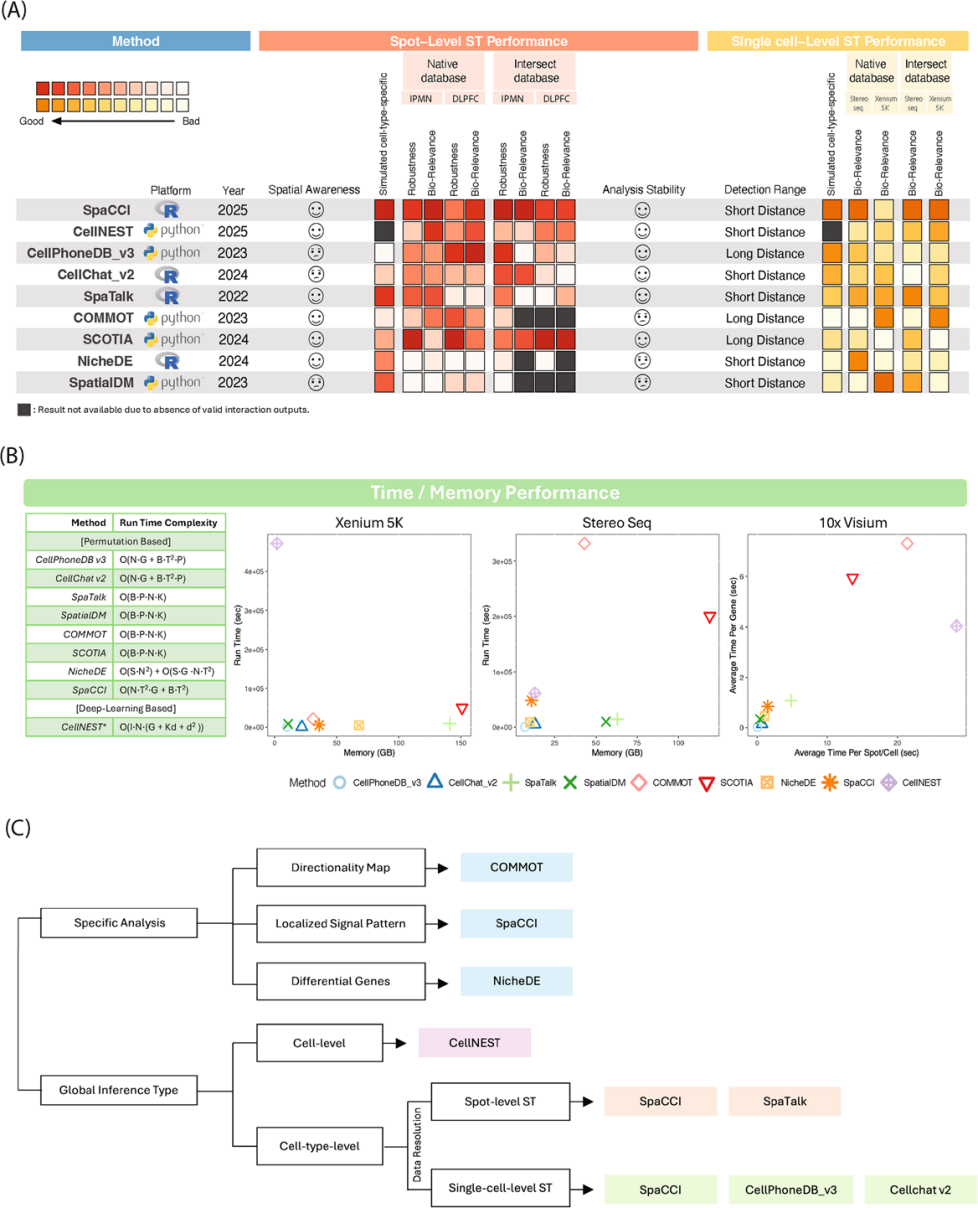
Summary of overall performance and usage guidelines for cell–cell interaction inference methods. **A** Overview of platform (Python or R), release year, spatial awareness in spot-level ST data, and benchmarking performance across simulated and real datasets. Performance is shown separately for spot-level ST data (red scale) and single-cell ST data (yellow scale). Darker colors indicate better performance. Grey color represent the absence of valid result. **B** Runtime and memory usage comparison across datasets. Left: Asymptotic run-time complexity for each permutation based method, using N (cells/spots), T (cell types), P (ligand–receptor pairs), B (permutations), K (neighbors per cell), G (genes), and S (kernel bandwidths; NicheDE). For deep learning based method CellNEST, runtime additionally depends on the d (embedding dimension) and I (the number of training iterations). Middle: Wall-clock runtime versus peak memory on the Xenium Prime 5 K lung cancer dataset and on the MOSTA Stereo-seq dataset. Right: average runtime per spot/cell versus average runtime per gene, summarizing method efficiency at the per-observation and per-gene levels on Visium datasets. **C** Scenario-specific, decision-tree-style guideline summarizing key strengths of each method. Specific analyses include directionality, localized signaling, and differential gene inference. Global analyses are divided by inference type (cell-level vs. cell-type-level) and resolution (spot-level vs. single-cell)

We summarized the features and overall performance of nine CCI inference methods across both spot-level and single-cell ST data (Fig. 6A). Overall, method performance exhibited clear resolution-dependent patterns rather than a single universally optimal approach. SpaCCI showed consistently robust and consistent performance across simulated and real datasets, particularly in spot-level ST settings where cell-type mixtures and spatial localization are critical, reflecting its explicit modeling of cell-type composition and spatial context. However, this consistency does not imply universal superiority; rather, it reflects strong performance under data regimes aligned with SpaCCI’s modeling assumptions. CellPhoneDB v3 and CellChat v2 performed particularly well on single-cell–resolution ST data, including Stereo-seq and Xenium, as well as in single-cell cell-type–specific simulation studies, but showed reduced performance in spot-level settings where cell-type mixing is prevalent. In contrast, SpaTalk demonstrated stronger performance in spot-level ST analyses, achieving high accuracy in spot-level cell-type–specific simulations and robust biological relevance in Visium datasets, consistent with its spatial modeling framework. SCOTIA also showed competitive pathway-level performance in some spot-level real datasets; however, this performance was accompanied by a large number of inferred interactions compared to other methods. Across real ST datasets, biological relevance was evaluated at the pathway level for all methods. Within this framework, CellNEST showed strong performance in real ST datasets, but was not assessed in cell-type–specific simulation benchmarks due to its cell-level formulation. We further evaluated computational efficiency by comparing runtime and memory usage across three representative datasets (Xenium Prime 5 K, Stereo-seq, and 10x Visium) (Fig. 6B). CellPhoneDB v3 and CellChat v2 demonstrated favorable computational efficiency, combining relatively low runtime and memory usage across platforms, making them practical choices for large-scale single-cell ST analyses. In contrast, SCOTIA and COMMOT required substantially greater computational resources, particularly for large single-cell datasets, limiting their scalability despite strong performance in specific analytical tasks.

To facilitate practical method selection, we provide a tree-structured guideline summarizing the strengths and intended use cases of each method (Fig. 6C). This includes support for directional signaling inference (e.g., COMMOT), localized CCI hotspot detection (e.g., SpaCCI), differential gene or niche analysis (e.g., NicheDE), and global interaction inference at either cell-level or cell-type–level resolution. Together, these results emphasize that optimal method choice depends on data resolution, biological question, and computational constraints.

## Discussion

In this study, we conducted a comprehensive evaluation of nine computational methods for inferring CCIs using ST data derived from diverse spatial platforms, including in silico simulations, Visium ST, Stereo-seq, and Xenium. Our evaluation framework considered multiple aspects of performance, including detection accuracy with respect to simulation-defined cell-type specific interactions, consistency across biological replicates and pathological grades in cancer, spatial distance characteristics of inferred interactions, and the ability to recover biologically meaningful and spatially plausible interactions such as proximal signaling or known immune communication patterns. This comprehensive framework provides guidance for researchers seeking to select appropriate CCI inference tools tailored to the resolution and biological context of their ST data.

Our results demonstrate that no single method is universally optimal across all datasets, spatial resolutions, and evaluation criteria. Instead, method performance exhibits clear resolution-dependent and context-dependent trade-offs, which is highly contingent on factors such as spatial granularity, cell type composition, and underlying data sparsity. Methods originally developed for single-cell–resolution data tend to perform better when cell identities are well resolved, whereas approaches that explicitly model spatial neighborhoods or cell-type mixtures show increased robustness in spot-level settings where cellular heterogeneity is intrinsic. These findings emphasize that differences in modeling assumptions and target resolution, rather than overall methodological complexity, largely determine performance across spatial contexts.

An important practical insight from our benchmarking is the substantial influence of L-R database choice on CCI inference outcomes. Comparing native method-specific databases with a harmonized common database revealed that database composition affects performance not only by altering the size of the interaction search space, but also by exposing method-specific precision–recall trade-offs. In several cases, restricting analyses to the common-DB universe reduced database-driven false-positive expansion and resulted in more stable or even improved pathway-level concordance. However, this constraint also amplified sensitivity differences across methods, resulting in reduced recall or complete loss of detectable interactions for some approaches. These observations indicate that database harmonization does not uniformly improve precision–recall balance, but rather provides a stricter and more informative test of methodological robustness, particularly for comparative benchmarking and cross-study integration.

Beyond the overall performance metrics, several notable methodological behaviors emerged from our analysis. First, SpaTalk was found to detect interactions predominantly between different cell types, with limited ability to capture intra-lineage communication. This cell-type-specific restriction may be rooted in its model design, which emphasizes heterotypic interactions. Second, despite not directly incorporating spatial coordinates, CellPhoneDB v3 and CellChat v2 achieved better recovery of biologically meaningful interactions across multiple real datasets. This performance highlights the strength of its underlying interaction database and statistical framework, expression-driven approaches can remain effective in identifying relevant signaling patterns when cell-type organization aligns with tissue microenvironment structure. Third, NicheDE demonstrated high true positive rates across simulations and real data applications but often failed to recover the full set of expected interactions and biologically meaningful pathways. This is likely due to its two-step design, which prioritizes differential expression testing as a prerequisite for L-R evaluation. While this conservativeness limits sensitivity, it may provide complementary value when paired with broader but less specific tools. Fourth, although SpatialDM provides cell-type-specific interaction inference and incorporates statistical significance testing through permutation-based or z-score–based procedures, its inference framework is centered on detecting spatial co-expression of L-R gene pairs rather than hypothesis testing explicitly conditioned on predefined sender–receiver cell-type pairs. As a result, in spot-level ST data, the method may aggregate multiple cell-type combinations present within the same spatial unit, which can increase the number of reported interactions and reduce specificity in settings with substantial cell-type mixing. Finally, CellNEST differs conceptually from the other eight evaluated methods in that it does not rely on explicit hypothesis testing or *p*-value–based significance thresholds. Instead, it outputs ranked interaction confidence scores learned through a deep learning framework. Consequently, interaction selection depends on rank-based filtering rather than statistical significance testing. Collectively, these observations highlight the diversity of methodological assumptions and underscore the importance of aligning tool selection with the specific biological question, tissue architecture, and data resolution at hand.

Our results also clarify an important conceptual question regarding the role of spatial awareness in CCI inference. While non-spatial methods can perform well in recovering biologically meaningful signaling pathways, especially in real datasets dominated by strong expression-driven signals, spatial awareness does not automatically confer superior performance. Instead, its value depends on how spatial information is incorporated and on the resolution of the data. Spatial modeling becomes particularly important in settings where expression-only signals are insufficient, such as spot-level data with mixed cell-type composition, under stringent database harmonization, or when the objective is to localize short-range, spatially constrained interactions. In these contexts, spatially aware methods tended to exhibit greater robustness and reproducibility, even when overall detection power was reduced. Thus, spatial awareness should be viewed as a complementary modeling component that enhances stability and interpretability.

Furthermore, an additional consideration highlighted by our analysis is the relationship between inference resolution and biological interpretability. In many ST studies, biological interpretation and experimental validation rely on pathway enrichment, literature support, and spatial colocalization of cell-type markers using techniques such as immunofluorescence, RNAscope, or spatial imaging. These validation strategies typically operate at the level of cell types, tissue regions, or signaling programs rather than at the level of individual cell–cell edges. In such settings, cell-type– or spot-cluster–level summaries can already provide robust and interpretable insights into tissue-scale communication patterns and are well aligned with common downstream validation workflows. Cell–level CCI methods, such as CellNEST, offer finer-grained hypotheses by inferring interactions between individual cells and are particularly valuable when the biological objective is to study local niche-specific signaling, fine-scale spatial heterogeneity, or interactions confined to restricted regions of interest. However, this increased resolution does not necessarily translate into improved performance under pathway-level evaluation criteria, nor does it imply universal superiority across datasets. Rather, our results indicate that the appropriate resolution for CCI inference should be matched to the specific biological question, data resolution, and validation strategy. Consequently, cell-type–level and cell-level CCI inference should be viewed as complementary approaches rather than competing paradigms.

Scalability emerged as another critical practical consideration. While several methods demonstrated strong biological relevance in large single-cell datasets, others encountered substantial computational bottlenecks when applied to high-throughput platforms such as Xenium or Stereo-seq. As ST technologies continue to advance toward higher resolution and larger tissue coverage, robustness to data sparsity, resolution heterogeneity, and computational scalability will become increasingly central to accurate and reproducible CCI inference.

Despite the comprehensive scope of this study, some limitations should be acknowledged. First, the absence of experimentally validated ground truth CCI in real ST datasets limits the ability to fully assess detection accuracy. Both the simulations with predefined cell-type-specific L-R expression patterns and the literature-confirmed pathway lists in real data application provide useful approximations; however, without confirmation from orthogonal experimental approaches, the evaluation remains constrained. Future studies employing validation techniques, such as multiplexed in situ hybridization, proximity ligation assays, or spatial proteomics will be essential for systematically and confidently benchmarking CCI methods. Second, differences in ligand–receptor database size, curation strategies, and inclusion of multimeric complexes or context-specific signaling inevitably contribute to performance variation across tools. Third, some methods were not originally designed for spot-level resolution data and may struggle with spatial resolution limitations or cell type mixtures inherent in platforms like Visium. Finally, although we evaluated nine recent developed tools, most introduced after 2023, ongoing updates and improvements to these frameworks may alter their relative performance, underscoring the need for periodic re-benchmarking.

## Conclusions

In summary, this study provides a systematic and quantitative evaluation of recent computational methods for inferring CCIs from ST data. Rather than identifying a single best-performing approach, our findings offer practical guidance for selecting appropriate tools across spatial resolutions and platforms and may inform the design of future benchmarking studies. As innovations in ST technologies and protocols progress, the comparative framework established in this study will be valuable for contextualizing emerging methods alongside existing benchmarks presented in published method papers.

## Methods

### Simulation design overview and expression-based embedding strategy

To enable controlled and interpretable evaluation of CCI inference methods, we employed an expression-based embedding strategy in which selected ligand and receptor genes are upregulated in spatially adjacent sender–receiver cell populations. This design allows explicit control over interaction strength, spatial localization, and cell-type specificity, while preserving realistic gene expression variability derived from scRNA-seq reference data. Our simulation goal was to assess whether CCI inference methods can recover cell-type–specific, spatially localized L-R signals under conditions where the location, magnitude, and spatial patterning of interactions are known. To this end, simulated L-R interactions were embedded within designated spatial regions by increasing ligand expression in sender cell types and receptor expression in neighboring receiver cell types. This design explicitly encodes both cell-type specificity and spatial dependence in the simulated data.

Expression-driven embedding has been widely adopted in prior CCI method development and benchmarking studies [12, 29], where expression magnitude serves as the primary observable signal of intercellular communication. Although this approach does not explicitly model L-R biophysics or signaling kinetics, spatial dependence is enforced by restricting signal enhancement to localized neighborhoods. As a result, simulated interactions occur only where sender and receiver cell types are spatially proximal, enabling systematic evaluation of method sensitivity to spatial localization and signal strength under controlled generative conditions.

### Ligand-receptor database harmonization and pair selection

To ensure fair and method-independent benchmarking, L-R pairs used in the simulation studies were selected from a harmonized L-R reference constructed by intersecting multiple widely used L-R databases from the nine evaluated methods, including CellChatDB, CellPhoneDB, CellTalkDB, FANTOM5, Ramilowski, and CellNESTDB. This harmonization strategy ensured that all evaluated methods operated on an identical L-R universe, preventing any method from benefiting from proprietary database expansions or differences in curation scope. As several benchmarking methods do not support multisubunit ligands or receptor complexes, we further restricted the harmonized L-R reference to single-chain L-R pairs that were compatible with all included tools. This compatibility filtering avoided excluding methods or altering their original modeling assumptions, and provided a conservative, method-agnostic solution for database harmonization

To ensure biological feasibility, in simulated studies, we retained only L-R pairs whose ligand and receptor genes were detected in the scRNA-seq reference dataset used for expression modeling and were expressed in the designated simulated sender and receiver cell types. From this filtered and biologically feasible L-R set, L-R pairs were randomly sampled for embedding in the simulation studies. Importantly, L-R selection was not based on known signaling pathways, literature priors, or functional categories, thereby avoiding bias toward specific molecular classes or interaction types. This random sampling strategy was designed to minimize the influence of prior biological knowledge on downstream performance, ensuring that observed differences among methods reflect their underlying modeling strategies rather than biases in L-R selection or database structure. In all simulation experiments, L-R pairs were restricted to this harmonized intersection-based reference, fully controlling for database heterogeneity during simulation benchmarking.

For real-data analyses, we adapted both each method’s native L-R database (native-DB) and the harmonized intersection-based reference (common-DB), wherever technically feasible. This dual analysis allows assessment of method robustness to database choice while preserving each tool’s original design assumptions. Throughout the manuscript, simulation results are therefore fully controlled for database effects, whereas real-data results explicitly evaluate the impact of L-R database heterogeneity.

### Simulation study with spot-level resolution spatial datasets

To evaluate cell–cell interaction inference methods at spot-level resolution, we generated 100 synthetic spot-level spatial transcriptomics (ST) datasets by combining real spatial coordinates adapted from a pancreatic cancer Visium experiment [36] with single-cell expression profiles resampled from a human pancreas scRNA-seq reference from Baron et al. (GSE84133) [31]. The reference dataset comprises 14 annotated cell types (alpha, beta, delta, gamma, epsilon, acinar, ductal, activated stellate, quiescent stellate, endothelial, macrophage, mast, T cells, and Schwann). The simulation design was described as follows.

#### Cell type selection and spot composition

For the simulations, we restricted to seven abundant cell types: beta, delta, ductal, macrophage, activated stellate, quiescent stellate, and endothelial. Rarer types such as epsilon, mast, T cells, and Schwann contributed negligibly when aggregated into spots and could yield unstable parameter estimates. Each simulated spot was assigned 12 cells, reflecting the typical capture size of 10–15 cells per spot in Visium ST data. The coordinate framework contained 782 spots, preserving the geometry and density of real tissue sections and providing a realistic spatial scaffold for simulation.

#### Resampling cell types and generating expression profiles

To construct cell-type mixtures, weresampled the selected cell types in proportion to their frequencies in the reference scRNA-seq dataset. Each spot was therefore represented as a heterogeneous mixture of cell types, with varying compositions across the tissue grid to mimic realistic heterogeneity. Gene expression were simulated using a negative binomial distribution, which captures the mean–variance relationship and over-dispersion characteristic observed in sequencing data. For each gene within each cell type, distribution parameters (mean and dispersion) were estimated from the scRNA-seq reference data, and new single-cell expression values were sampled from these fitted distributions. Genes with fewer than 10 total UMIs or detected in fewer than 10 single cells were set to zero to avoid unstable fits. To model skewed gene expression patterns, sampled values were ranked such that subsets of cells was assigned higher expression levels, while the remainder retained baseline values. Spot-level expression matrices were then generated by summing the simulated single-cell counts within each spot, and spot-specific cell-type compositions were recorded for downstream benchmarking.

#### Embedding spatial structure and simulation-defined truth CCIs

Spatial structure was introduced by defining a central region of approximately 130 spots as enriched for specific L–R interactions, while peripheral regions retained baseline expression levels. Ligands were upregulated in central spots and their cognate receptors in adjacent (neighboring) spots, enforcing a spatial configuration consistent with paracrine signaling. Simulation-defined truth interactions were defined as L–R pairs in which both ligand and receptor expression exceeded global quantile thresholds, selected at 25th, 50th, or 75th percentiles, to mimick realistic sparsity in spot-level ST signals. A true positive interaction was defined when both ligand and receptor were co-localized in spatially adjacent enriched spots, while all other pairs were considered negatives. This simulation framework provided synthetic spot-level ST datasets with reproducible simulation-defined truth interactions labels, while preserving realistic variation in cell-type composition, gene expression distributions, and spatial organization.

### Simulation study with single cell resolution spatial datasets

To benchmark cell–cell interaction inference methods at single-cell resolution, we generated 50 synthetic ST datasets also using the human pancreatic scRNA-seq reference from Baron et al. (GSE84133) [31]. These simulations combine bioogically realistic expression patterns with spatial organization that mimics in vivo tissue architecture.

#### Cell types and spatial layouts

The coordinate framework contained 3,593 single cells from 10 major pancreatic cell types: acinar, activated stellate, alpha, beta, delta, ductal, endothelial, gamma, macrophage, and quiescent stellate. Cell type abundances were preserved in proportion to the original reference dataset. Rare populations such as epsilon, mast, T cells, and Schwann cells were excluded to avoid unstable parameter estimation. To simulate spatial coordinates were generated using Voronoi tessellation [52, 53], which partitions space into compact regions surrounding seed points. This approach produces contiguous, type-specific clusters that better reflect tissue-like organization than random placement, and also creates variable inter-cluster distances, allowing systematic testing of whether methods can detect interactions between spatially proximal versus more distant cell-type clusters.

#### Simulating gene expression profiles

Gene expression profiles were simulated by fitting negative binomial distributions to the UMI counts of each gene within each cell type, thereby modeling the mean–variance relationship and over-dispersion characteristics of scRNA-seq data. Genes with extremely sparse expression (fewer than 10 total UMIs or expressed in fewer than 10 cells) were filtered out to avoid unreliable parameter estimates. Remaining genes were resampled from the fitted distributions. We selected the negative binomial modeling over the simpler Poisson model due to its superior fit to scRNA-seq count variability. All datasets were generated with a fixed random seed to ensure reproducibility, and outputs included a cell-by-gene expression matrix together with metadata on cell type and spatial coordinates.

#### Embedding simulation-defined truth ligand-receptor interactions

To introduce simulation-defined truth CCIs, we modulated L–R expression within spatially defined sender–receiver neighborhoods. In each replicate, ligands were upregulated in designated sender cells and their cognate receptors in neighboring receiver cells. To assess sensitivity to interaction strength, we applied multiplicative fold-changes of 5$\times$, 10$\times$, or 15$\times$ over baseline expression levels in the simulated dataset. These levels were chosen to evaluate whether methods could reliably detect weak, moderate, or strong interaction signals. Sender–receiver neighborhoods were defined using Euclidean k-nearest neighbors with k = 18, 36, or 60, corresponding to progressively larger local spatial neighborhoods. Simulation-defined truth positives were defined as L–R pairs with simultaneous ligand and receptor upregulation in adjacent sender–receiver cells, reflecting the biological requirement for co-expression of both partners. All other L-R pairs were treated as negatives. This framework therefore enabled systematic benchmarking of method sensitivity to interaction distance, signal strength, and cell-type specificity at single-cell resolution. By integrating both spatial and molecular simulation-defined truth, these synthetic single-cell ST datasets provide a robust testbed for evaluating the resolution, sensitivity, and specificity of computational CCI detection approaches.

To evaluate spatial specificity rather than long-range signaling per se, we further contrasted proximal and distal sender–receiver configurations. Proximal interactions correspond to spatially adjacent sender–receiver cell populations, where L-R signaling is biologically plausible through juxtacrine or paracrine mechanisms [54, 55]. Distal configurations were generated by intentionally separating sender and receiver cell types into non-adjacent spatial regions and were included as negative controls, not to model biologically realistic long-range signaling. This proximal–distal contrast therefore serves as a methodological stress test of spatial awareness, assessing whether CCI inference methods appropriately down-weight or eliminate predicted interactions when spatial proximity is disrupted.

### Statistical thresholds and evaluation protocol

In simulation benchmarking, method performance was evaluated across multiple statistical significance thresholds (*p* < 0.05, 0.10, and 0.20) to assess robustness and sensitivity under simulation-defined truth. These thresholds were applied uniformly to methods that employ statistical hypothesis testing when computing precision, recall, specificity, and F1 score. The inclusion of relaxed thresholds was intended solely for simulation studies to avoid scenarios in which certain methods return very few or no detected interactions under stringent cutoffs, which would preclude meaningful sensitivity assessment.

In all real ST data analyses, each method was evaluated following its recommended default criteria, with statistical-testing–based methods using conventional significance thresholds (*p* < 0.05) as specified in their original protocols, while the deep learning–based method was evaluated using model-derived confidence score and rank according to the original method specifications.

### Evaluation metrics

To quantitatively evaluate the performance of each cell-cell interaction inference method, we employed five evaluation metrics, encompassing both classification accuracy and reproducibility. The definition and interpretations of each metric are described below.

1. **Precision.** Precision measures the proportion of predicted interactions that are true positives:
  1 $$\begin{aligned} Precision = \frac{TP}{TP+FP} \end{aligned}$$
  Here, *TP* (true positives) are interactions correctly identified as present, while *FP* (false positives) are predicted interactions that are not supported by the ground truth in simulations or lack biological support in real datasets. A high precision value indicates that the method produces few false positives, reflecting high confidence in its predictions. In simulated datasets, true positives are defined as ground-truth interactions correctly detected between sender and receiver cell types. In real datasets, true positives are defined as literature-supported pathway-level interactions among known cell types.
2. **Recall.** Recall, also known as sensitivity or true positive rate, quantifies the proportion of ground-truth interactions that are successfully recovered
  2 $$\begin{aligned} Recall = \frac{TP}{TP+FN} \end{aligned}$$
  Here, *FN* (false negatives) are true interactions that the method failed to detect. A high recall suggests that the method is sensitive and able to detect a large proportion of true CCIs. However, high recall alone may come at the cost of more false positives.
3. **F1 score.** To balance the trade-off between precision and recall, we computed the F1 score, defined as the harmonic mean of the two:
  3 $$\begin{aligned} F1 = \frac{2\cdot Precision \cdot Recall}{Precision+Recall} \end{aligned}$$
  The F1 score ranges from 0 to 1, where a value of 1 indicates perfect precision and recall. This metric is especially useful when one wants to balance false positive and false negative rates, which is often critical in noisy single-cell and spatial transcriptomics datasets.
4. **Specificity.** Specificity, or the true negative rate, measures the ability of the method to correctly reject non-interactions:
  4 $$\begin{aligned} Specificity = \frac{TN}{TN+FP} \end{aligned}$$
  Here, *TN* (true negatives) are interaction pairs correctly identified as absent. In simulated datasets, TNs correspond to ligand–receptor pairs that do not meet expression or spatial proximity thresholds. In real datasets, they represent interactions not enriched or not supported by prior biological evidence. High specificity indicates effective suppression of spurious predictions, which is important when the majority of all possible interactions are negative (sparse setting).
5. **Jaccard Index.** The Jaccard index was used to assess the consistency of predicted interactions across different slices in the DLPFC datasets and the IPMN datasets within the same pathological grade. For each pair of slices $i$ and $j$, we defined
  $$\begin{aligned} J(i,j) = \frac{|P_i \cap P_j|}{|P_i \cup P_j|}, \end{aligned}$$
  where $P_i$ and $P_j$ denote the sets of predicted interactions in slices $i$ and $j$, respectively, and $|\cdot |$ indicates the cardinality (number of elements) of a set. The numerator $|P_i \cap P_j|$ is the number of interactions shared between the two slices, while the denominator $|P_i \cup P_j|$ is the total number of unique interactions across them.
  To obtain a global reproducibility score across all slices, we computed the average Jaccard index:
  $$\begin{aligned} \overline{J} = \frac{1}{\left( {\begin{array}{c}n\\ 2\end{array}}\right) } \sum _{1 \le i < j \le n} J(i,j), \end{aligned}$$
  where $n$ is the total number of slices and $$\left( {\begin{array}{c}n\\ 2\end{array}}\right)$$ is the number of unique slice pairs.

### Real data analyses

We curated nine spatial transcriptomics datasets spanning diverse tissue types, biological contexts, and experimental conditions to evaluate the performance of cell–cell interaction inference methods. These include three human pancreatic samples from intraductal papillary mucinous neoplasm (IPMN) patients [36], four human prefrontal cortex slices from the DLPFC dataset [37, 38], one mouse embryo dataset [32], and one human lung cancer dataset [51]. Detailed information for each dataset, including preprocessing steps and cell type annotations, is described below.

**IPMN patients** The IPMN dataset was obtained from a recent study profiling human pancreatic intraductal papillary mucinous neoplasms [36]. For primary analyses, we included three tissue sections: one low-grade IPMN sample (LG1: GSM7421780), one high-grade IPMN sample (HG1: GSM7421787), and one IPMN-associated pancreatic ductal adenocarcinoma (PDAC) sample (PDAC3: GSM7421792), as all evaluated methods were able to produce valid CCI results for these sections. Raw and the preprocessed Visium count matrices and spatial coordinates were downloaded from Gene Expression Omnibus (GEO) under accession GSE233254 [56], and matched scRNA-seq data were obtained from GEO under accession GSE233293 [57]. Cell type deconvolution was performed with RCTD as described in the original study. To simplify downstream analysis, fine-grained immune and stromal subtypes were collapsed into major lineages: for example, B-cell subclusters (activated, naive, memory) were aggregated into a single “B cell” category; NK-cell subclusters (CD16.NK, CD56.NK, NK.T) into “NK cell”; dendritic cell subtypes (DC1, DC2/3, pDC) into “DCs”; and multiple T-cell subtypes (CD4/CD8 effector, naive, memory, Treg, MAIT, etc.) into “T cell”. Similarly, endothelial, epithelial, fibroblast, myeloid, mast, and plasma cell identities were retained at higher levels. In total, the original set of more than 20 fine-grained subtypes was consolidated into 11 major cell types. Because RCTD outputs proportional cell type assignments per spot, we further annotated each spot by its dominant cell type (i.e., the category with the highest proportion) when benchmarking methods that require single-cell inputs. For robustness and cross-sample consistency analyses, we additionally included three low-grade IPMN samples (LG1–LG3), three high-grade IPMN samples (HG1–HG3), and three IPMN-associated PDAC samples (PDAC1–PDAC3).

**DLFPC** The DLPFC dataset comprises spatial transcriptomics profiles of human prefrontal cortex tissue generated with Visium technology and was originally published by researchers from the Lieber Institute for Brain Development (ILIBD) [37]. We included four control slices: 151507, 151508, 151509, and 151510, which were come from the same donor and were previously analyzed in studies on spatial domain detection and cortical layer structure. Preprocessed data were downloaded from the IRIS portal [38], which applies the IRIS pipeline to integrate Visium profiles with matched single-nucleus RNA-seq references. IRIS provides spot-level cell type proportions for excitatory neurons, inhibitory neurons, astrocytes, oligodendrocytes, OPCs, microglia, and endothelial cells, along with cortical layer annotations. We adopted these annotations as they have been validated in recent studies. For benchmarking methods that require discrete single-cell inputs, each spot was further annotated by its dominant cell type.

**Mouse embryo** The mouse embryo dataset [32] was obtained from the MOSTA (Mouse Spatial Transcriptomic Atlas) project, which profiled spatial gene expression across developmental stages using Stereo-seq technology. We included the sample E10.5_E2S1, corresponding to embryonic day 10.5. This dataset represents developing mouse tissue and provides high-resolution spatial information in a developmental context, which contains 8,494 cells across 18 annotated cell types. Processed spatial transcriptomics including count data along with cell type annotations were provided and downloaded from the MOSTA data portal by Spatial Transcript Omics DataBase (STOmics DB) [58]. As the dataset was already provided with curated cell type labels derived from clustering and marker gene analysis, we used these annotations without modification.

**Human lung cancer** The human lung cancer dataset [51] was generated using the Xenium Prime 5 K platform and released by 10x Genomics. It profiles formalin-fixed paraffin-embedded (FFPE) human lung cancer tissue at subcellular resolution, with over 278,000 cells and expression data for 5,001 genes. The tissue was classified as invasive acinar adenocarcinoma (stage IB, T2a N0 MX), a histological subtype of non-small cell lung cancer (NSCLC). We processed the raw Xenium output using Seurat. Counts were normalized with SCTransform, followed by dimensionality reduction with PCA (30 principal components) and UMAP. We then constructed a shared-nearest-neighbor graph using the top 30 PCs and applied FindNeighbors and FindClusters with a resolution of 0.3 to identify transcriptionally distinct clusters, where marker genes for each cluster were identified with FindAllMarkers (positive markers only; threshold avg_log2FC> 1). Cell type annotations were assigned by comparing cluster-specific marker genes to the reference marker lists provided by 10x Genomics. In total, we identified 16 cell types, including epithelial (alveolar AT1, AT2, airway/ciliated/secretory, mixed AT1/AT2), endothelial/capillary, stromal (fibroblast/myofibroblast, fibromyocyte/smooth-muscle–like), and immune populations (T cells, B cells, plasma cells, monocytes/macrophages, mast cells), as well as a neuroendocrine subset. One additional cluster was labeled as unannotated because its marker genes could not be confidently assigned to a known lineage, likely reflecting stressed or mixed states. Because the full dataset exceeded the scalability of some benchmarking methods (e.g., SpaTalk), we restricted comparative analyses to a representative tumor-containing region, which contains 44,704 cells and was identified using the accompanying fluorescence image, ensuring biologically meaningful tumor environment while maintaining computational feasibility.

**Pathway enrichment** We conducted KEGG pathway enrichment [59, 60] separately for each of the nine datasets and for each method. For a given dataset–method combination, the input gene set was the union of ligands and receptors from L-R pairs meeting the method’s significance threshold; the background set comprised all ligand and receptor genes that the method evaluated on that dataset. Gene identifiers were standardized to the appropriate human or mouse reference before testing. *P*-values were adjusted using the Benjamini–Hochberg procedure, and pathways with adjusted *p*-value smaller than 0.05 were retained. Analyses and any comparisons were performed within datasets only. KEGG pathway definitions were used to summarize L-R pathway enrichment results because they capture curated signaling cascades and involve less term redundancy than ontology-style annotations, which facilitates interpretation in this study.

**Literature-curated pathway** To contextualize enrichment results, we performed separate, structured literature searches for each dataset’s biological setting (IPMN low-grade, IPMN high-grade, IPMN-associated PDAC; four DLPFC brain datasets; NSCLC lung; mouse embryo E10.5). Searches combined pathway names with precise tissue and stage keywords (e.g., “WNT signaling” AND “mouse embryo” AND “E10.5”, “TGF-$\beta$ pathway” AND “NSCLC”, “SEMA3 signaling” AND “IPMN high grade”). We considered a pathway supported when peer-reviewed sources in the matching context reported involvement of that pathway (e.g., expression patterns, functional or phenotypic association, or histology evidence); not supported when sources indicated the pathway is implausible or contradicted for that context; and unknown when no relevant evidence was located.

### Parameter settings for inference methods in this study

We evaluated the performance of eight cell–cell interaction inference methods for spatial transcriptomics, all of which are primarily based on statistical frameworks. For each method, we used default or recommended parameter settings provided by the authors and official documentation.

1. **CellPhoneDB v3** We followed the instructions provided on the CellPhoneDB website: https://cellphonedb.readthedocs.io/en/latest/index.html. Since CellPhoneDB treats each spot as a single cell type in spot-level spatial transcriptomics analysis, we assigned to each spot its dominant cell type annotation. We performed statistical inference using CellPhoneDB v3 [15], which first introduced support for spatial data. Significant cell type specific interactions were selected based on a *p*-value threshold of 0.05, following the official tutorial. Although the inference was performed using v3, we utilized the updated ligand–receptor reference database from CellPhoneDB v5 [16] to ensure broader and more current coverage of signaling interactions.
2. **CellChat v2** We followed the tutorial and guidelines provided in the CellChat v2 GitHub repository: https://github.com/jinworks/CellChat. As CellChat v2 treats each spot as a single cell type in spot-level spatial transcriptomics analysis, we annotated each spot using its dominant cell type. Significant cell type specific ligand–receptor interactions were identified using a *p*-value threshold of 0.05. For dataset-specific parameter tuning, we referred to the recommendations in the FAQ for spatial transcriptomics analysis.
3. **SpaTalk** We followed the guidelines provided in the SpaTalk GitHub repository: https://github.com/ZJUFanLab/SpaTalk. For spot-level spatial transcriptomics data, SpaTalk internally reconstructs pseudo single-cell resolution based on its spatial graph and deconvolution framework. Significant cell type specific ligand–receptor interactions were identified using a *p*-value threshold of 0.05.
4. **SpatialDM** We followed the tutorial and parameter settings available at the SpatialDM website: https://spatialdm.readthedocs.io/en/latest/. According to the documentation, we used a permutation-based approach for datasets with fewer than 10,000 cells or spots, and switched to a z-score–based approach for larger datasets to reduce computational burden. Significant ligand–receptor interactions were identified using a *p*-value threshold of 0.05.
5. **COMMOT** We followed the official tutorial and parameter recommendations at the COMMOT website: https://commot.readthedocs.io/en/latest/index.html. Significant cell type specific ligand–receptor interactions were identified using a *p*-value threshold of 0.05.
6. **SCOTIA** We followed the guidelines and parameter settings provided in the SCOTIA GitHub repository: https://github.com/Caochris/SCOTIA. The ligand–receptor interactions were assessed using the reference database FANTOM5 provided in the original publication [18]. As SCOTIA treats each spot as a single cell type in spot-level spatial transcriptomics analysis, we annotated each spot using its dominant cell type. Significant cell-type-specific ligand–receptor interactions were identified using a *p*-value threshold of 0.05.
7. **NicheDE** We followed the tutorial provided on the NicheDE website: https://kaishumason.github.io/NicheDE/. The ligand–receptor list used for the analysis was followed from the tutorial data(“ramilowski_ligand_receptor_list”), which is based on the curated database from Ramilowski et al [61]. Significant cell-type-specific ligand–receptor interactions were identified using a *p*-value threshold of 0.05.
8. **SpaCCI** We followed the tutorial provided on the SpaCCI website: https://litingku.github.io/SpaCCI/. Ligand–receptor interactions were evaluated using the CellChat reference database, which provides curated signaling pairs. Significant cell-type-specific ligand–receptor interactions were identified using a *p*-value threshold of 0.05. SpaCCI reports interaction strength values as localized interaction probabilities that jointly incorporate ligand and receptor expression, local cell-type abundance, and spatial constraints. As a result of this probabilistic formulation, interaction scores are bounded and typically small in magnitude, and should be interpreted relative within a dataset rather than as unnormalized effect sizes. These values are used consistently throughout SpaCCI analyses for visualization of spatially localized interactions.
9. **CellNEST** We followed the guidelines and parameter settings provided in the CellNEST GitHub repository: https://github.com/schwartzlab-methods/CellNEST. CellNEST uses its own ligand–receptor database, which integrates interactions curated from CellChat and NicheNet, as described in the original publication. Unlike the other methods evaluated in this study, CellNEST infers interactions at the individual cell–cell level using a deep learning framework by inferring interactions ranked by model-derived confidence scores. Following the authors’ recommendations, we defined significant interactions as those within the top 20% of ranked interaction scores. For all analyses, models were trained using the default settings. Although CellNEST provides built-in visualization functions, we were unable to generate these visualizations due to known implementation issues reported in the public GitHub repository. Similar issues have been reported by other users, and this limitation does not affect the underlying interaction inference results used in our benchmarking.

### Computational efficiency and empirical scalability assessment on real data

We profiled wall-clock runtime and peak resident memory for each CCI inference method on three spatial transcriptomics platforms that span distinct scales and resolutions. For 10x Visium, we analyzed three IPMN sections (LG1/HG1/PDAC3: $\sim$982 spots each; $\sim$21,000 genes) and four DLPFC slices (151507/151508/151509/151510: $\sim$4,100 spots each; $\sim$33,500 genes). For Xenium 5 K (FFPE lung cancer; 44,704 cells; 5,001 genes) and MOSTA Stereo-seq (E10.5 embryo; 8,494 cells; 22,385 genes), we used representative large-scale datasets at single-cell or ultra-high-resolution granularity.

All runs were executed on a High-Performance Computing (HPC) cluster managed using IBS’s LSF (Load Sharing Facility) software. To maintain comparability while ensuring feasibility, we fixed the CPU allocation per job (28 cores where supported) and used platform/sample-specific memory requests that reflect typical workload size: 64 GB for IPMN datasets, 100 GB for DLPFC datasets, and higher method-dependent limits (typically 128–350 GB) for Xenium Prime 5 K and Stereo-seq. More memory-intensive tools (e.g., SpaTalk, COMMOT, SCOTIA) were assigned the upper range to avoid out-of-memory termination. Reported peak memory values correspond to the observed maximum resident memory recorded by LSF (Max Memory), rather than requested quotas. Runtime was obtained from scheduler logs, measured from job start to completion of the method’s CCI inference step. Where methods exposed internal parallelization, thread counts were aligned with the allocated cores. GPU acceleration was not used for these methods to avoid hardware-specific speedups.

As the spot-level Visium cohort contains multiple samples of different sizes, we computed scale-normalized efficiency metrics, average time per spot/cell and average time per gene, to enable cross-dataset comparisons within this platform. For Xenium Prime 5 K and Stereo-seq, each represented by a single large dataset, we report absolute resource profiles (runtime versus peak memory) that characterize efficiency at realistic single-dataset scales. Jobs that exceeded wall-time (the maximum allowed elapsed time for a job set in the scheduler) or terminated due to out-of-memory were recorded as failures and excluded from aggregate summaries of observed time and memory. All methods were run on identical inputs (expression matrices and annotations) and under the parameter choices described in other subsection in Methods to isolate computational differences from upstream preprocessing effects. This approach provides an empirical view of scalability across representative spatial data regimes, combining per dataset efficiency at single-cell/ultra-high-resolution scales (Xenium Prime 5 K, Stereo-seq) with scale-normalized comparisons across multiple spot-level Visium samples, while controlling for hardware and execution policies.

CellNEST differs from the other eight evaluated methods in its execution requirements. According to the official CellNEST implementation and documentation, GPU acceleration is required for model training and inference, and the method is distributed as a Singularity container optimized for GPU-enabled environments. We therefore executed CellNEST on GPU nodes following the authors’ recommended workflow, while all other methods were run in CPU-only environments. Runtime and memory usage for CellNEST were recorded from the same HPC scheduler logs as other methods but should be interpreted as reflecting the computational characteristics of a deep learning–based, GPU-dependent approach rather than being directly comparable to CPU-based statistical methods.

## Supplementary Information

Additional file 1: Figs. S1–S40. A single file containing all supplementary figures referenced in the manuscript. Captions and legends for each supplementary figure are included within the file.

Additional file 2: Tables S1–S4. An Excel file containing curated pathway lists with supporting literature references for the IPMN, DLPFC, MOSTA mouse embryo, and Xenium lung cancer datasets used in the benchmarking analyses. Table S1. Curated pathways associated with IPMN and supporting literature references. Table S2. Curated pathways associated with DLPFC spatial transcriptomics datasets and literature references. Table S3. Curated developmental pathways identified in the MOSTA mouse embryo dataset with literature sources. Table S4. Curated pathways associated with human lung cancer Xenium dataset with literature references.

## Data Availability

The raw spatial transcriptomics datasets analyzed in this study are publicly available from the following sources: the intraductal papillary mucinous neoplasm (IPMN) dataset generated using 10x Visium technology (GEO accession GSE233254) [56]; the dorsolateral prefrontal cortex (DLPFC) dataset generated using 10x Visium technology [62]; the mouse embryo dataset from the MOSTA (Mouse Spatial Transcriptomic Atlas) project at embryonic day E10.5 generated using Stereo-seq technology [58]; and the human lung cancer dataset generated using the Xenium Prime 5 K platform for FFPE tissue [51]. All processed spatial transcriptomics data with cell type annotations and the code used for data preprocessing, simulated data generation, and benchmarking analyses are publicly available on GitHub [63] and Zenodo [64] under an MIT license.
