# Supplementary material for: Benchmarking tools for deciphering cellular crosstalk in spatially-resolved transcriptomics
Source: Genome Biol. 2026 Apr 8;27:163. doi: 10.1186/s13059-026-04063-5 (PMC13174004; doi:10.1186/s13059-026-04063-5)
Supplement: Supplementary file 1 — Additional file 1: Figs. S1–S40. A single file containing all supplementary figures referenced in the manuscript. Captions and legends for each supplementary figure are included within the file. [file 13059_2026_4063_MOESM1_ESM.pdf]

Supplementary Figures

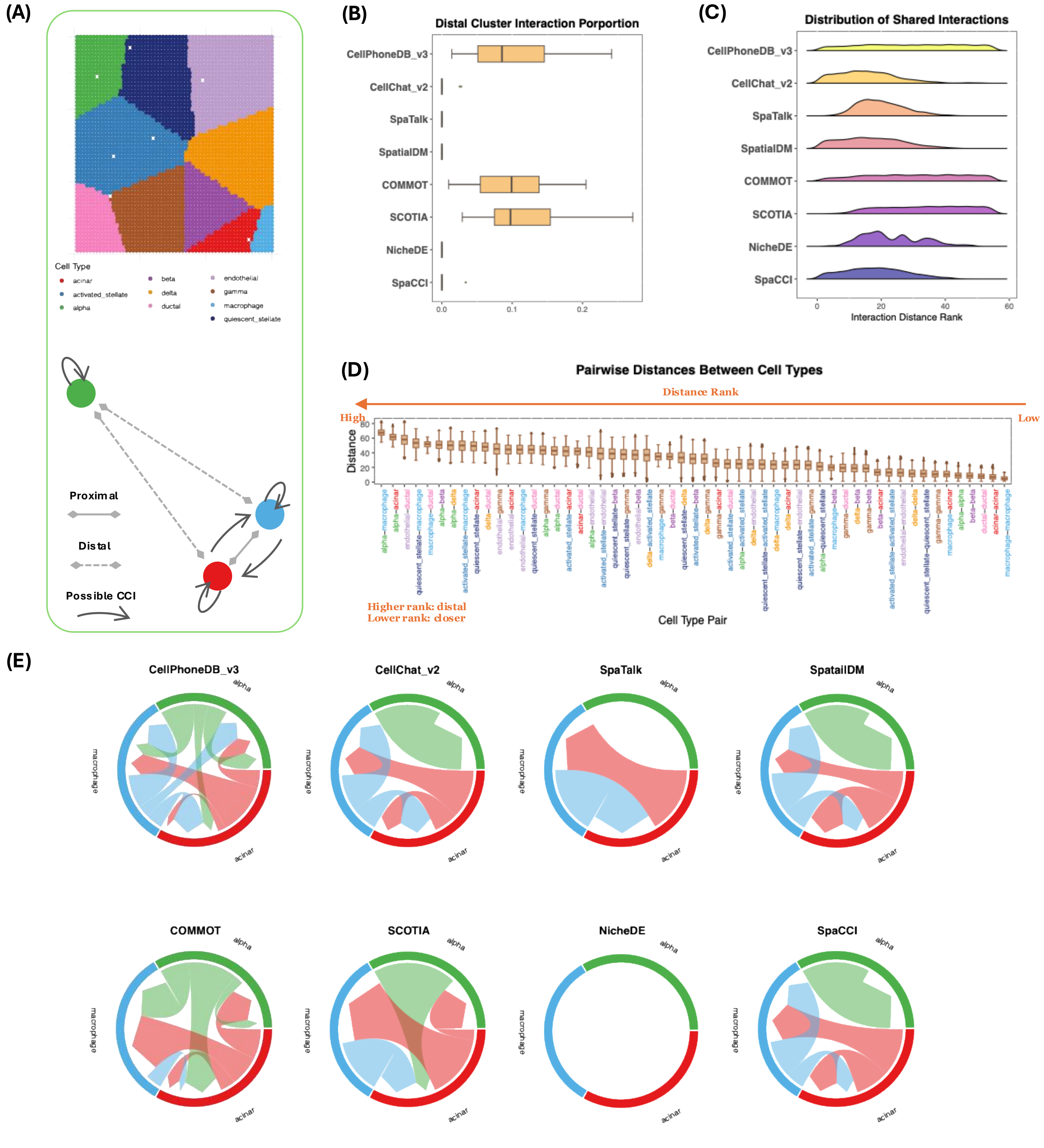

Fig. S1 | Evaluation of interaction distances in well-clustered simulated single-cell spatial transcriptomics data. (A) Visualization of the well-clustered simulated spatial transcriptomics dataset, where spatial relationships between cell types—proximal or distal—are clearly distinguishable. (B) Boxplot showing the distribution of interactions involving distal cell type clusters as detected by each CCI tool. (C) Ridge plots showing the distribution of inferred interaction distance ranks for each tool. On the x-axis, lower ranks represent proximal interactions between cell types, while higher ranks indicate distal interactions. (D) Boxplot illustrating how interaction distance ranks were defined in the analysis. Lower ranks correspond to interactions between nearby cell type clusters; higher ranks represent more spatially distant interactions. (E) Chord diagrams visualizing detected interactions in one simulated dataset, highlighting each tool's ability to capture interactions between proximal or distal cell type clusters.

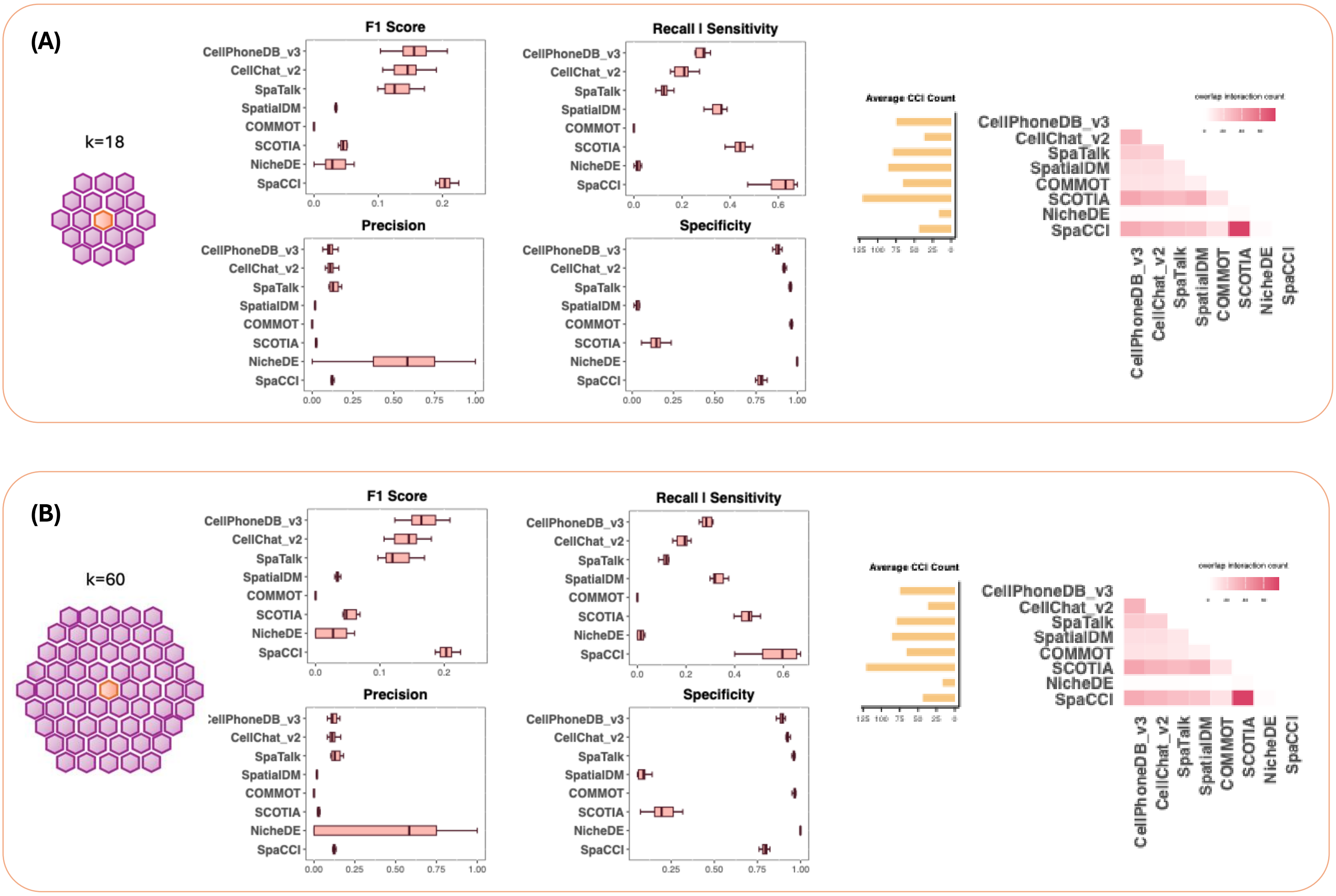

Fig. S2 | Evaluation of cell-cell interaction in simulated single-cell spatial transcriptomics data. (A) Performance under a simulation scenario with a spatial interaction range of k = 18, where interacting cell pairs exhibit a 10-fold increase in ligand-receptor expression, serving as the ground truth. Left: boxplots showing the distribution of F1 score, recall (sensitivity), precision, and specificity for each method. Right: bar plot showing the average number of detected interactions and a heatmap of overlap counts between methods. (B) Performance under a simulation scenario with a spatial interaction range of k = 60, where interacting cell pairs exhibit a 10-fold increase in ligand-receptor expression, serving as the ground truth.

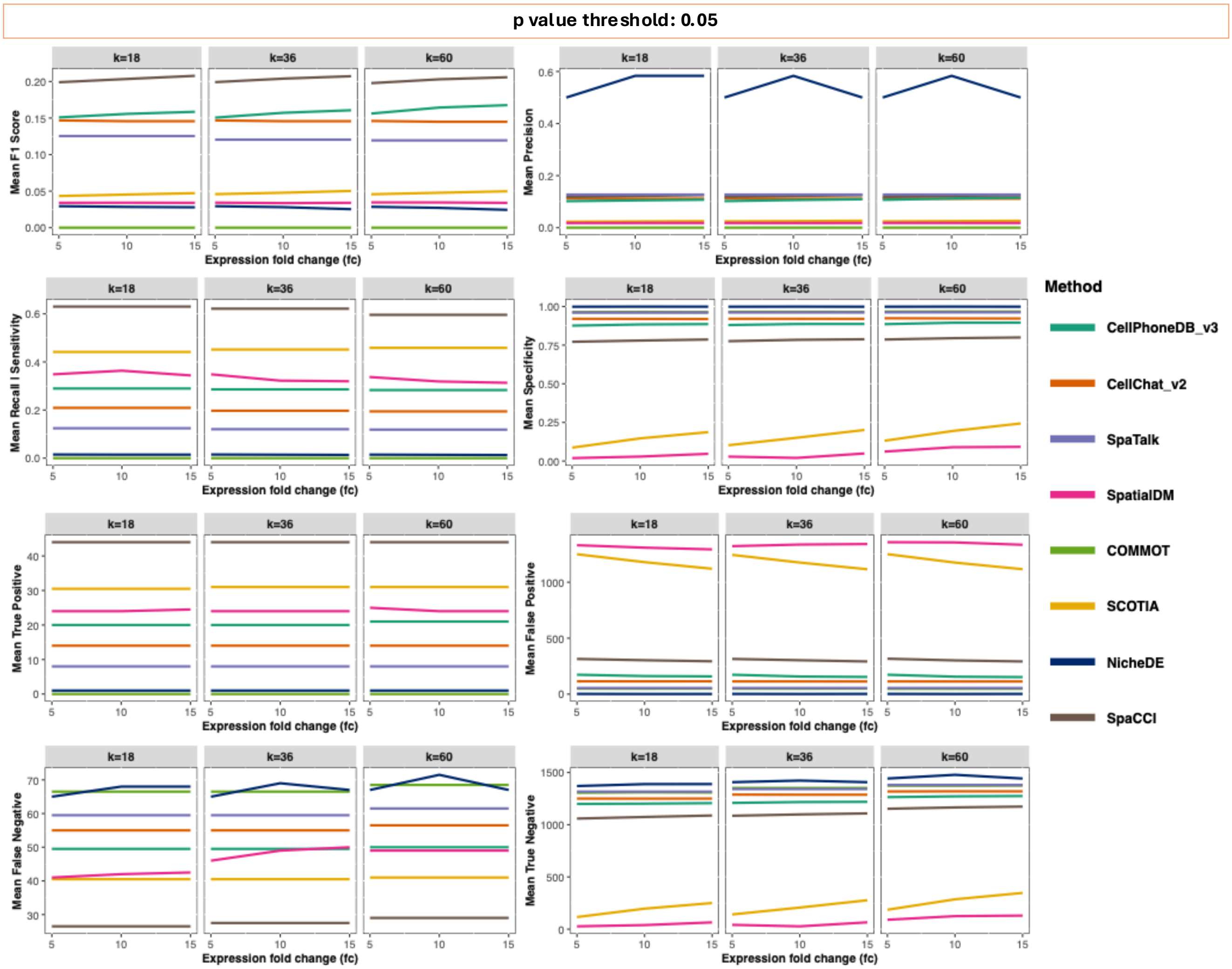

Fig. S3 | Evaluation of cell-cell interaction in simulated single-cell spatial transcriptomics data. Performance metrics, including mean F1 score, precision, recall (sensitivity), specificity, true positives, false positives, false negatives, and true negatives, are shown across all simulation scenarios. Simulations were conducted under varying spatial interaction ranges (k = 18, 36, and 60) and ligand–receptor expression fold changes (5×, 10×, and 15×), which define the ground truth interactions. A significance threshold of $p < 0.05$ was applied to determine detected interactions.

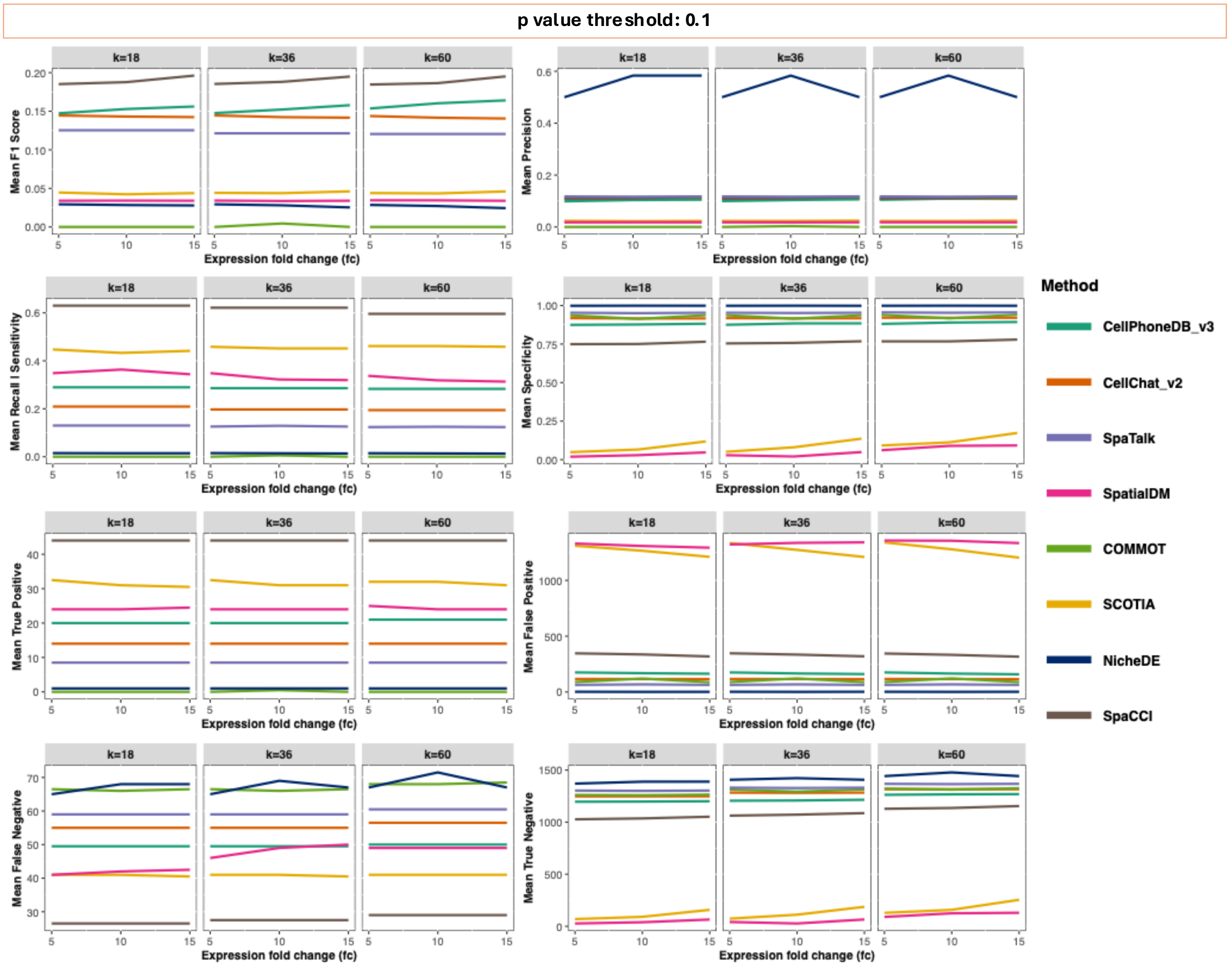

Fig. S4 | Evaluation of cell-cell interaction in simulated single-cell spatial transcriptomics data. Performance metrics, including mean F1 score, precision, recall (sensitivity), specificity, true positives, false positives, false negatives, and true negatives, are shown across all simulation scenarios. Simulations were conducted under varying spatial interaction ranges (k = 18, 36, and 60) and ligand–receptor expression fold changes (5×, 10×, and 15×), which define the ground truth interactions. A significance threshold of $p < 0.1$ was applied to determine detected interactions.

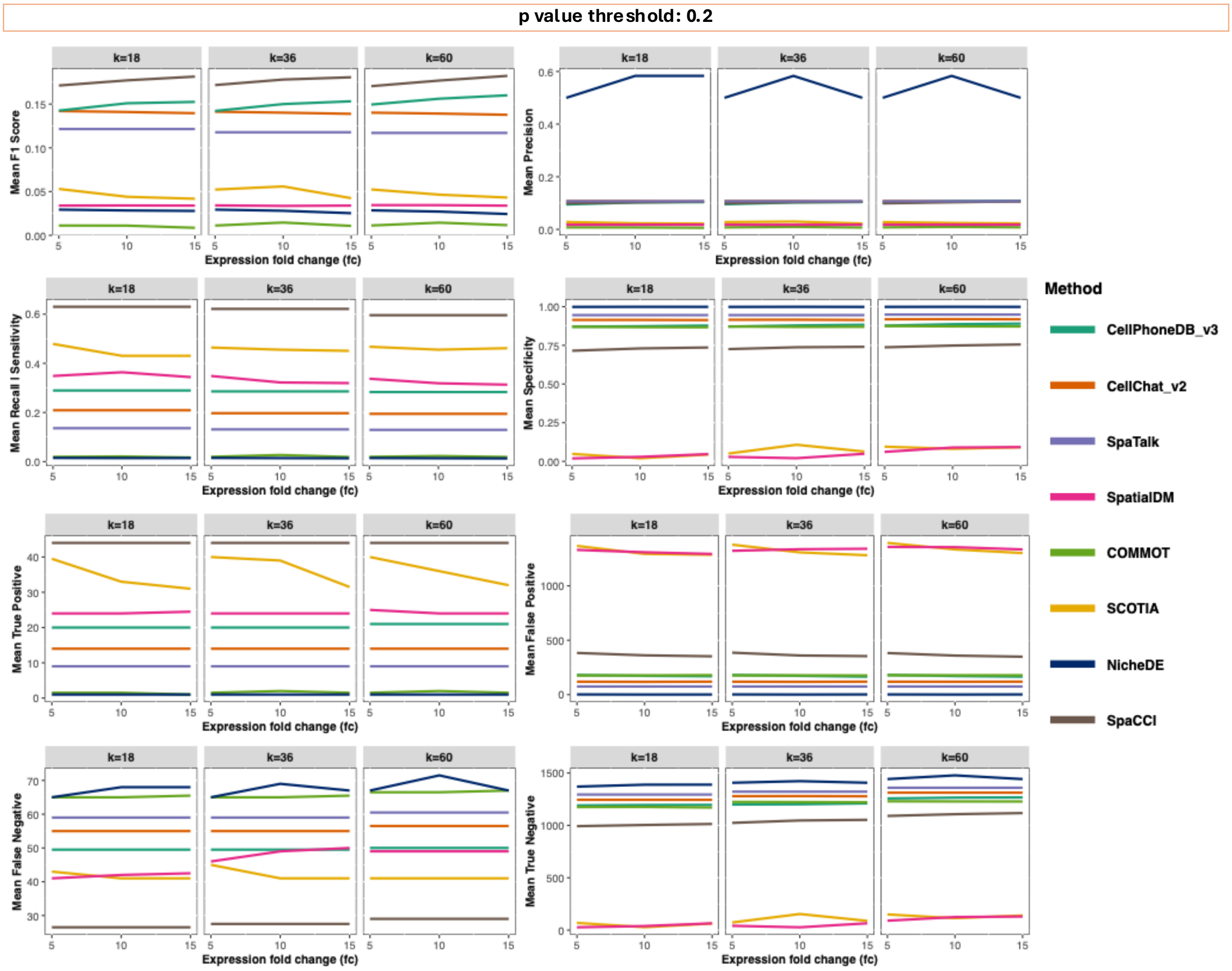

Fig. S5 | Evaluation of cell-cell interaction in simulated single-cell spatial transcriptomics data. Performance metrics, including mean F1 score, precision, recall (sensitivity), specificity, true positives, false positives, false negatives, and true negatives, are shown across all simulation scenarios. Simulations were conducted under varying spatial interaction ranges (k = 18, 36, and 60) and ligand–receptor expression fold changes (5×, 10×, and 15×), which define the ground truth interactions. A significance threshold of $p < 0.2$ was applied to determine detected interactions.

(A)

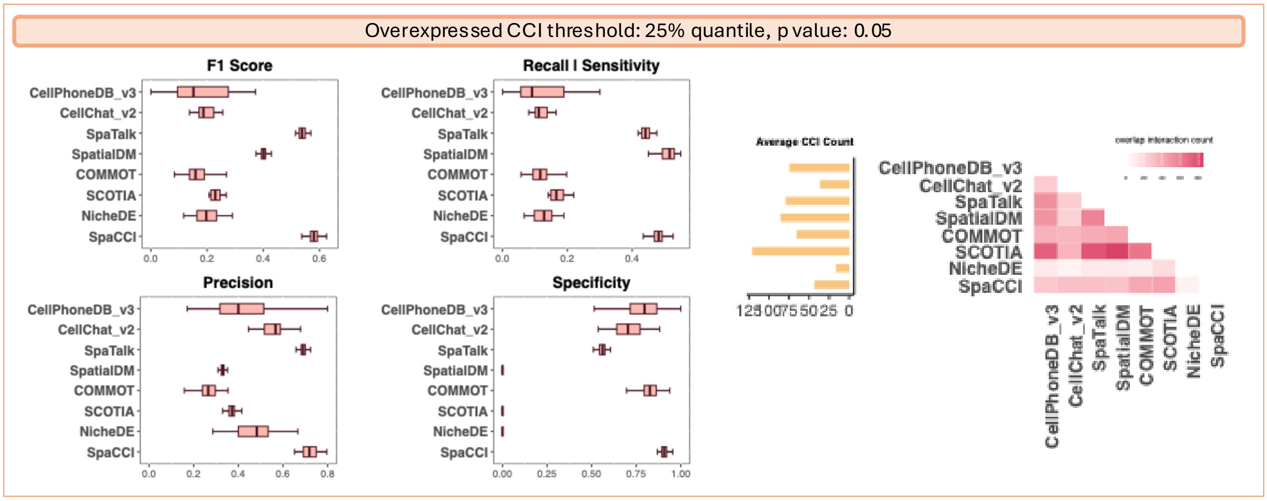

(B)

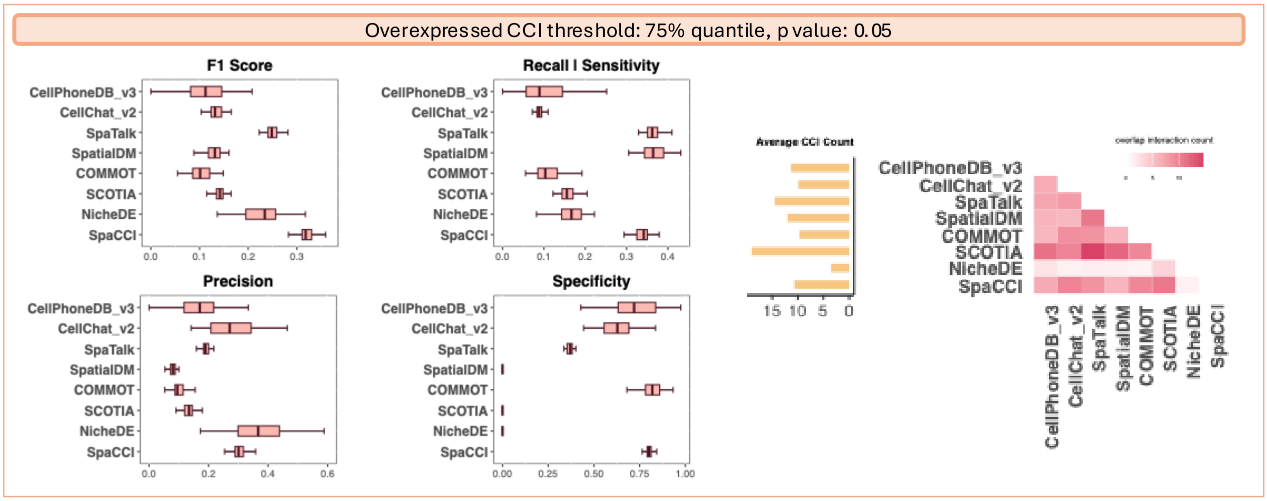

Fig. S6 | Evaluation of cell-cell interaction in simulated spot-level spatial transcriptomics data. (A) Performance under a simulation scenario where ground truth interactions are defined as ligand-receptor pairs overexpressed above the median (25th percentile) relative to global expression, with a significance threshold of $p < 0.05$ applied during analysis. Left: boxplots of F1 score, recall, precision, and specificity. Right: average number of detected interactions and overlap counts between methods. (B) Performance under a simulation scenario where ground truth interactions are defined as ligand-receptor pairs overexpressed above the median (75th percentile) relative to global expression, with a significance threshold of $p < 0.05$ applied during analysis.

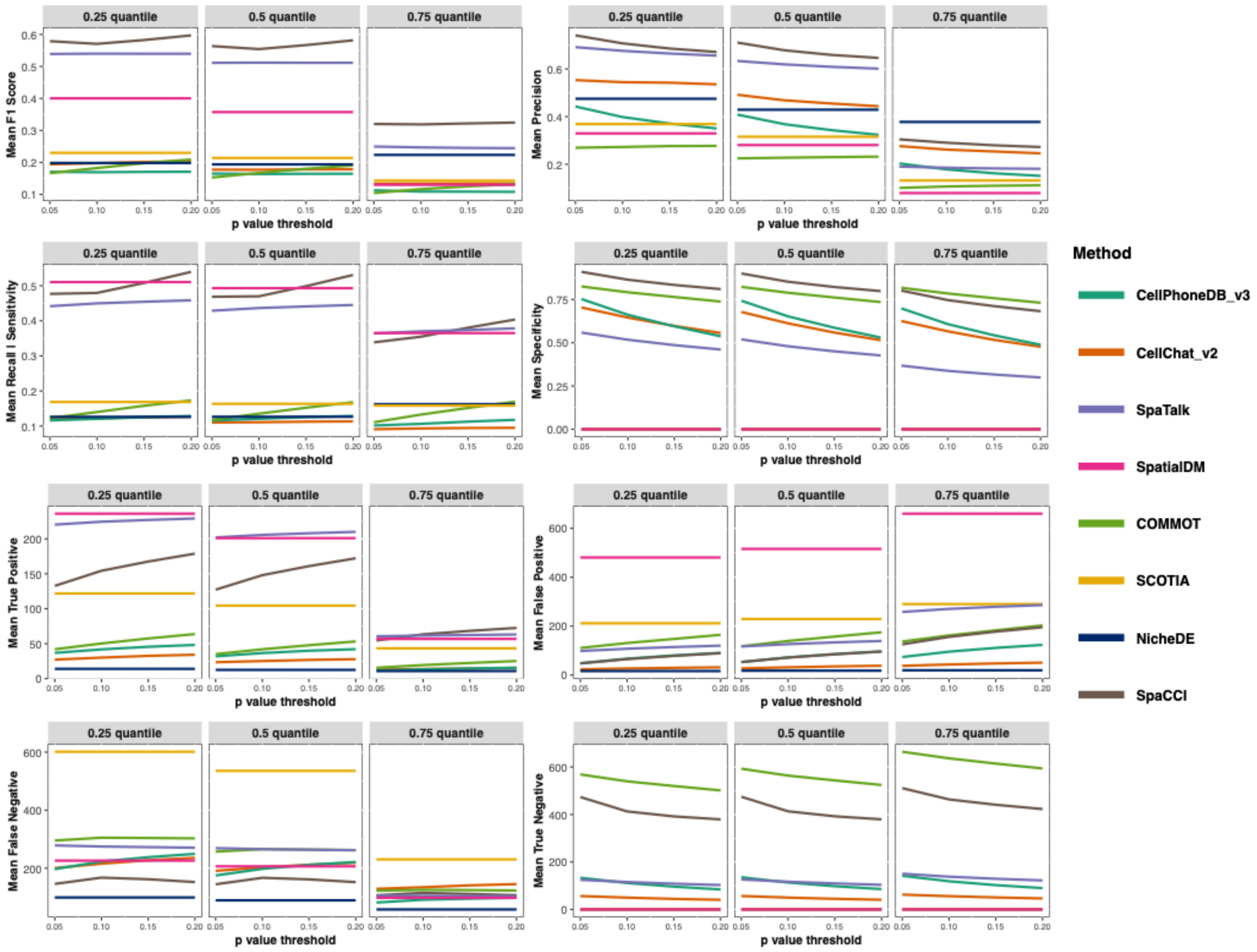

Fig. S7 | Evaluation of cell-cell interaction in simulated spot-level spatial transcriptomics data. Performance metrics—including mean F1 score, precision, recall (sensitivity), specificity, true positives, false positives, false negatives, and true negatives—are shown across all simulation scenarios. Ground truth interactions were defined based on ligand–receptor pairs overexpressed above specific quantile thresholds (25th, 50th, and 75th percentiles), with detection evaluated under varying significance thresholds (p = 0.05, 0.1, and 0.2).

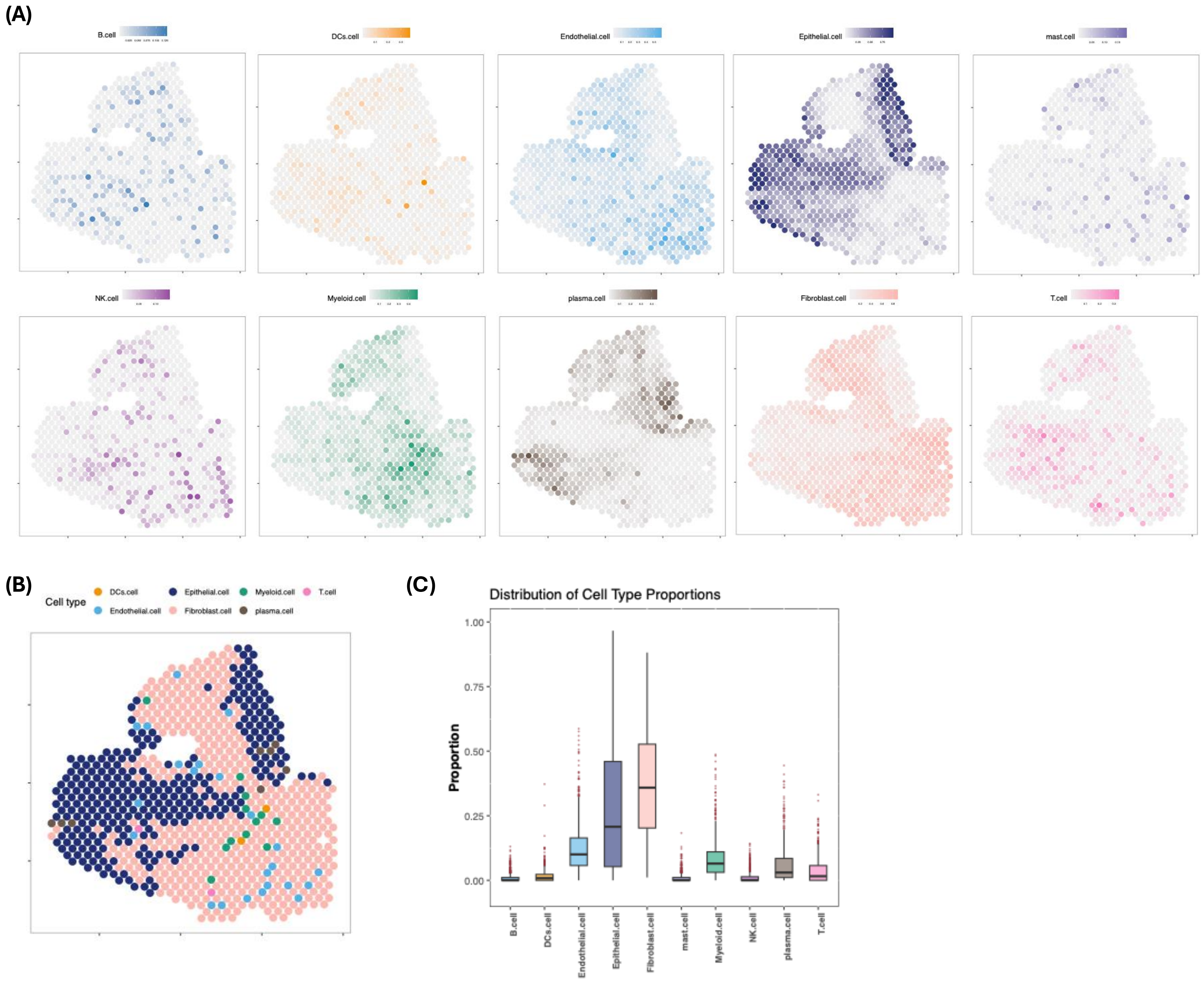

Fig. S8 | Cell type composition in the 10x Visium IPMN dataset (sample LG1: Low Grade IPMN). (A) Spatial distribution of cell type proportions across the tissue slide, showing estimated abundance for each major cell type per spot. (B) Spot-wise cell type annotation for methods that assume a single dominant cell type per spot. Each spot is labeled with the most abundant cell type. (C) Boxplot showing the distribution of cell type proportions across all spots in the sample.

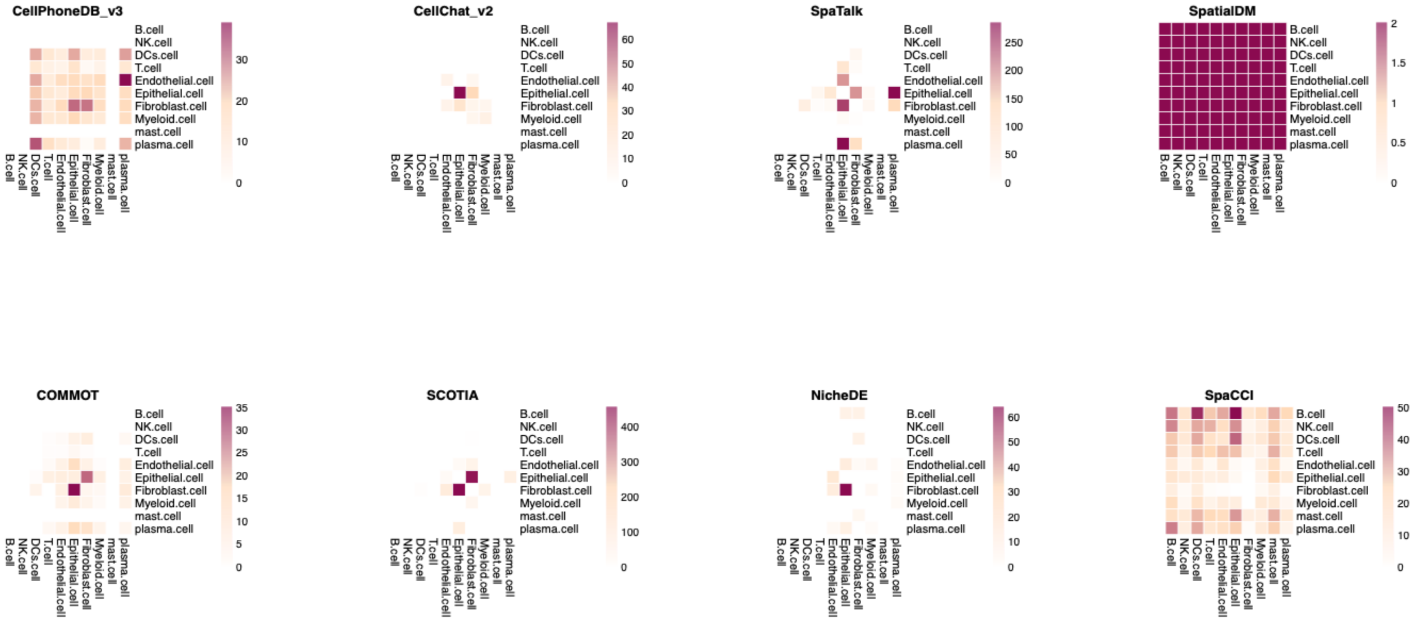

Fig. S9 | Cell-cell interaction inference in the 10x Visium IPMN dataset (sample LG1: Low Grade IPMN). Heatmaps showing the number of inferred ligand–receptor interactions between cell type pairs, as detected by each of the eight computational tools. Rows represent sender cell types, and columns represent receiver cell types. The color intensity reflects the number of detected interactions.

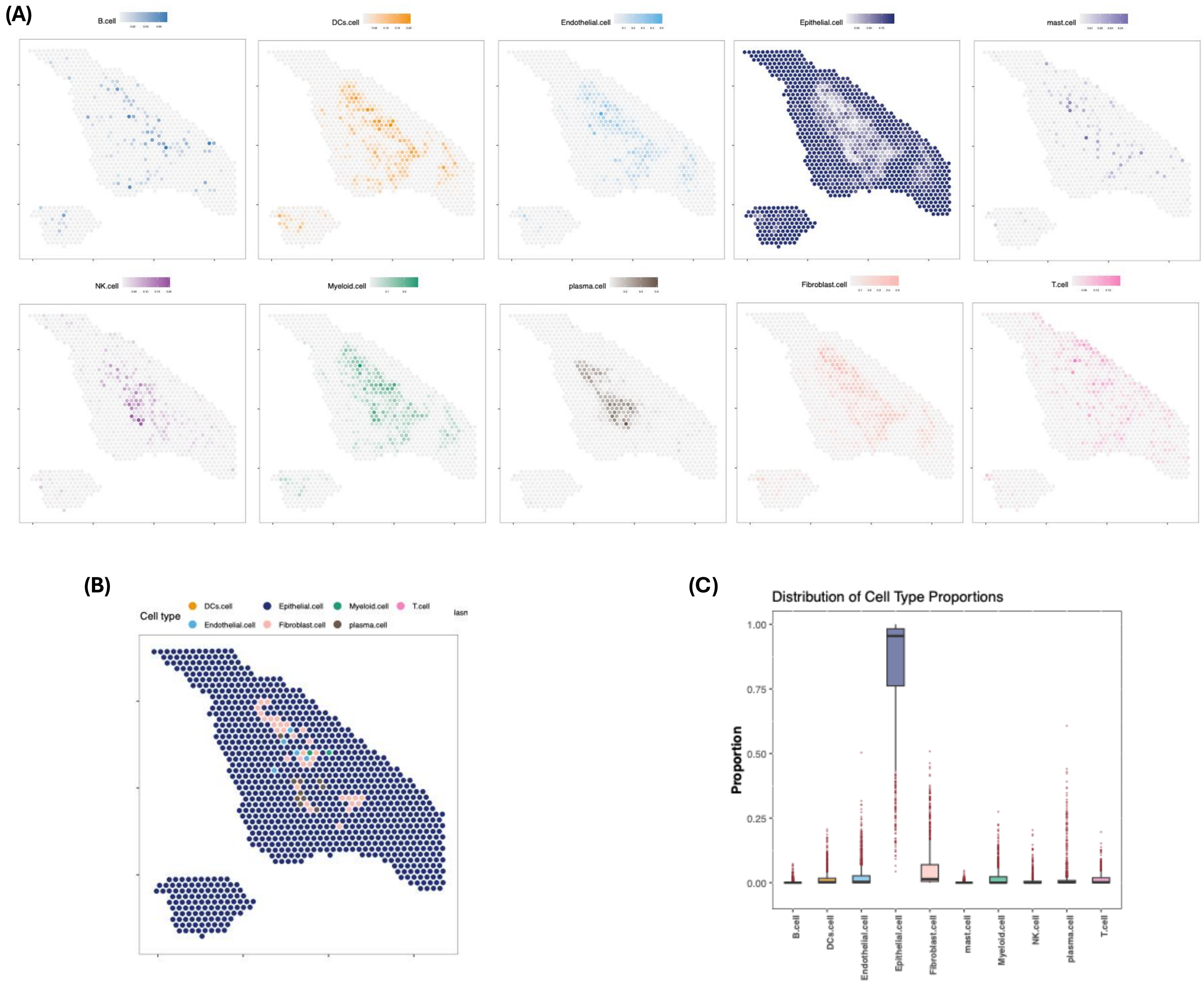

Fig. S10 | Cell type composition in the 10x Visium IPMN dataset (sample HG1: High Grade IPMN). (A) Spatial distribution of cell type proportions across the tissue slide, showing estimated abundance for each major cell type per spot. (B) Spot-wise cell type annotation for methods that assume a single dominant cell type per spot. Each spot is labeled with the most abundant cell type. (C) Boxplot showing the distribution of cell type proportions across all spots in the sample.

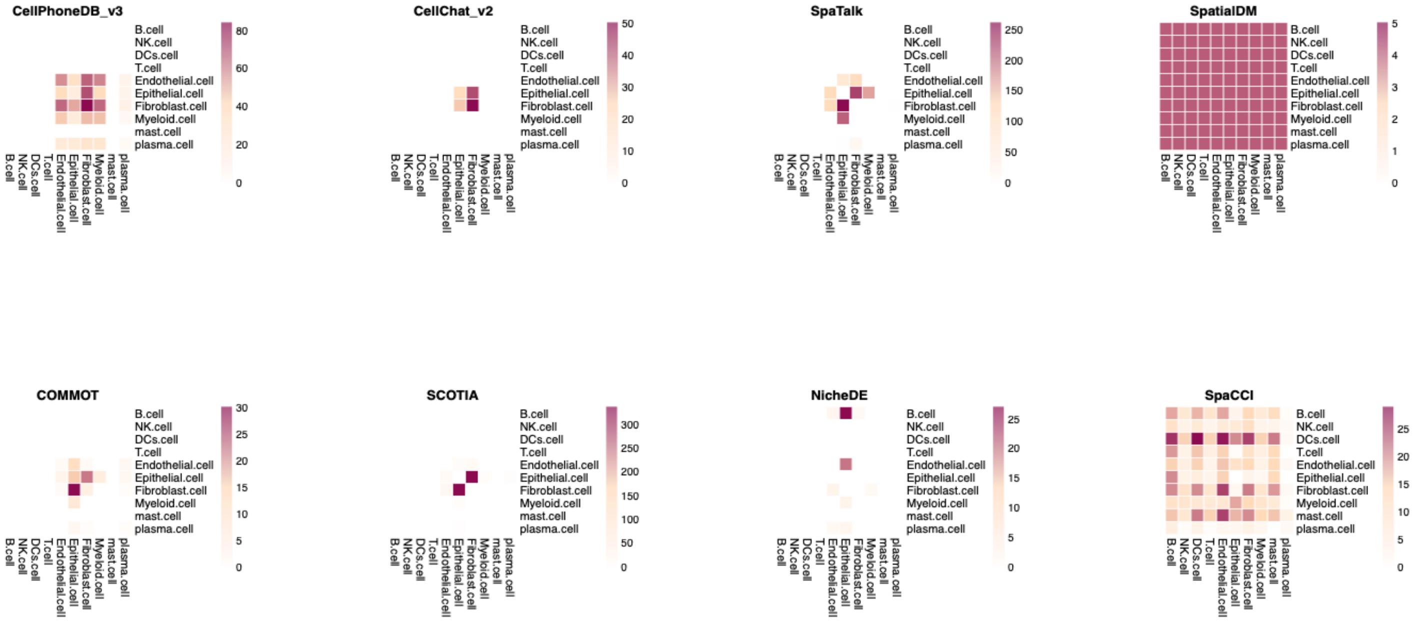

Fig. S11 | Cell-cell interaction inference in the 10x Visium IPMN dataset (sample HG1: High Grade IPMN). Heatmaps showing the number of inferred ligand–receptor interactions between cell type pairs, as detected by each of the eight computational tools. Rows represent sender cell types, and columns represent receiver cell types. The color intensity reflects the number of detected interactions.

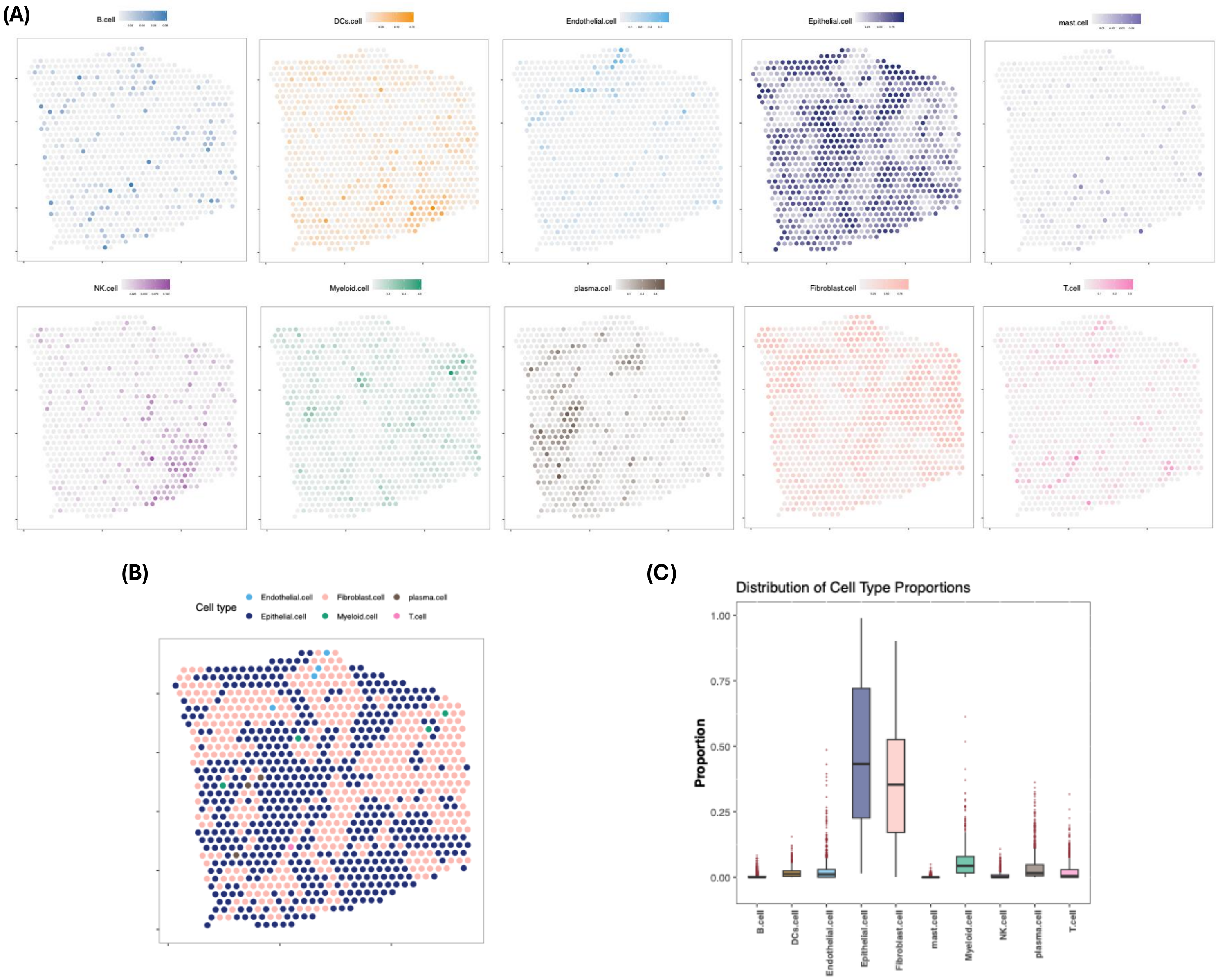

Fig. S12 | Cell type composition in the 10x Visium IPMN dataset (sample PDAC3: IPMN Associated Cancer). (A) Spatial distribution of cell type proportions across the tissue slide, showing estimated abundance for each major cell type per spot. (B) Spot-wise cell type annotation for methods that assume a single dominant cell type per spot. Each spot is labeled with the most abundant cell type. (C) Boxplot showing the distribution of cell type proportions across all spots in the sample.

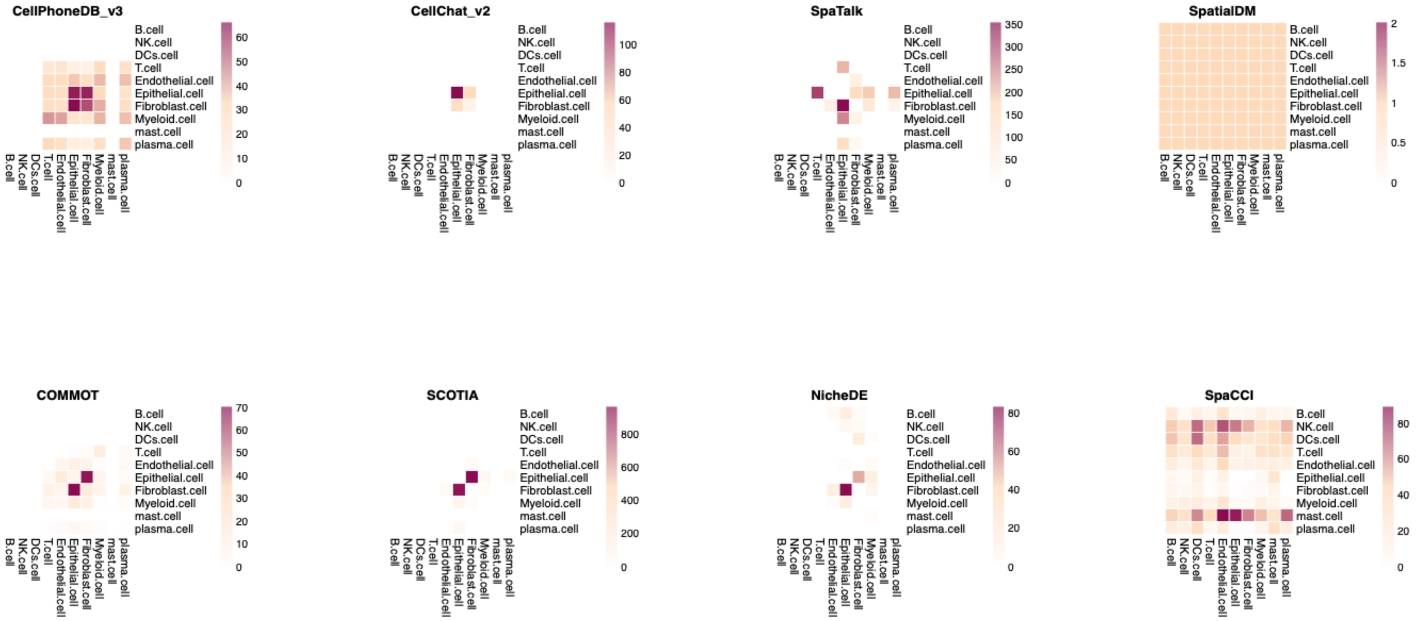

Fig. S13 | Cell-cell interaction inference in the 10x Visium IPMN dataset (sample PDAC3: IPMN Associated Cancer). Heatmaps showing the number of inferred ligand–receptor interactions between cell type pairs, as detected by each of the eight computational tools. Rows represent sender cell types, and columns represent receiver cell types. The color intensity reflects the number of detected interactions.

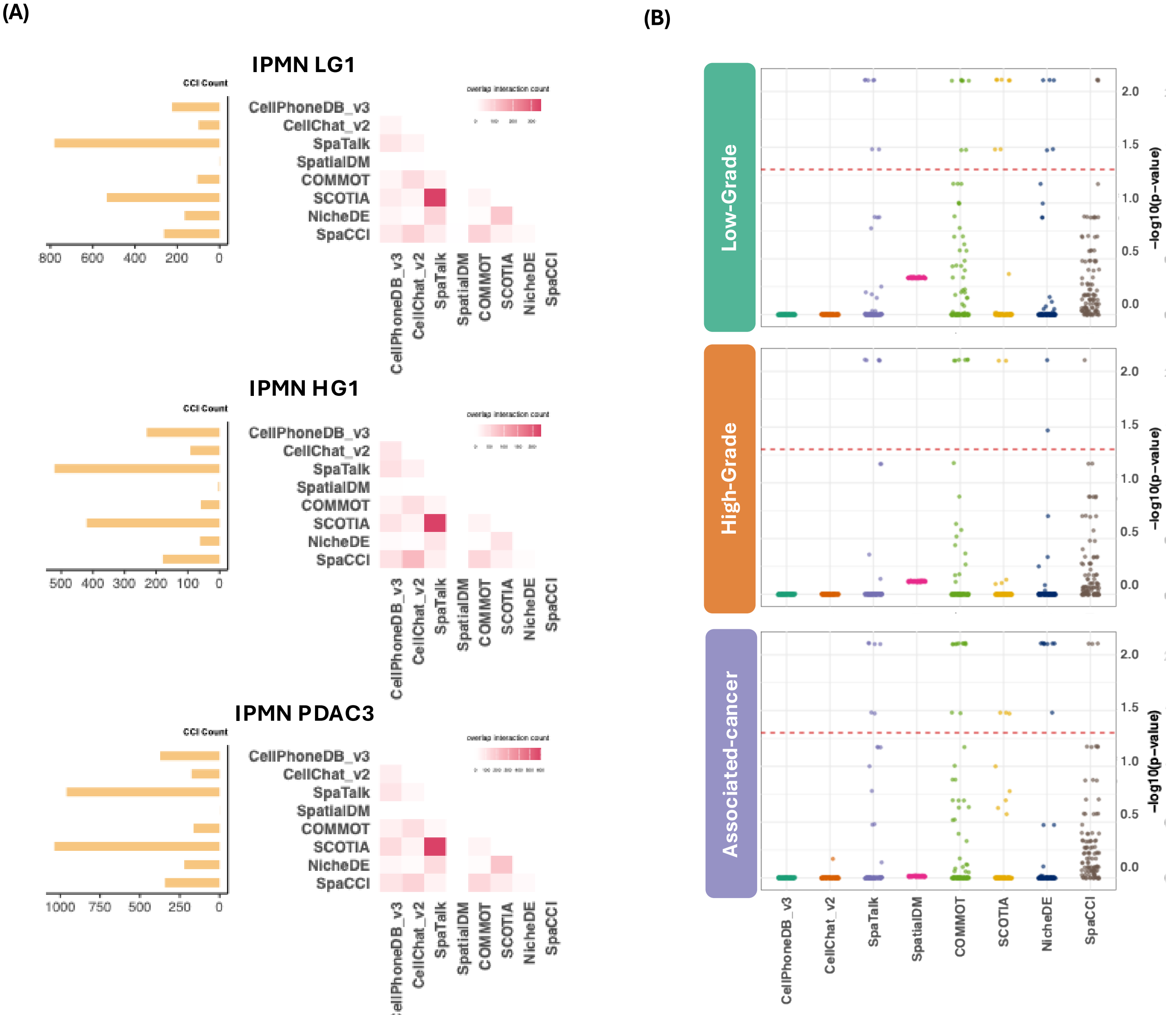

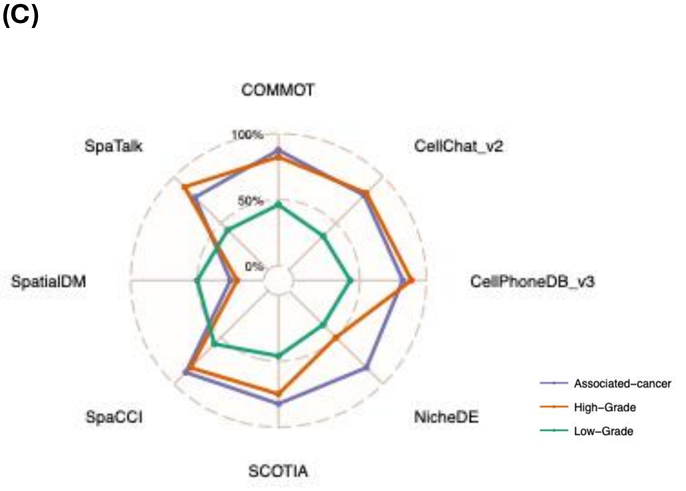

Fig. S14| Evaluation of cell-cell interaction inference in the 10x Visium IPMN dataset. (A) Bar plot showing the number of detected interactions and a heatmap of overlap counts between methods. (B) Evaluation of spatial leverage performance for each cell–cell interaction inference tool on 10x Visium IPMN data. The red dotted line indicates the significance threshold at $p = 0.05$ ($-\log_{10}(p) \approx 1.3$). Tools that incorporate spatial information tend to show a greater number of interactions above this threshold, reflecting their ability to detect spatially structured cell–cell communication. (C) Radar plots showing F1 score performance under different IPMN pathological condition.

(A)

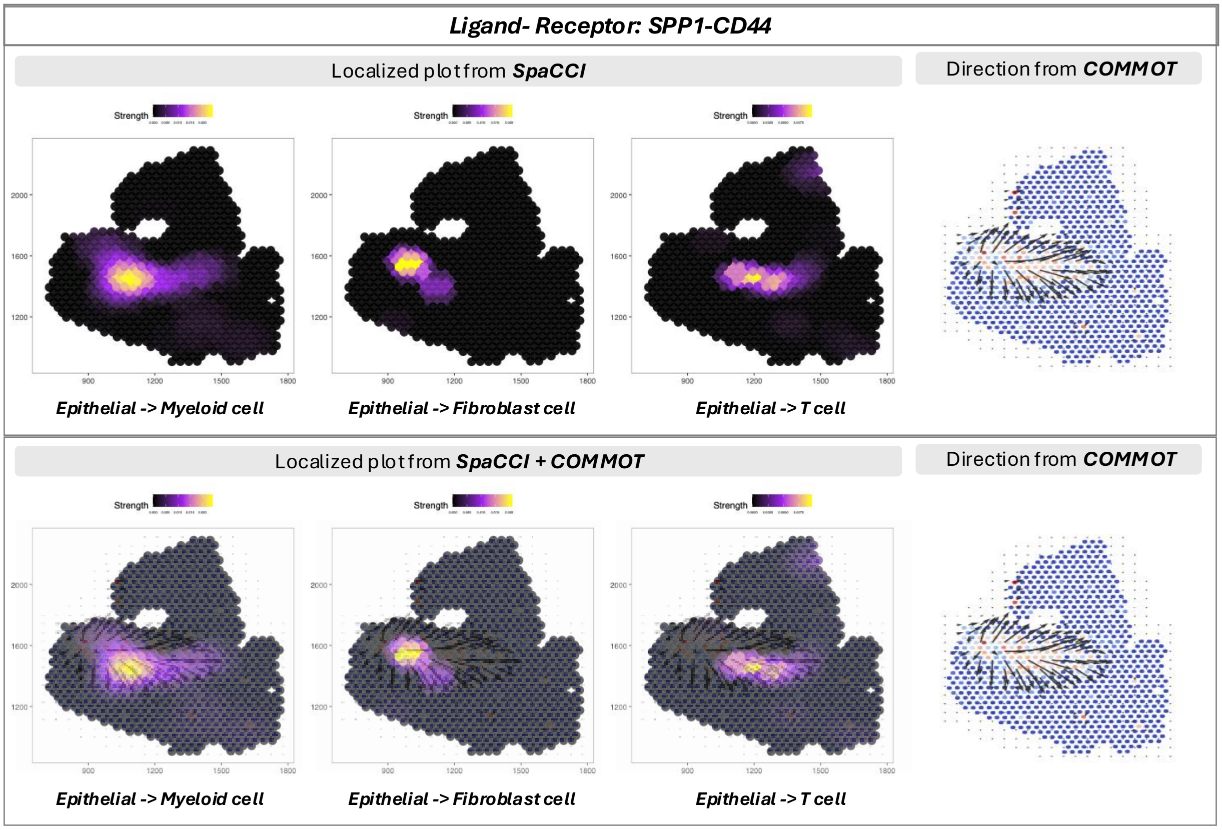

(B)

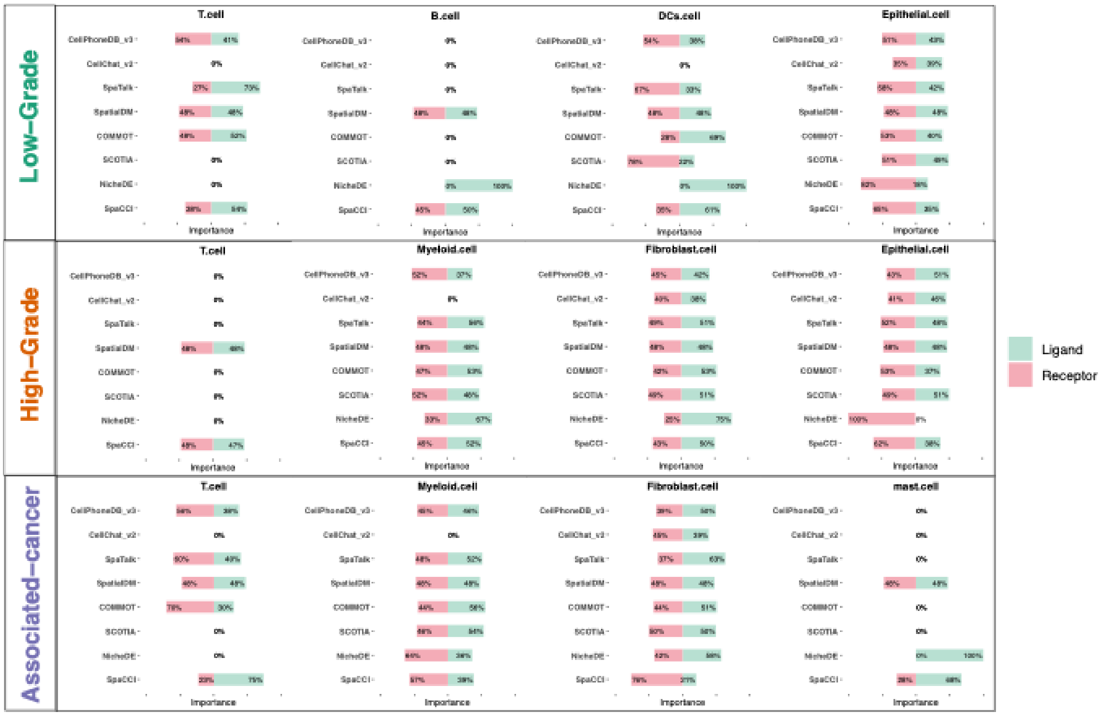

Fig. S15 | Evaluation of cell-cell interaction inference in the 10x Visium IPMN dataset. (A) Top panel: Localized interaction strength plots of low-grade IPMN (LG1) inferred by SpaCCI for three epithelial-directed interactions: Epithelial → Myeloid cell, Epithelial → Fibroblast cell, and Epithelial → T cell, alongside the overall directionality inferred by COMMOT. Bottom panel: Overlay of SpaCCI-inferred local signaling strength and COMMOT-inferred directionality for the same interactions, highlighting both spatial localization and direction of communication. (B) Comparison of ligand and receptor importance across cell types involved in cell–cell interactions in IPMN, stratified by pathological condition (Low-Grade, High-Grade, and Associated-Cancer).

(A)

(B)

(C)

(D)

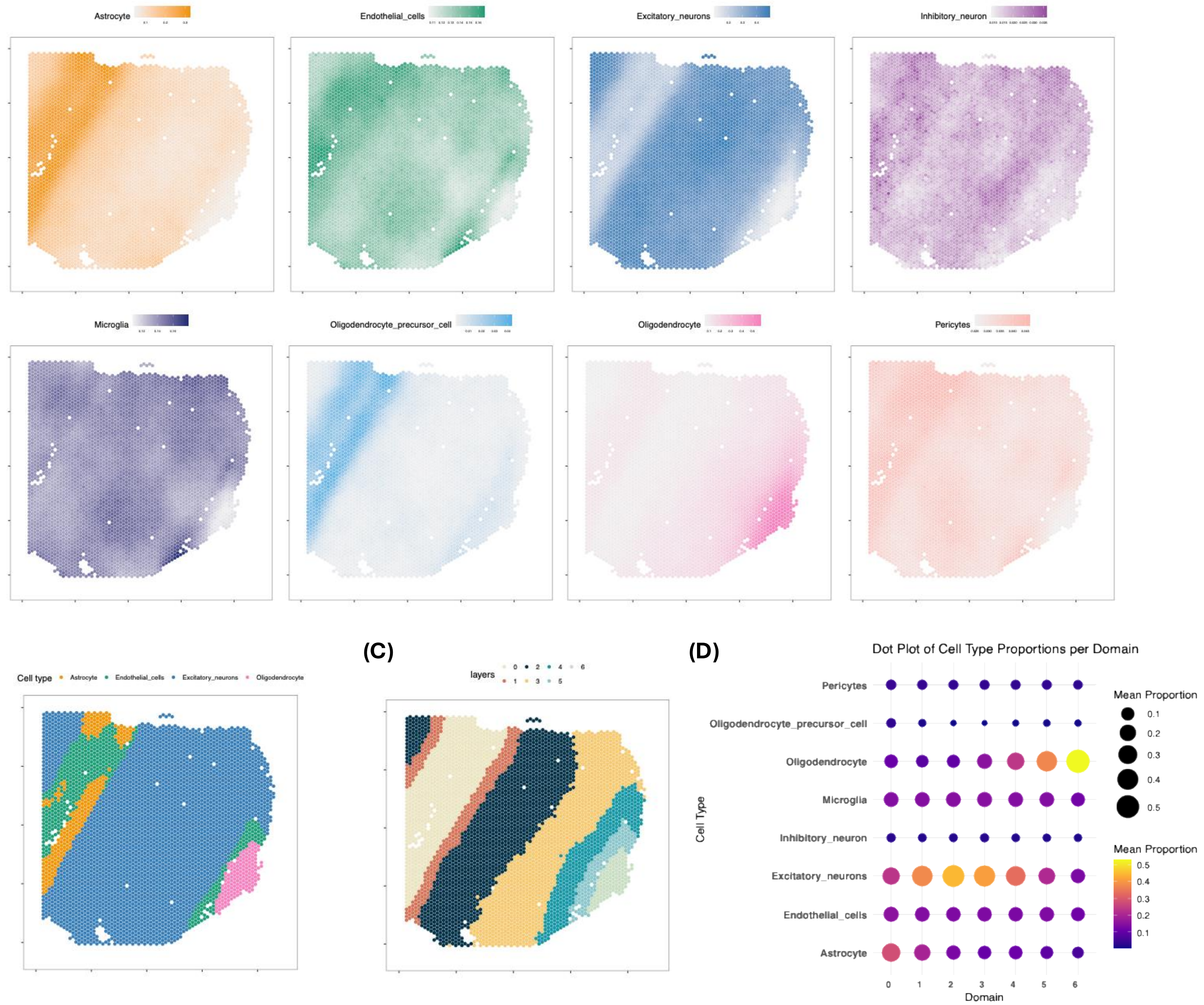

(E)

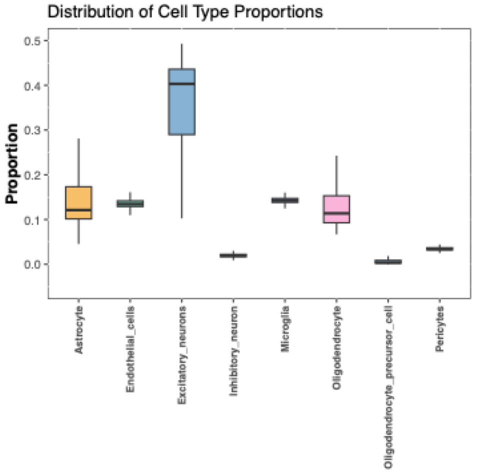

(F)

Metrics Table

Method	TP	FN	FP	TN
CellPhoneDB_v3	12	22	3	75
CellChat_v2	4	30	2	76
SpaTalk	2	32	3	75
SpatialDM	6	28	12	66
COMMOT	10	24	12	66
SCOTIA	22	12	55	23
NicheDE	1	33	0	78
SpaCCI	18	16	23	55

(G)

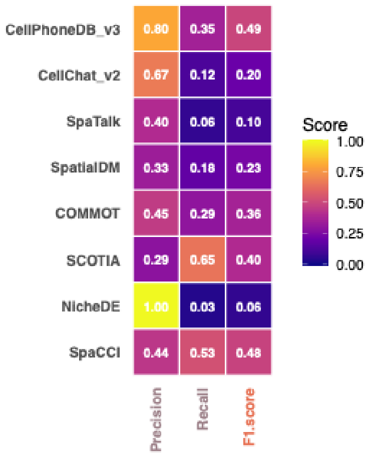

Fig. S16 | Cell type composition and evaluation of cell–cell interaction inference in the 10x Visium DLPFC dataset (slice 151507). (A) Spatial distribution of cell type proportions across the tissue slide, showing estimated abundance of each major cell type per spot. (B) Spot-wise cell type annotation for methods that assume a single dominant cell type per spot. Each spot is labeled according to the most abundant cell type. (C) Layer domain annotation across the DLPFC tissue. (D) Dot plot showing average cell type proportions within each domain layer. Dot size reflects the mean proportion of the respective cell type per layer. (E) Boxplot showing the distribution of cell type proportions across all spots. (F) Performance metrics table reporting true positives (TP), false negatives (FN), false positives (FP), and true negatives (TN) for each method, based on overlap with literature-supported interactions. (G) Heatmap summarizing precision, recall, and F1 score for each tool, based on pathway enrichment analysis compared to literature-supported pathways.

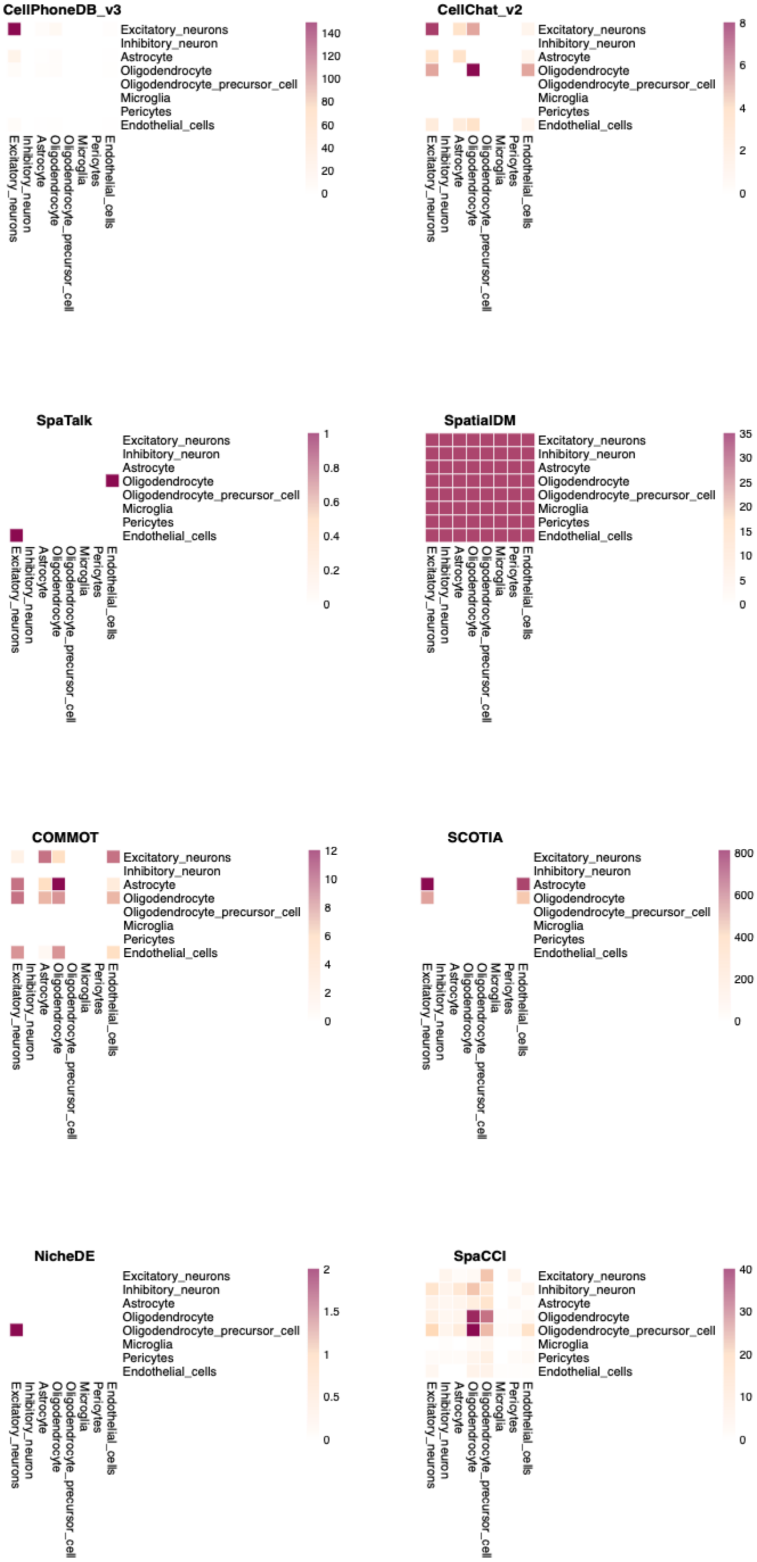

Fig. S17 | Cell-cell interaction inference in the 10x Visium DLPFC dataset (slice 151507). Heatmaps showing the number of inferred ligand–receptor interactions between cell type pairs, as detected by each of the eight computational tools. Rows represent sender cell types, and columns represent receiver cell types. The color intensity reflects the number of detected interactions.

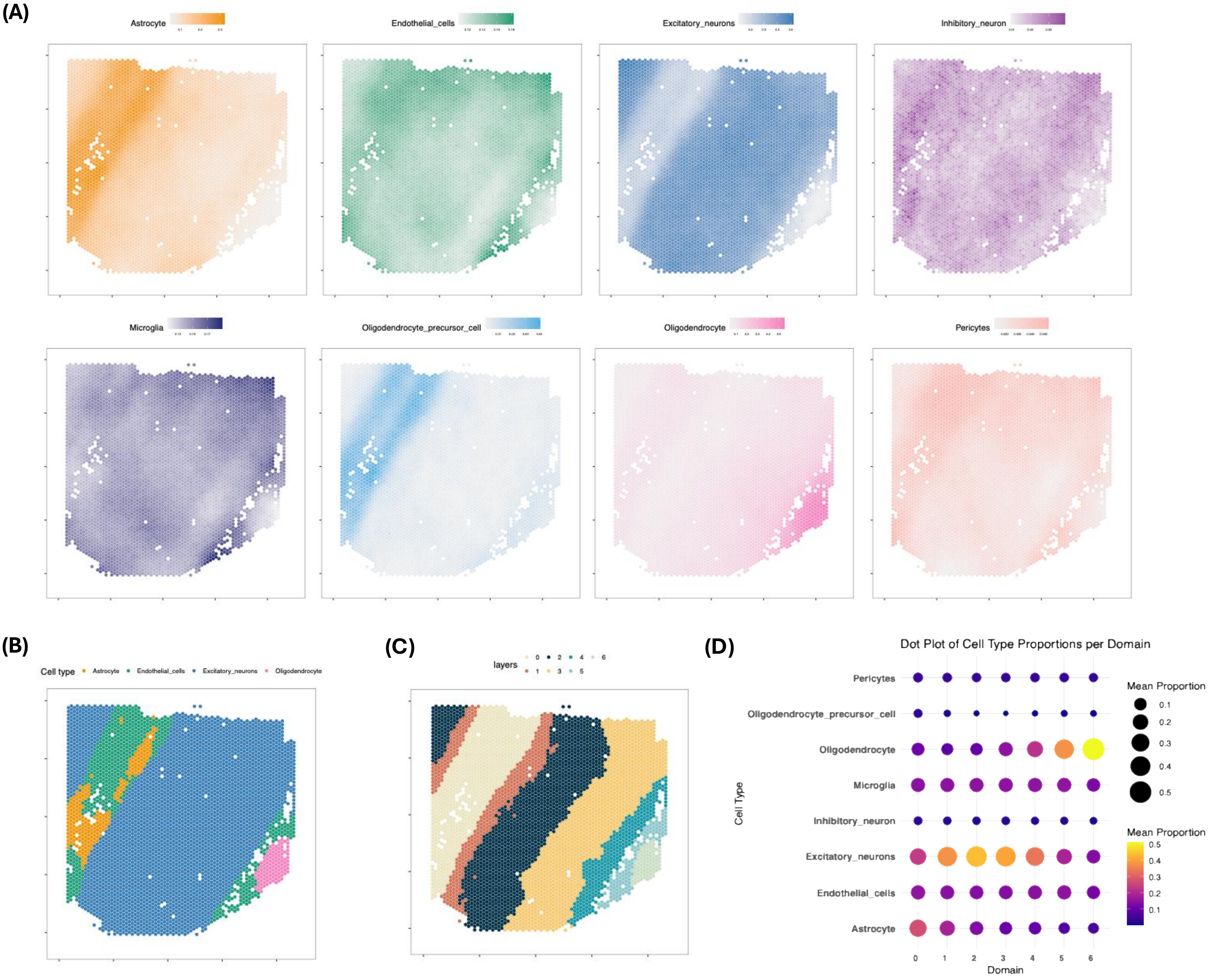

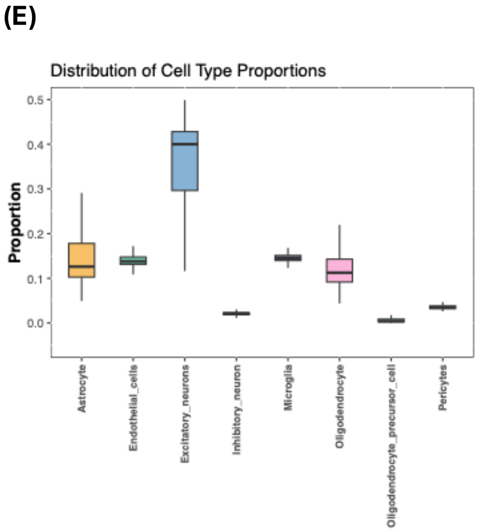

(F)

Metrics Table

Method	TP	FN	FP	TN
CellPhoneDB_v3	12	22	5	73
CellChat_v2	0	34	0	78
SpaTalk	2	32	6	72
SpatialDM	7	27	21	57
COMMOT	10	24	15	63
SCOTIA	23	11	55	23
NicheDE	5	29	1	77
SpaCCI	16	18	19	59

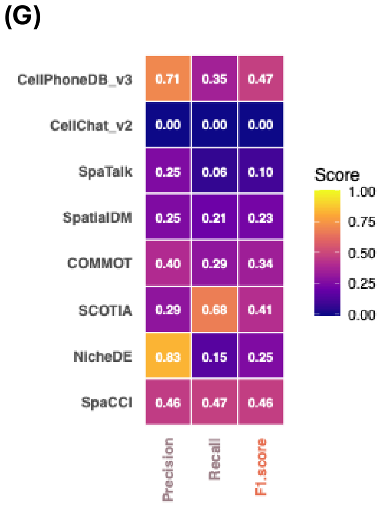

Fig. S18 | Cell type composition and evaluation of cell–cell interaction inference in the 10x Visium DLPFC dataset (slice 151508). (A) Spatial distribution of cell type proportions across the tissue slide, showing estimated abundance of each major cell type per spot. (B) Spot-wise cell type annotation for methods that assume a single dominant cell type per spot. Each spot is labeled according to the most abundant cell type. (C) Layer domain annotation across the DLPFC tissue. (D) Dot plot showing average cell type proportions within each domain layer. Dot size reflects the mean proportion of the respective cell type per layer. (E) Boxplot showing the distribution of cell type proportions across all spots. (F) Performance metrics table reporting true positives (TP), false negatives (FN), false positives (FP), and true negatives (TN) for each method, based on overlap with literature-supported interactions. (G) Heatmap summarizing precision, recall, and F1 score for each tool, based on pathway enrichment analysis compared to literature-supported pathways.

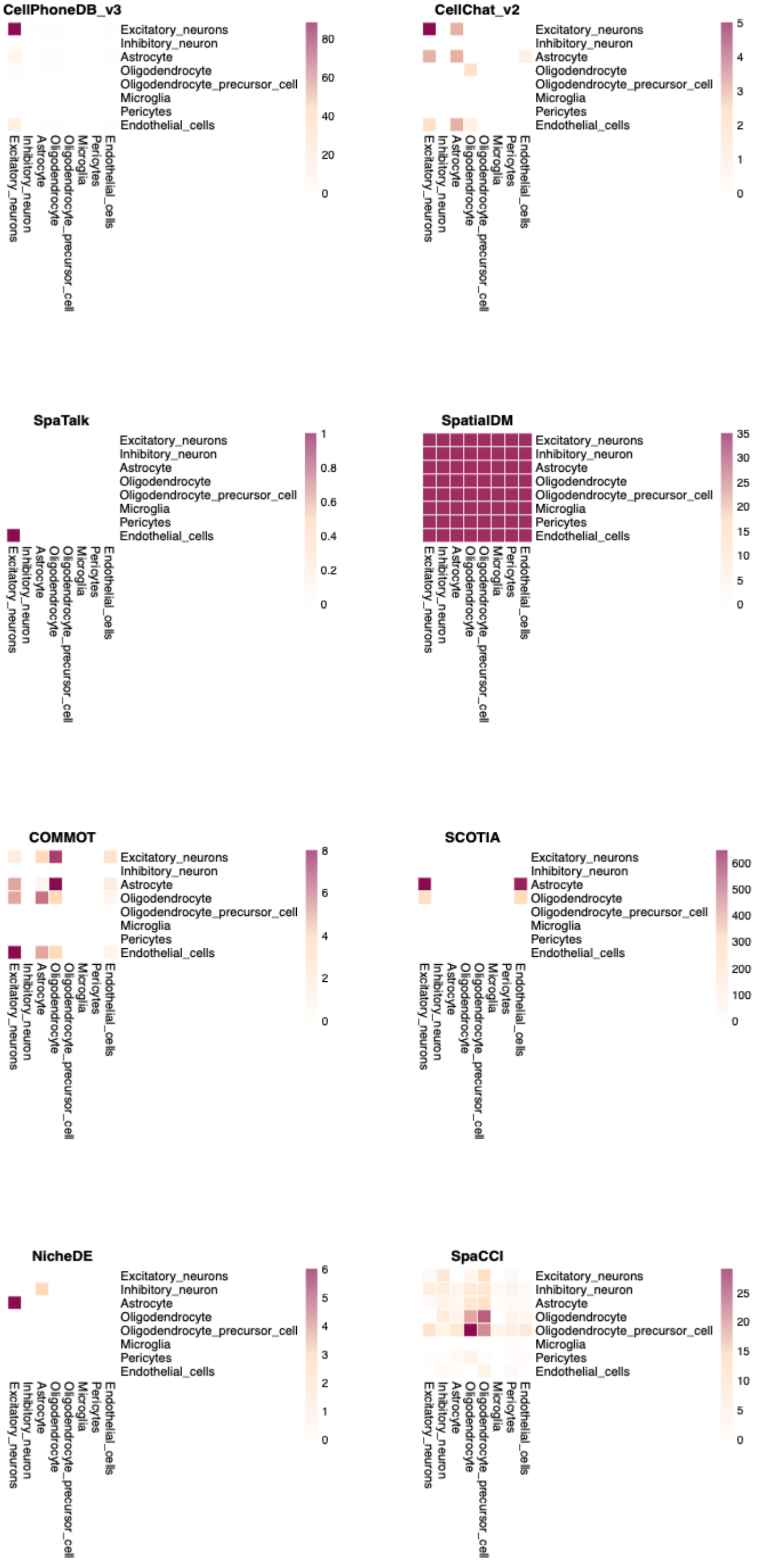

Fig. S19 | Cell-cell interaction inference in the 10x Visium DLPFC dataset (slice 151508). Heatmaps showing the number of inferred ligand–receptor interactions between cell type pairs, as detected by each of the eight computational tools. Rows represent sender cell types, and columns represent receiver cell types. The color intensity reflects the number of detected interactions.

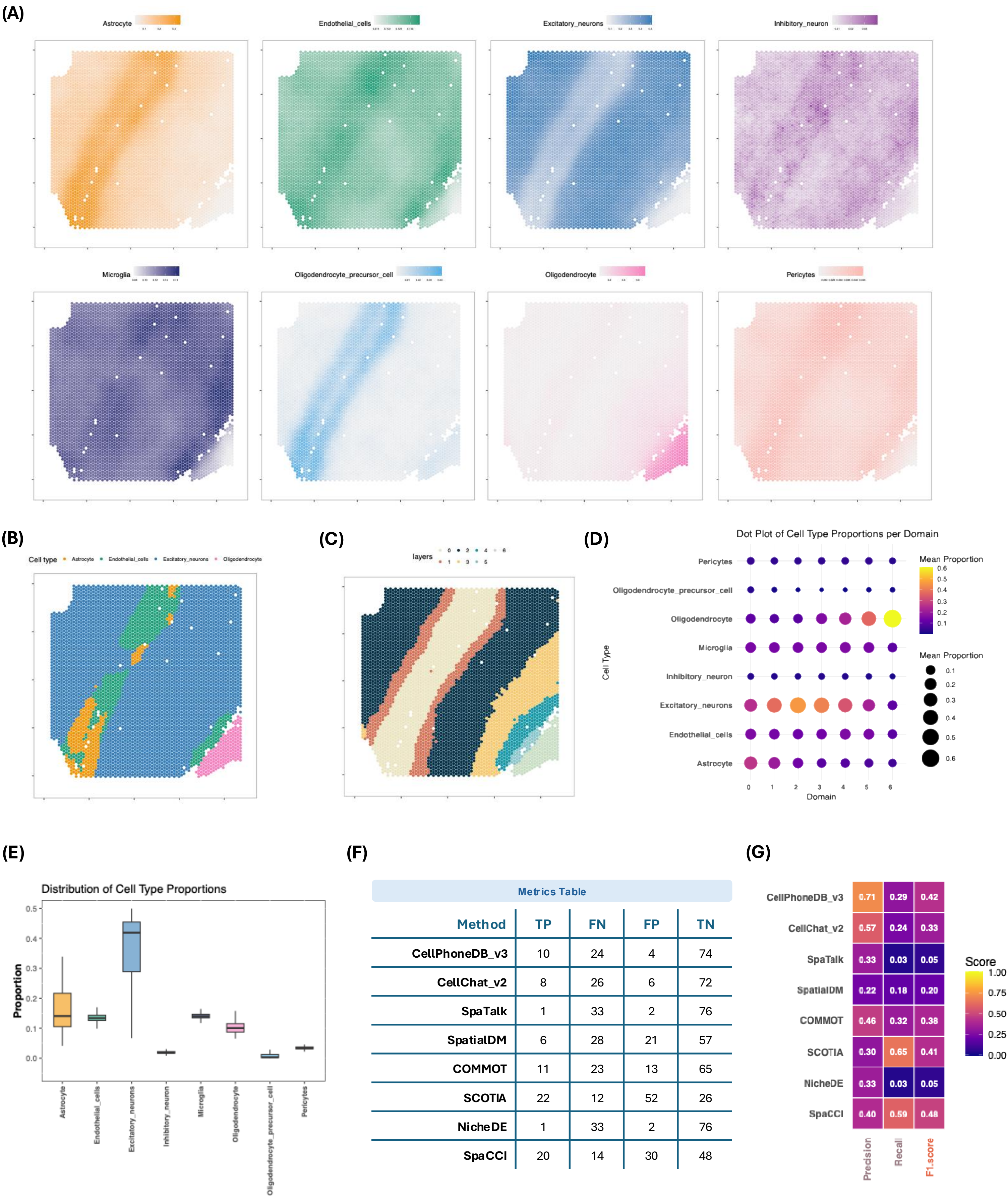

Metrics Table

Method	TP	FN	FP	TN
CellPhoneDB_v3	10	24	4	74
CellChat_v2	8	26	6	72
SpaTalk	1	33	2	76
SpatialDM	6	28	21	57
COMMOT	11	23	13	65
SCOTIA	22	12	52	26
NicheDE	1	33	2	76
SpaCCI	20	14	30	48

	Precision	Recall	F1.score
CellPhoneDB_v3	0.71	0.29	0.42
CellChat_v2	0.57	0.24	0.33
SpaTalk	0.33	0.03	0.05
SpatialDM	0.22	0.18	0.20
COMMOT	0.46	0.32	0.38
SCOTIA	0.30	0.65	0.41
NicheDE	0.33	0.03	0.05
SpaCCI	0.40	0.59	0.48

Fig. S20 | Cell type composition and evaluation of cell–cell interaction inference in the 10x Visium DLPFC dataset (slice 151509). (A) Spatial distribution of cell type proportions across the tissue slide, showing estimated abundance of each major cell type per spot. (B) Spot-wise cell type annotation for methods that assume a single dominant cell type per spot. Each spot is labeled according to the most abundant cell type. (C) Layer domain annotation across the DLPFC tissue. (D) Dot plot showing average cell type proportions within each domain layer. Dot size reflects the mean proportion of the respective cell type per layer. (E) Boxplot showing the distribution of cell type proportions across all spots. (F) Performance metrics table reporting true positives (TP), false negatives (FN), false positives (FP), and true negatives (TN) for each method, based on overlap with literature-supported interactions. (G) Heatmap summarizing precision, recall, and F1 score for each tool, based on pathway enrichment analysis compared to literature-supported pathways.

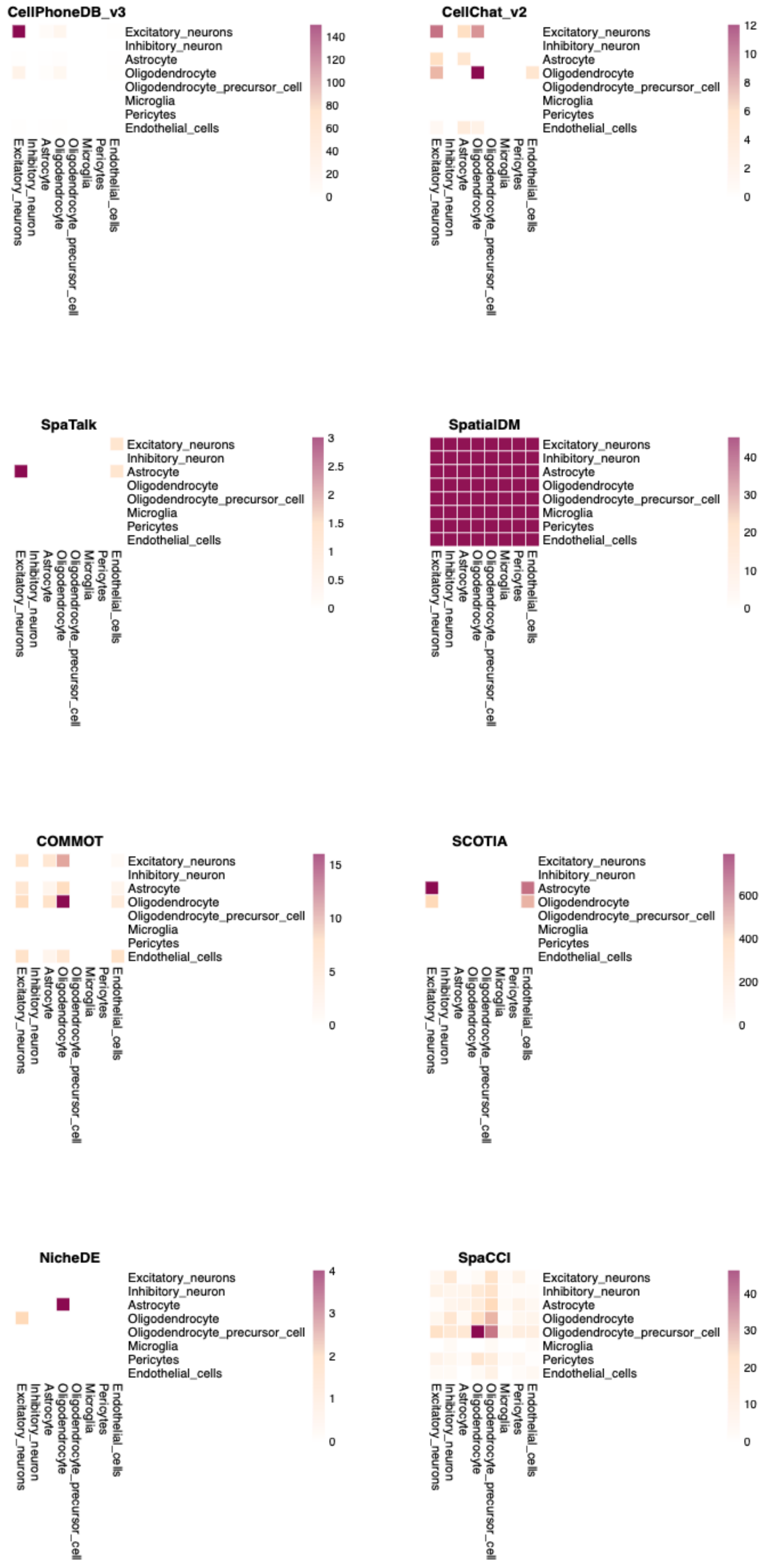

Fig. S21 | Cell-cell interaction inference in the 10x Visium DLPFC dataset (slice 151509). Heatmaps showing the number of inferred ligand–receptor interactions between cell type pairs, as detected by each of the eight computational tools. Rows represent sender cell types, and columns represent receiver cell types. The color intensity reflects the number of detected interactions.

(A)

(B)

(C)

(D)

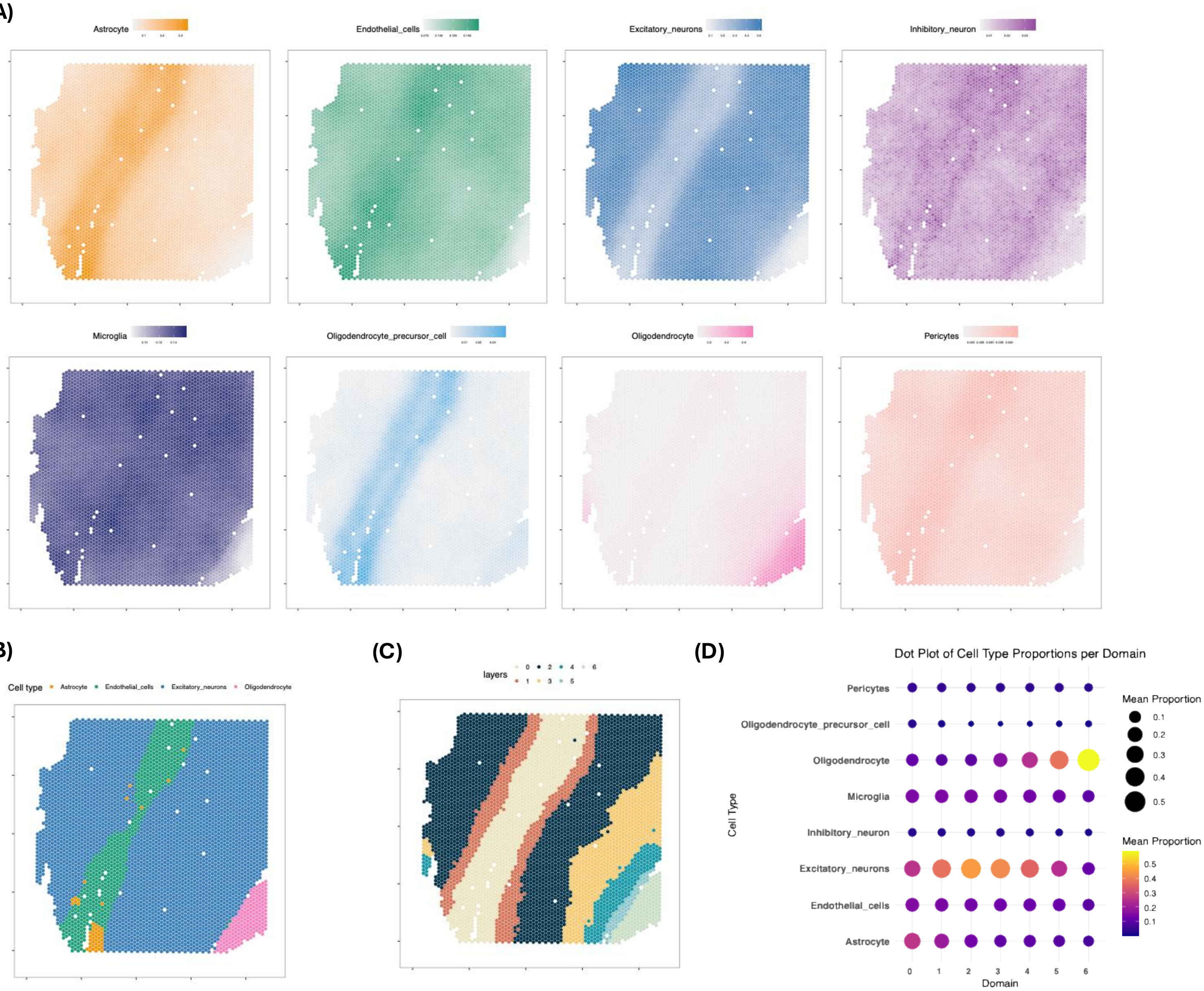

(E)

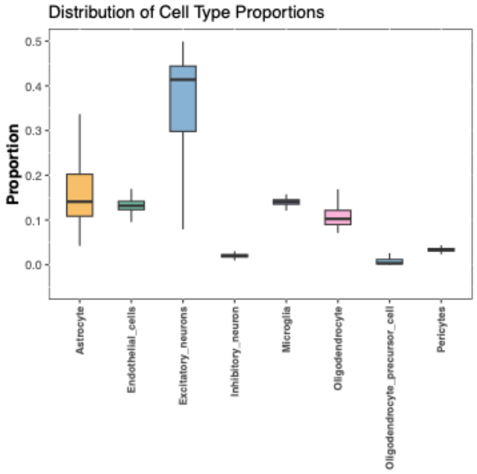

(F)

Metrics Table

Method	TP	FN	FP	TN
CellPhoneDB_v3	10	24	3	75
CellChat_v2	6	28	7	71
SpaTalk	14	20	36	42
SpatialDM	6	28	18	60
COMMOT	11	23	11	67
SCOTIA	22	12	52	26
NicheDE	0	34	0	78
SpaCCI	18	16	23	55

(G)

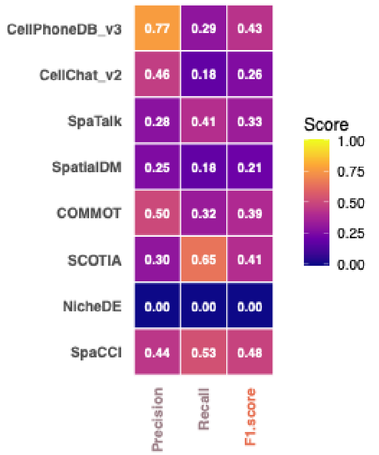

Fig. S22 | Cell type composition and evaluation of cell–cell interaction inference in the 10x Visium DLPFC dataset (slice 151510). (A) Spatial distribution of cell type proportions across the tissue slide, showing estimated abundance of each major cell type per spot. (B) Spot-wise cell type annotation for methods that assume a single dominant cell type per spot. Each spot is labeled according to the most abundant cell type. (C) Layer domain annotation across the DLPFC tissue. (D) Dot plot showing average cell type proportions within each domain layer. Dot size reflects the mean proportion of the respective cell type per layer. (E) Boxplot showing the distribution of cell type proportions across all spots. (F) Performance metrics table reporting true positives (TP), false negatives (FN), false positives (FP), and true negatives (TN) for each method, based on overlap with literature-supported interactions. (G) Heatmap summarizing precision, recall, and F1 score for each tool, based on pathway enrichment analysis compared to literature-supported pathways.

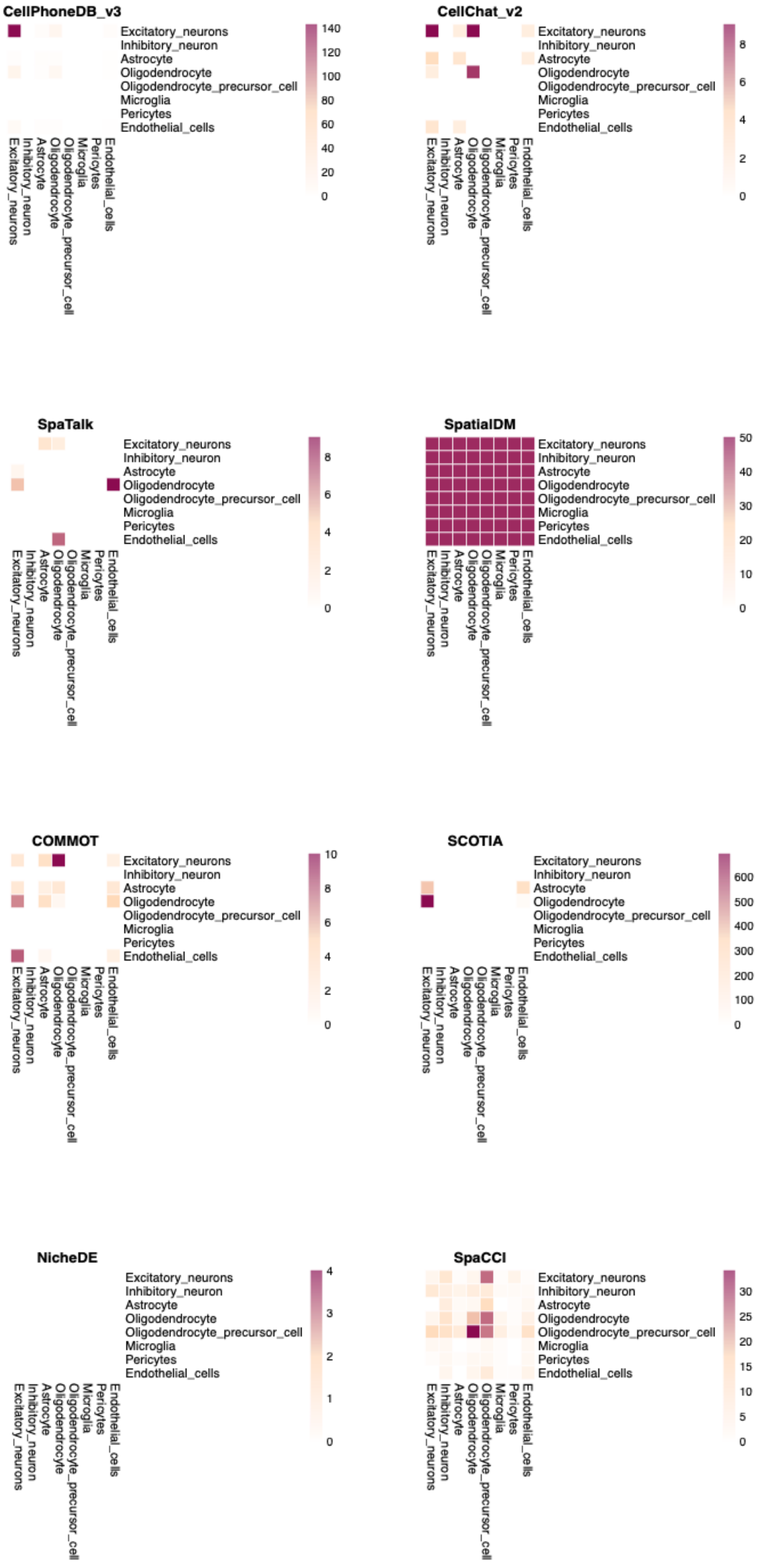

Fig. S23 | Cell-cell interaction inference in the 10x Visium DLPFC dataset (slice 151510). Heatmaps showing the number of inferred ligand–receptor interactions between cell type pairs, as detected by each of the eight computational tools. Rows represent sender cell types, and columns represent receiver cell types. The color intensity reflects the number of detected interactions.

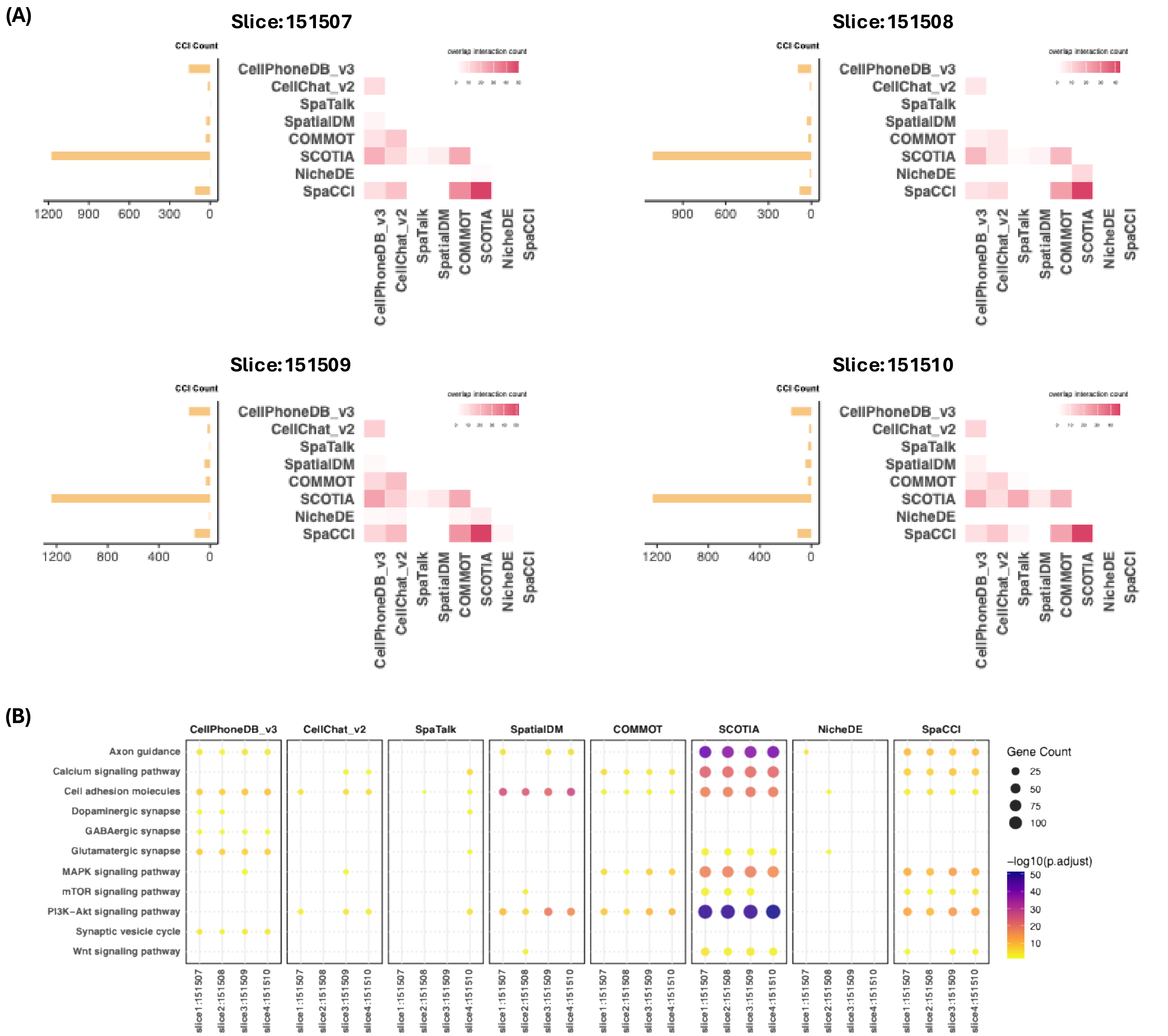

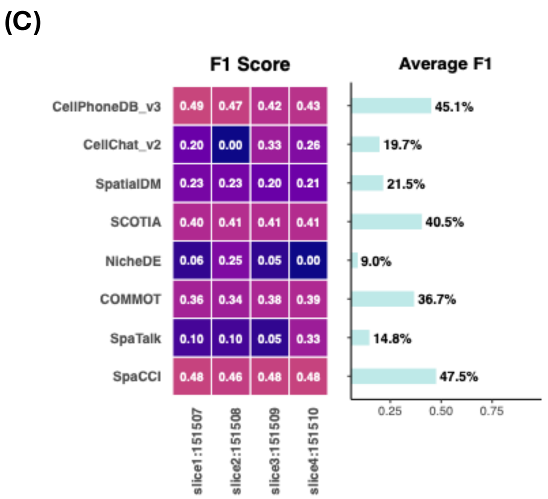

Fig. S24 | Evaluation of cell-cell interaction inference in the 10x Visium DLPFC dataset. (A) Bar plot showing the number of detected interactions and a heatmap of overlap counts between methods. (B) Pathway enrichment of ligand–receptor-mediated interactions across DLPFC slices as inferred by eight computational CCI tools. (C) Heatmap showing the F1 score for each slice and tool, based on pathway enrichment analysis on the DLPFC dataset. F1 scores reflect the agreement between inferred and literature-supported pathways. The accompanying bar plot summarizes the average F1 score across conditions for each method.

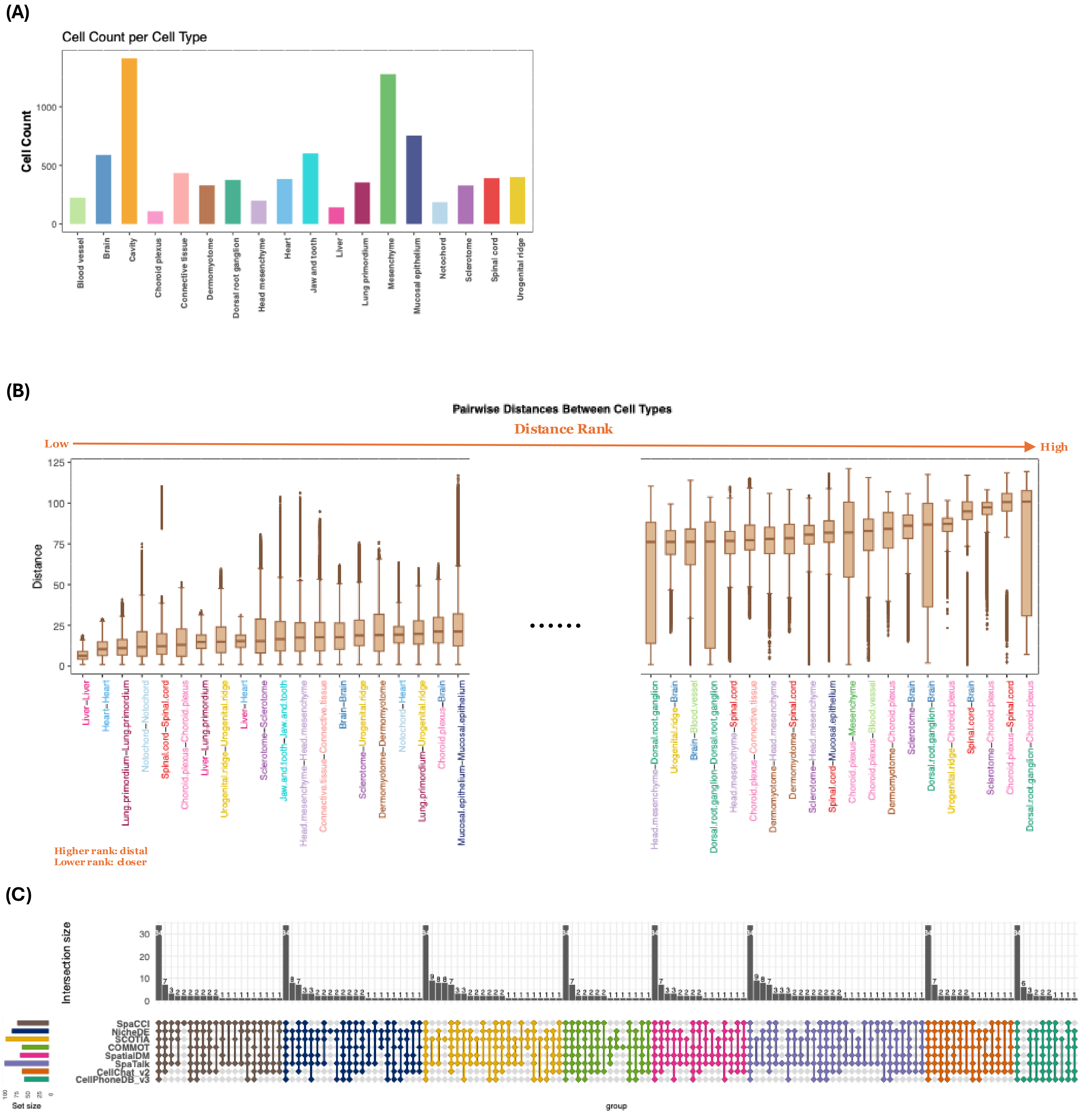

Fig. S25 | Cell–cell interaction distance and overlap analysis in the Stereo-Seq dataset (MOSTA E10.5). (A) Bar plot showing the number of cells per annotated cell type in the MOSTA E10.5 Stereo-seq data. (B) Boxplots illustrating pairwise spatial distances between cell type clusters, used to define interaction distance ranks in the analysis. Lower ranks correspond to proximal interactions between nearby cell types; higher ranks represent more spatially distant interactions. (C) UpSet plot showing the overlap of detected interactions across the eight computational tools applied to the MOSTA Stereo-seq dataset.

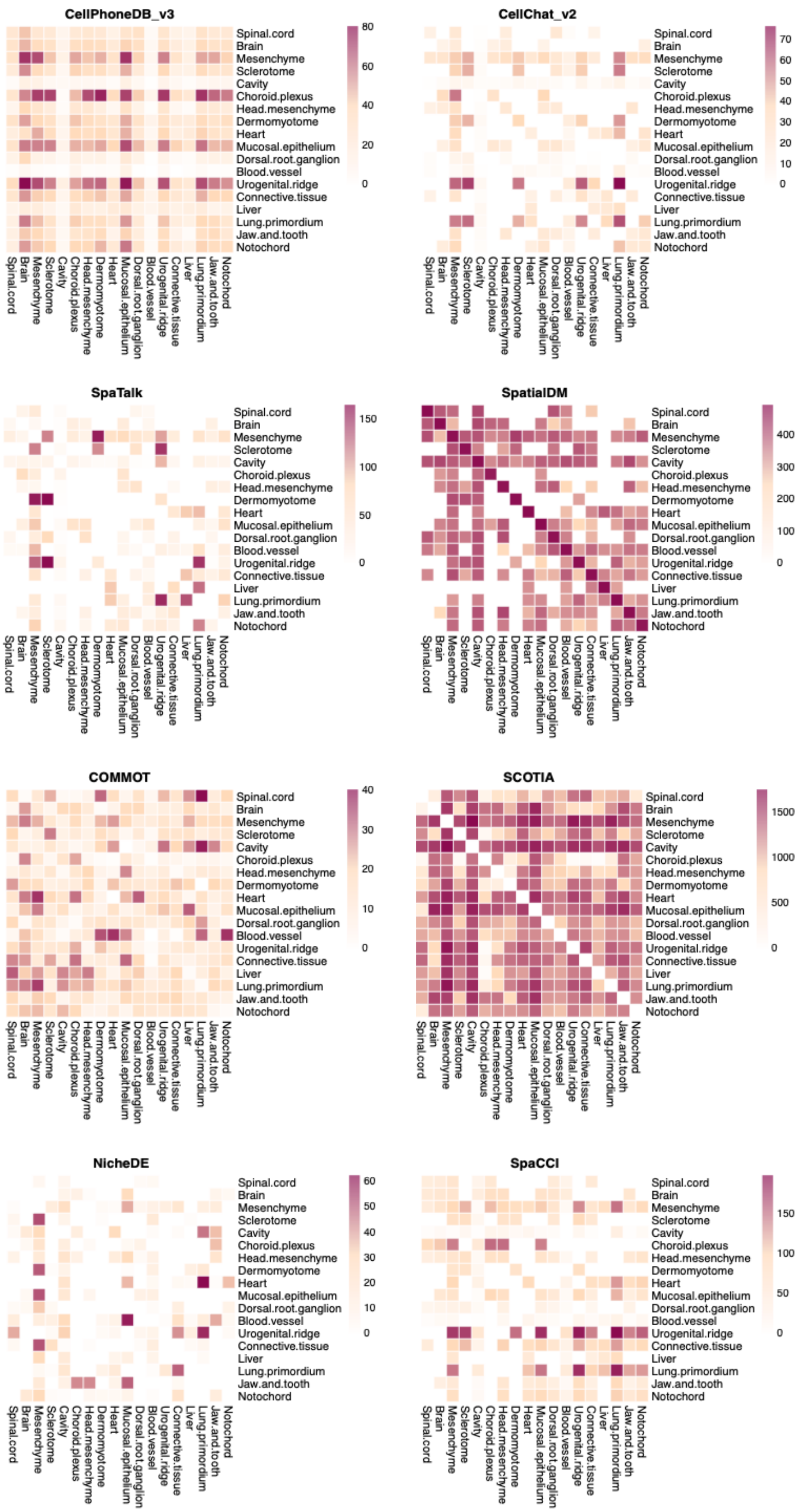

Fig. S26 | Cell-cell interaction inference in the Stereo-Seq dataset (MOSTA E10.5). Heatmaps showing the number of inferred ligand–receptor interactions between cell type pairs, as detected by each of the eight computational tools. Rows represent sender cell types, and columns represent receiver cell types. The color intensity reflects the number of detected interactions.

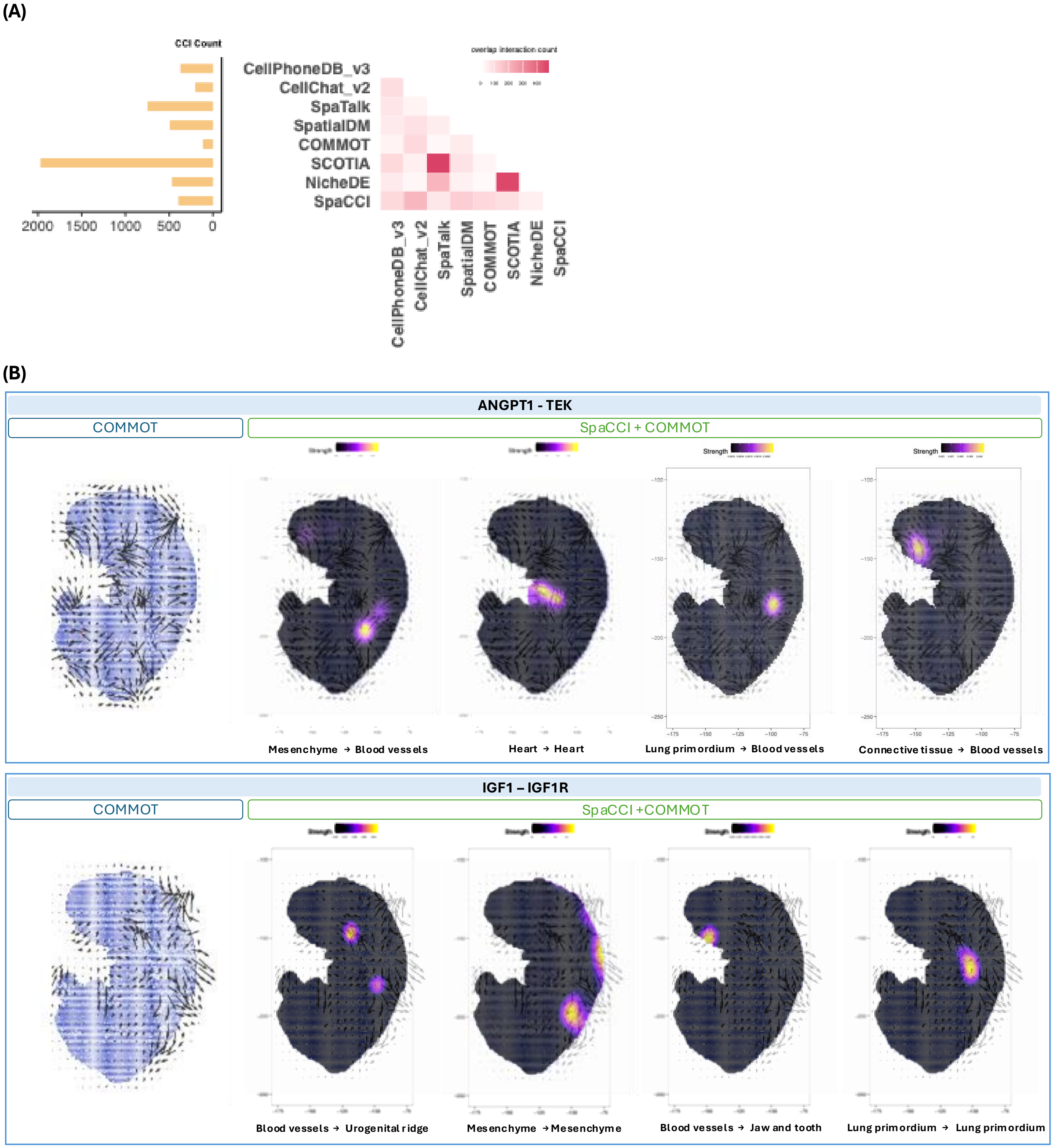

Fig. S27 | Evaluation of cell-cell interaction inference in the Stereo-Seq dataset (MOSTA E10.5). (A) Bar plot showing the number of detected interactions and a heatmap of overlap counts between methods. (B) Overlay of spatial visualization of selected interactions. Top panel: ANGPT1 - TEK interaction, showing global directionality (COMMOT) and local cell-type-specific signaling strength (SpaCCI). Bottom panel: IGF1 - IGF1R interaction, with similar visualization of directionality and strength.

(A)

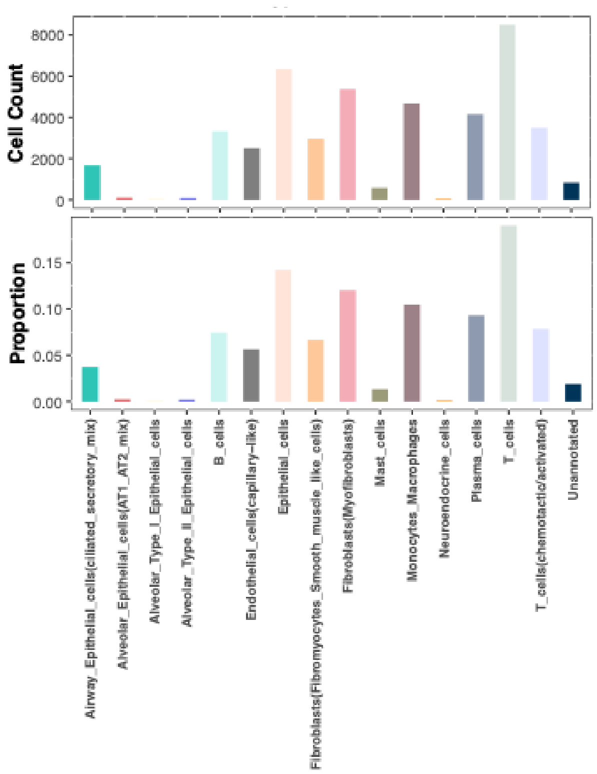

(B)

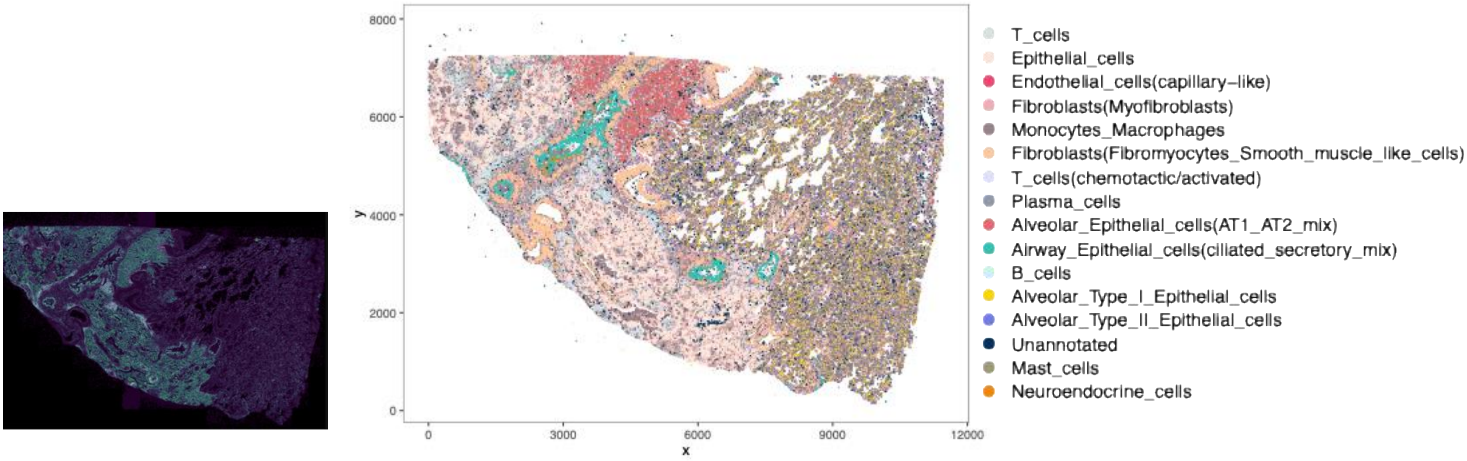

Fig. S28 | Cell type composition in the Xenium 5K FFPE lung cancer dataset.
(A) Bar plots showing the total cell count (top) and relative proportion (bottom) of each annotated cell type across the Xenium 5K FFPE lung cancer dataset. (B) Spatial visualization of the tissue section, including a fluorescence image (left) and a cell-type annotated map (right), showing the spatial distribution of major immune, stromal, and epithelial cell types.

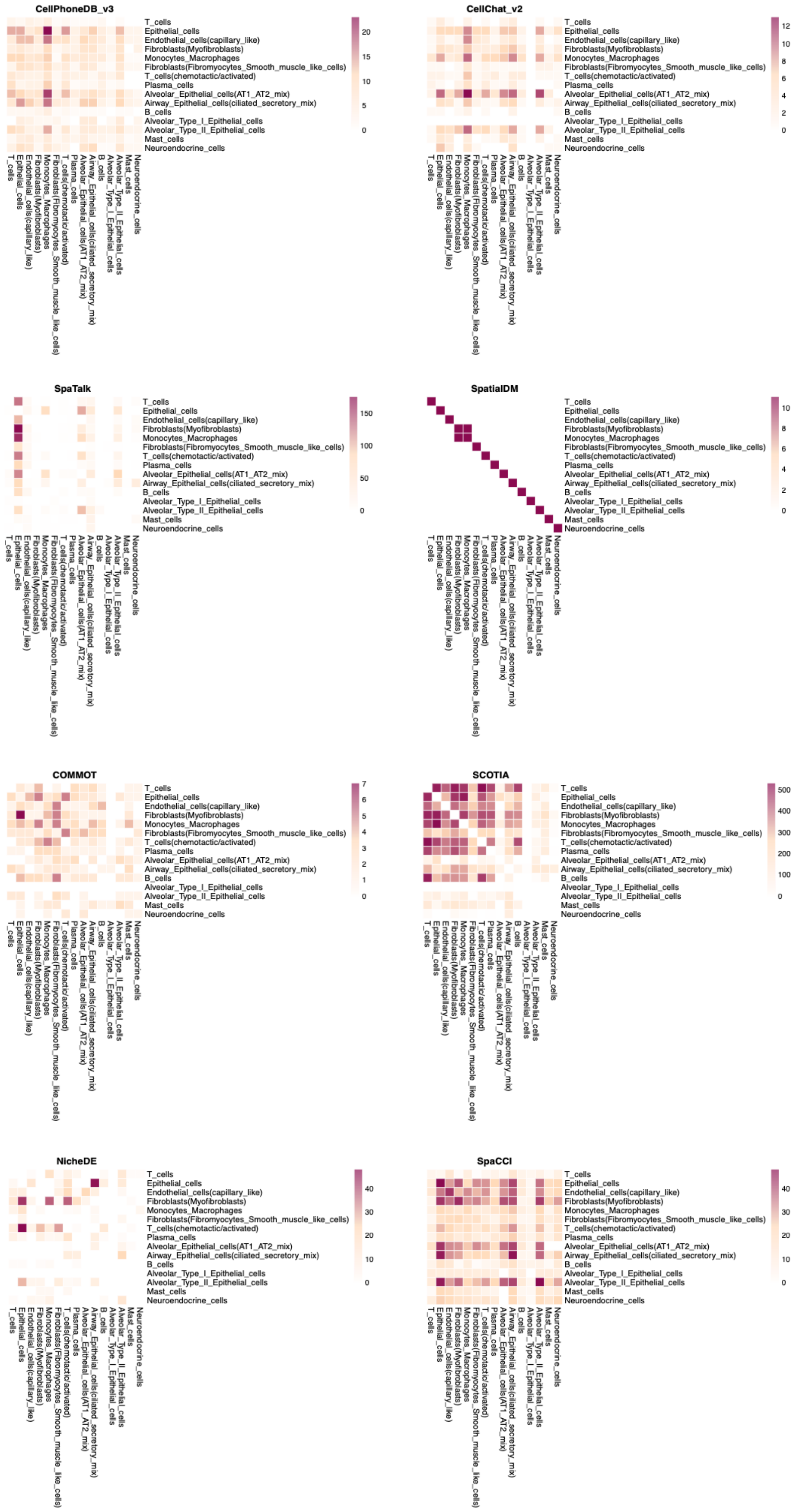

Fig. S29 | Cell-cell interaction inference in the Xenium 5K FFPE lung cancer dataset. Heatmaps showing the number of inferred ligand–receptor interactions between cell type pairs, as detected by each of the eight computational tools. Rows represent sender cell types, and columns represent receiver cell types. The color intensity reflects the number of detected interactions.

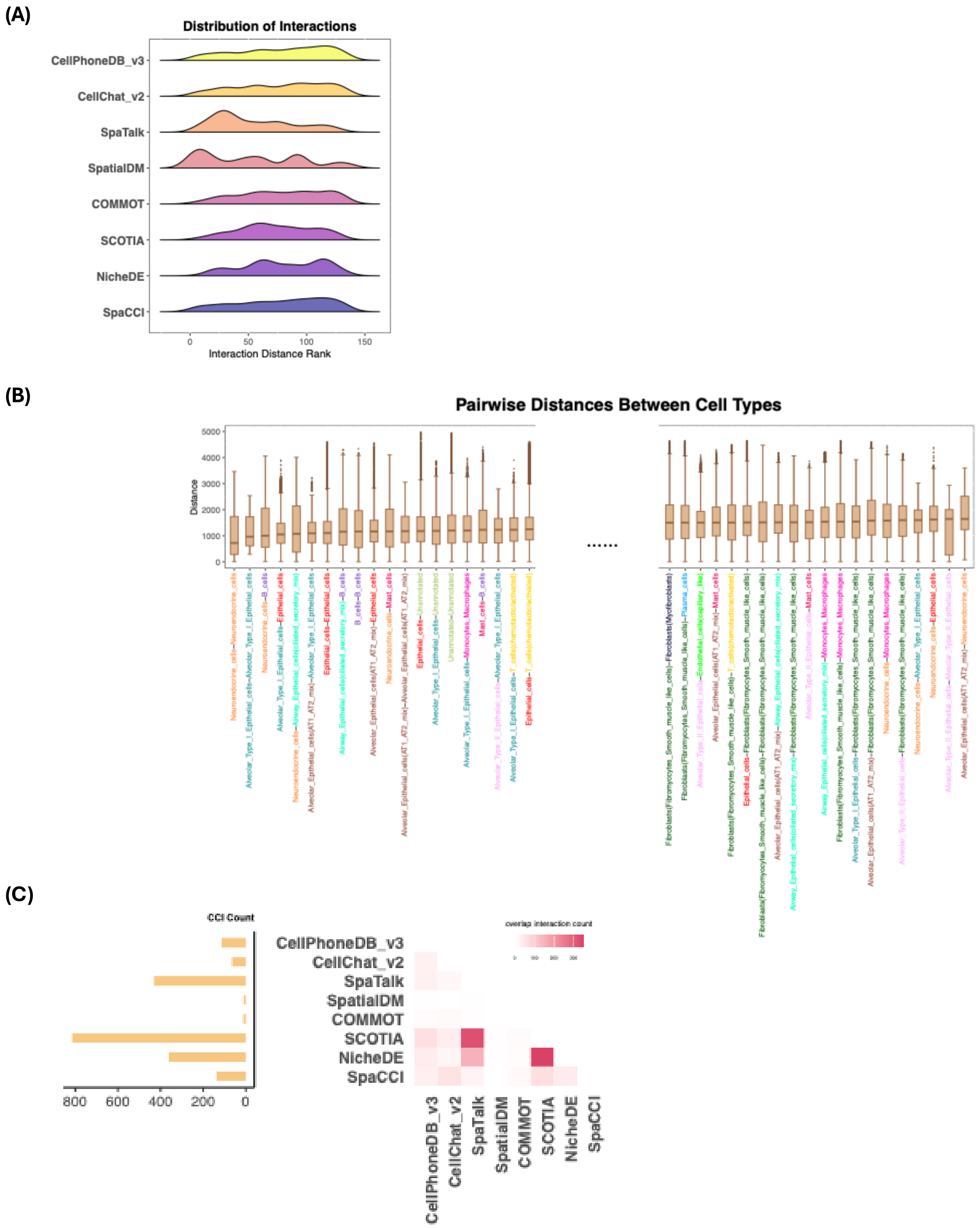

Fig. S30 | Evaluation of cell–cell interaction inference in the Xenium 5K FFPE lung cancer dataset. (A) Ridge plots showing the distribution of interaction distances inferred by each tool. On the x-axis, lower ranks indicate proximal interactions between cell types, while higher ranks reflect distal interactions. (B) Boxplots illustrating pairwise spatial distances between annotated cell type clusters, used to define interaction distance ranks in the analysis. While some variation exists across cell type pairs, the spatial separation is generally modest, suggesting that distinctions between proximal and distal pairs are not strongly pronounced.. (C) Bar plot showing the number of detected cell–cell interactions per method, alongside a heatmap of overlapping interaction counts between tools.

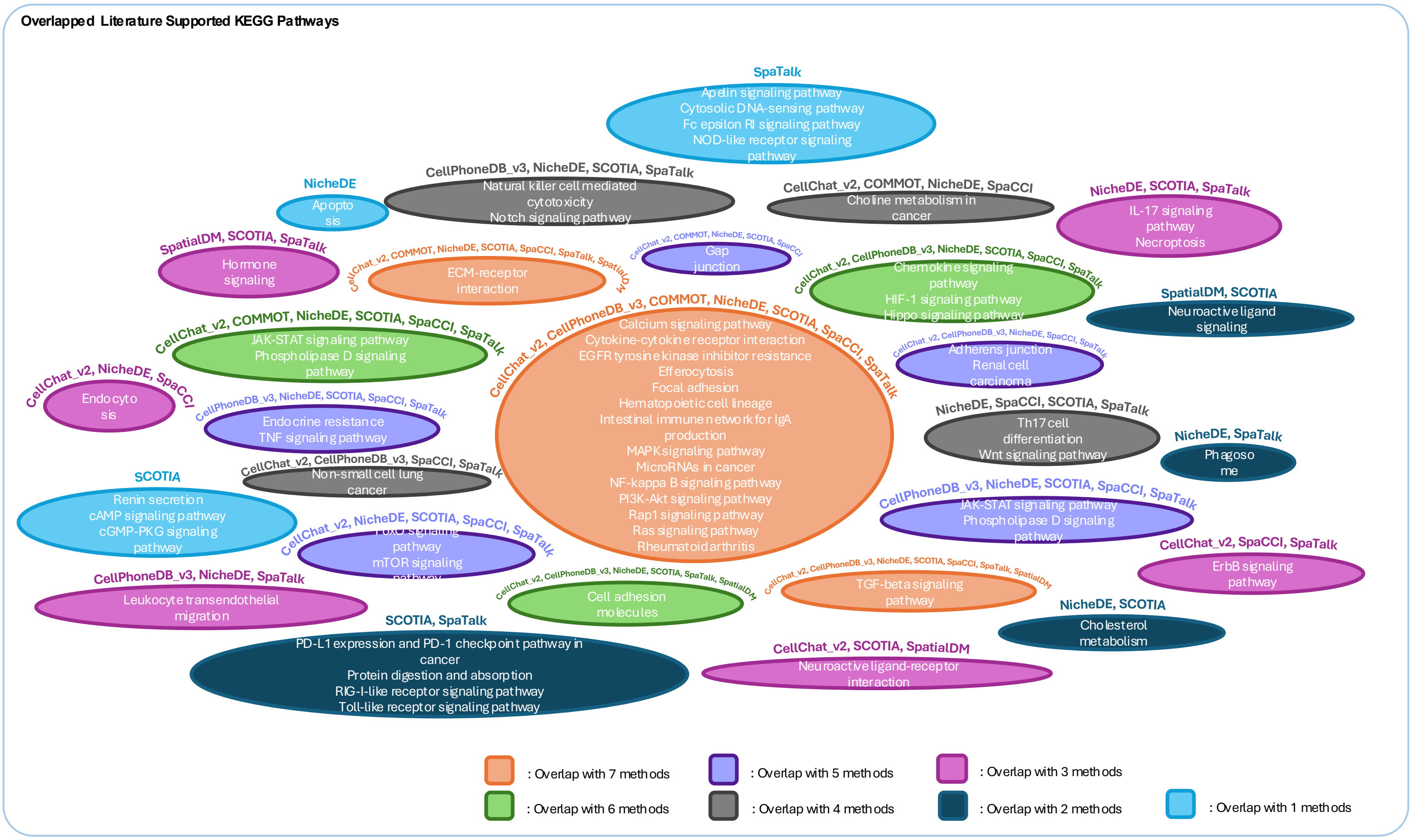

Fig. S31 | Evaluation of biological meaningful pathways in the Xenium 5K FFPE lung cancer dataset. Literature-supported KEGG pathways enriched among the inferred interactions. Each pathway is colored by the number of methods that commonly detect it, with larger overlaps (≥7 tools) shown in orange and smaller overlaps shown in shades of blue and purple.

(A)

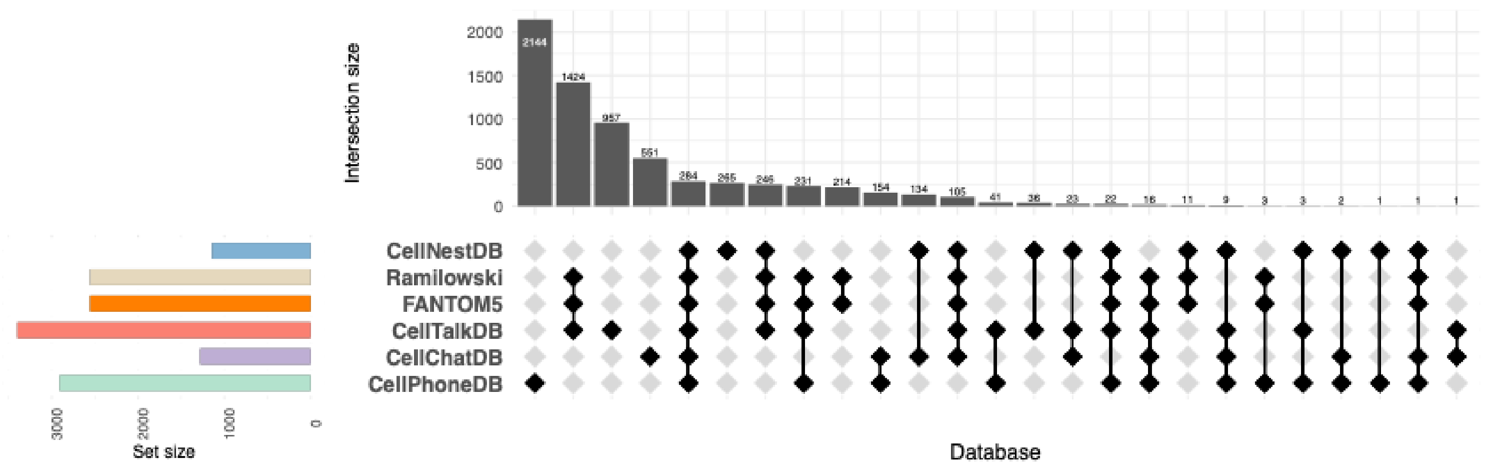

(B)

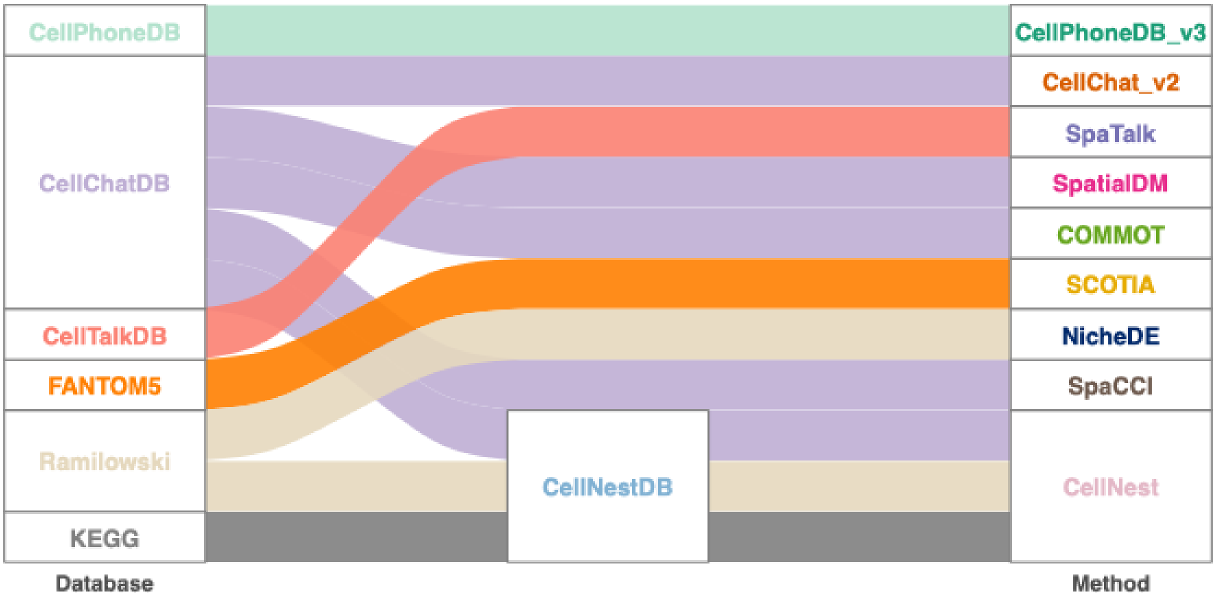

Fig. S32 | Summarization of the existing popular CCI databases and the construction of common-DB. (A) Presented the upset plot showing the overlap of ligand–receptor pairs among commonly used interaction databases. Bar heights indicate the size of each intersection. (B) Demonstrates the Sankey diagram illustrating the mapping from source ligand–receptor databases to the databases used by each CCI inference method. This visualization highlights heterogeneity in database composition across methods and motivates the use of harmonized (intersect) databases for fair benchmarking.

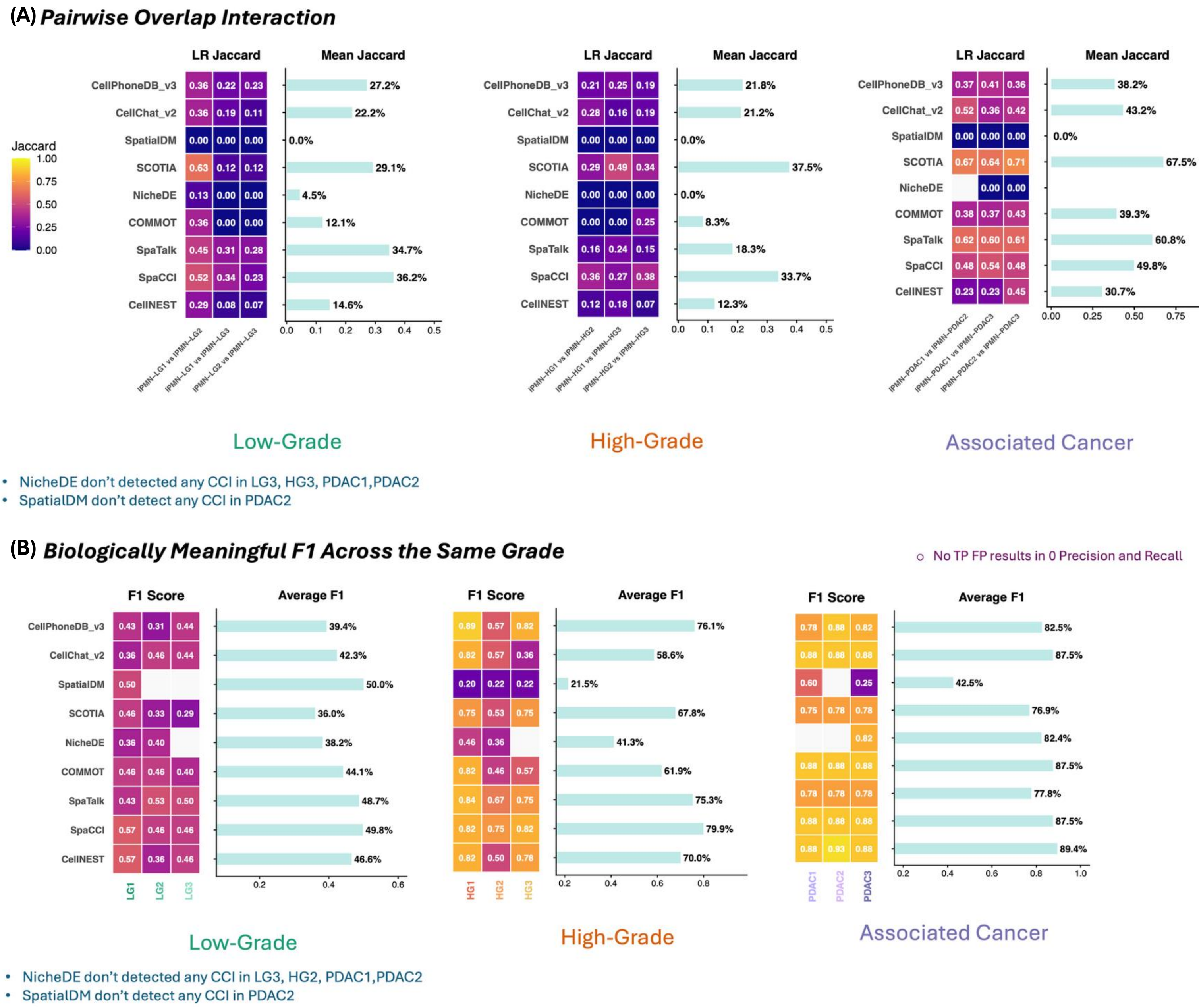

Fig. S33 | Evaluation of consistency and biological relevance using methods' native databases in 10x Visium IPMN dataset. *<u>(A) Pairwise overlap of inferred ligand–receptor (LR) interactions within the same pathological grade:</u>* Heatmaps show pairwise Jaccard similarity of LR interactions inferred by each method across samples within low-grade (LG), high-grade (HG), and associated cancer (PDAC) IPMN groups. Each column corresponds to one pairwise comparison within the same grade, and values indicate the fraction of overlapping interactions between sample pairs. Bar plots summarize the mean Jaccard similarity across all pairwise comparisons for each method. Higher values indicate greater consistency of inferred interactions across samples. Methods that did not detect any cell–cell interactions in specific samples yield zero overlap. Notably, NicheDE did not detect any interactions in LG3, HG3, PDAC1, or PDAC2, and SpatialDM did not detect interactions in PDAC2. *<u>(B) Biological relevance of inferred interactions assessed by F1 score within the same pathological grade:</u>* Heatmaps report F1 scores for biologically meaningful interactions identified by each method across samples within the same grade, evaluated using each method's native ligand–receptor database. Bar plots show the average F1 score across samples for each grade. Methods producing no true positive or false positive interactions result in zero precision and recall and therefore undefined or zero F1 scores.

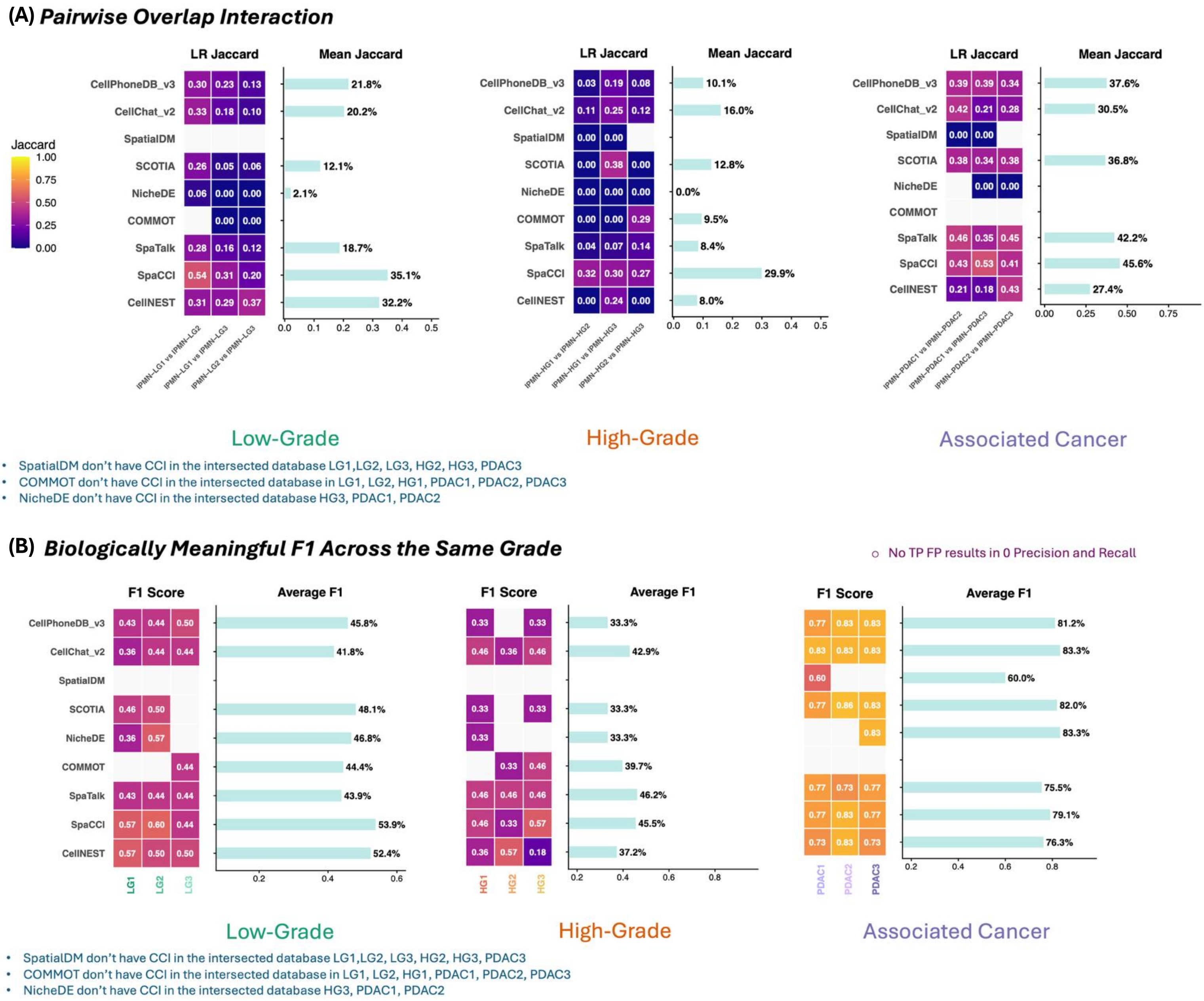

Fig. S34 | Evaluation of consistency and biological relevance using common-DB databases in 10x Visium IPMN dataset. *(A) Pairwise overlap of inferred ligand–receptor (LR) interactions within the same pathological grade using a common database:* Heatmaps display pairwise Jaccard similarity of LR interactions inferred by each method across samples within low-grade (LG), high-grade (HG), and associated cancer (PDAC) IPMN groups, after restricting all methods to a shared, intersected ligand–receptor database. Each column corresponds to a pairwise comparison within the same grade, and values indicate the proportion of overlapping interactions. Bar plots summarize the mean Jaccard similarity across all within-grade comparisons for each method. Methods that did not detect any interactions under the common-database setting yield zero overlap. SpatialDM, COMMOT, and NicheDE show reduced or absent overlap in multiple samples due to the absence of detectable interactions after database intersection. *(B) Biological relevance of inferred interactions assessed by F1 score within the same pathological grade using a common database:* Heatmaps show F1 scores for biologically meaningful interactions inferred by each method across samples within the same grade, evaluated using the common ligand–receptor database. Bar plots report the average F1 score across samples for each grade. Methods producing no true positive or false positive interactions result in zero precision and recall and therefore zero F1 scores.

(A)

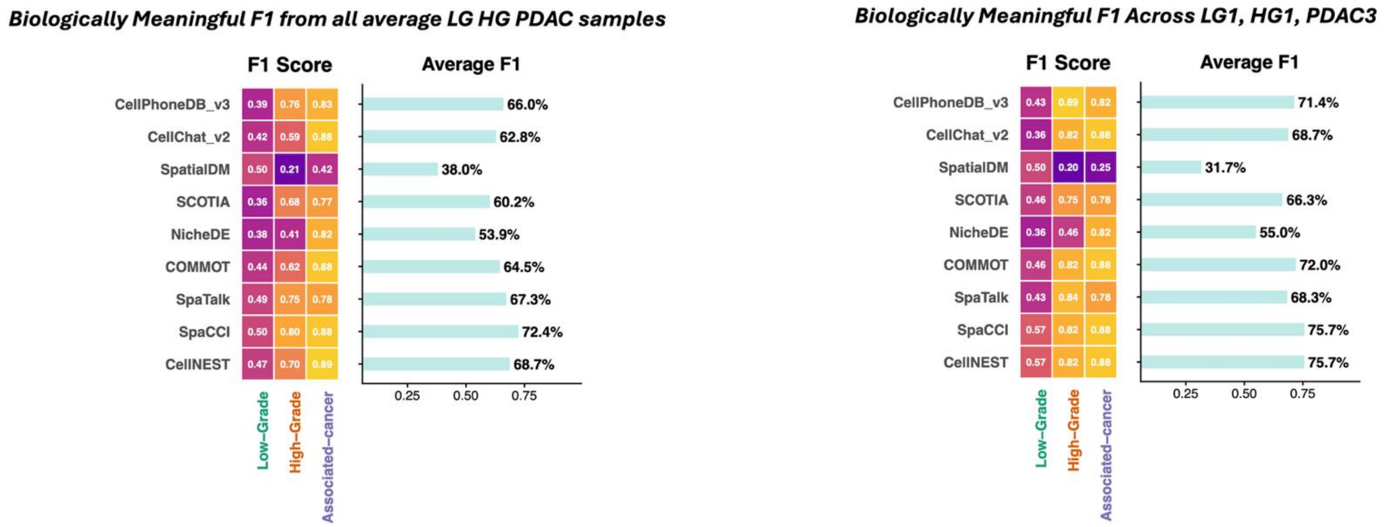

(B)

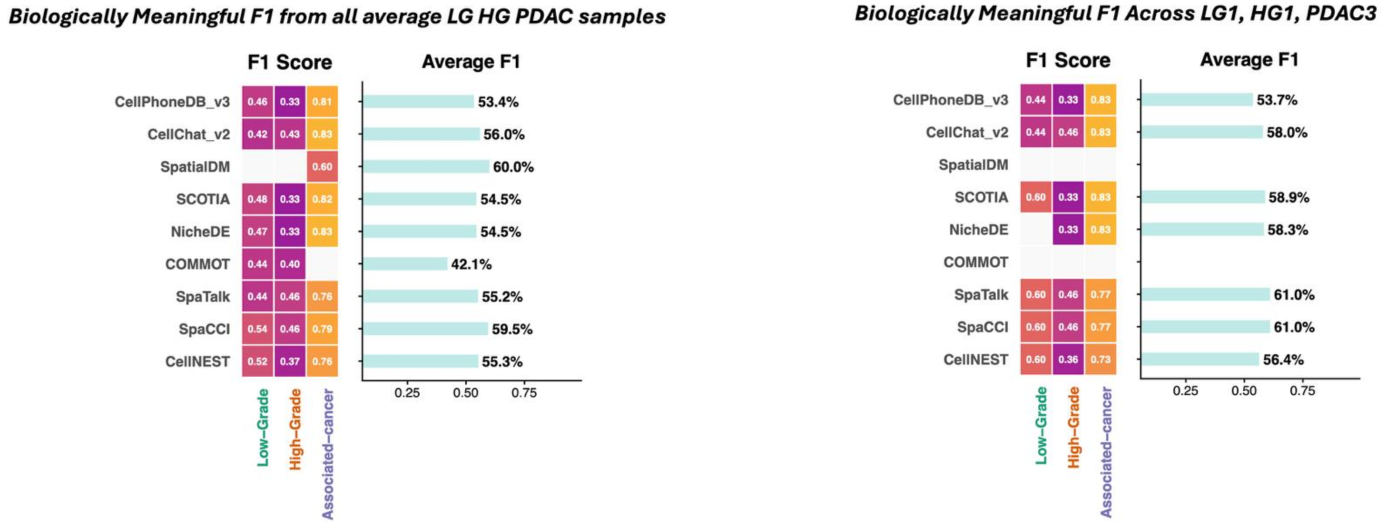

Fig. S35 | Comparison of biological relevance across grades using native and common ligand–receptor databases in the 10x Visium IPMN dataset. _(A) Biological relevance evaluated using each method's native ligand–receptor database:_ Heatmaps show F1 scores for biologically meaningful interactions inferred by each method across low-grade (LG), high-grade (HG), and associated cancer (PDAC) IPMN samples with bar plots summarizing the average F1 score. The left panel reports results averaged across all available LG, HG, and PDAC samples for each method. The right panel restricts evaluation to LG1, HG1, and PDAC3, which represent the subset of samples for which all methods produced valid cell–cell interaction inferences in the preceding analyses. This restriction enables a fair, sample-matched comparison of biological relevance across methods without confounding due to missing inferences. _(B) Biological relevance evaluated using a common ligand–receptor database:_ The same analyses as in (A) are shown after restricting all methods to a shared ligand–receptor database. Heatmaps display F1 scores across grades, and bar plots summarize average F1 values. Compared with native-database evaluations, the common-database setting reduces overall sensitivity for several methods; however, relative performance trends are preserved, with spatially aware methods maintaining higher and more stable F1 scores across grades.

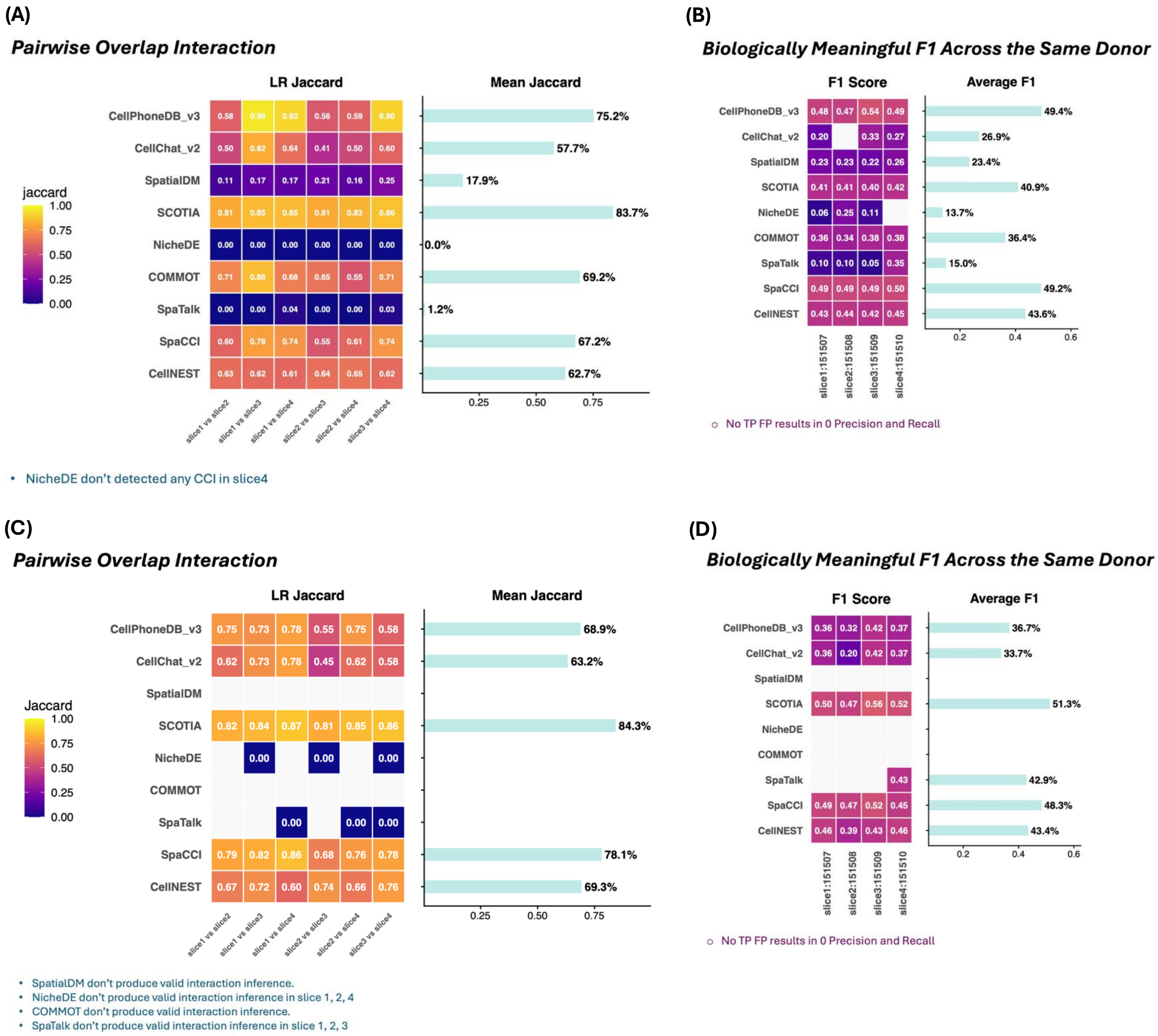

Fig. S36 | Evaluation of consistency and biological relevance using methods' native and common ligand–receptor databases in 10x Visium DLPFC dataset. *(A) Pairwise overlap of inferred ligand–receptor (LR) interactions across slices from the same donor using native-DB:* Heatmaps display pairwise Jaccard similarity of LR interactions inferred by each method across DLPFC slices derived from the same donor, evaluated using each method's native ligand–receptor database. Each column represents one slice–slice comparison, and values indicate the proportion of overlapping interactions. Bar plots summarize the mean Jaccard similarity across all pairwise comparisons for each method. *(B) Biological relevance of inferred interactions across slices from the same donor using native-DB:* Heatmaps show F1 scores for biologically meaningful interactions inferred by each method across individual slices from the same donor, with bar plots summarizing the average F1 score. *(C) Pairwise overlap of inferred LR interactions across slices using a common-DB:* The same analysis as in (A) is shown after restricting all methods to a shared, intersected ligand–receptor database. *(D) Biological relevance of inferred interactions across slices using common-DB:* Heatmaps report F1 scores across slices under the common-database setting, with bar plots showing average F1 values.
(Grey cells indicate slice pairs for which a method did not produce valid interaction inference in at least one slice, resulting in undefined overlap values. Methods that produced no true positive or false positive interactions in a given slice yield zero precision and recall, resulting in zero or undefined F1 scores)

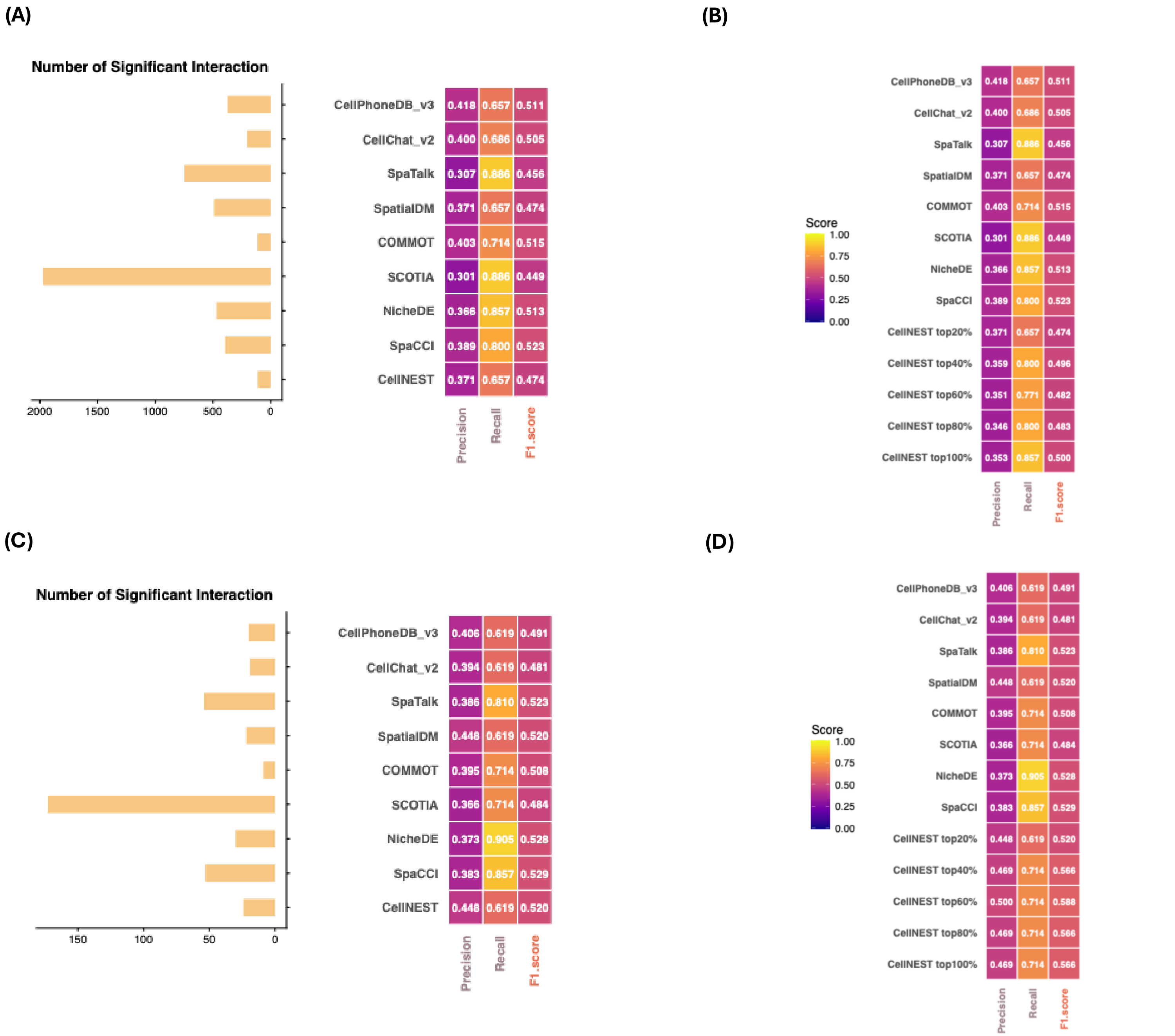

Fig. S37 | Evaluation of biological relevance using methods' native and common ligand–receptor databases in Stereo-Seq dataset (MOSTA E10.5). (A) Biological relevance evaluated using methods' native L-R databases: Bar plots show the number of significant cell–cell interactions inferred by each method, and heatmaps report precision, recall, and F1 score for biologically meaningful interactions. All methods except CellNEST apply statistical significance testing and include interactions based on method-specific p-value thresholds. Because CellNEST is a deep learning–based method that does not output p-values, top 20% representative cutoff is shown in this panel. (B) Sensitivity of biological relevance to CellNEST cutoff selection under native-DB. The same evaluation as in (A) is shown while varying the inclusion threshold for CellNEST by selecting the top 20%, 40%, 60%, 80%, or 100% of ranked interactions. Other methods are unchanged. (C) Biological relevance evaluated using a common (intersected) L-R database. The same analyses as in (A) are repeated after restricting all methods to a shared L-Rdatabase. Bar plots show the number of significant interactions and heatmaps report precision, recall, and F1 score. Restricting to a common-DB reduces the total number of detectable interactions for several methods. (D) Sensitivity of biological relevance to CellNEST cutoff selection under the common database. As in (B), CellNEST results are evaluated across multiple ranked-interaction cutoffs (top 20%–100%) under the common-DB setting.

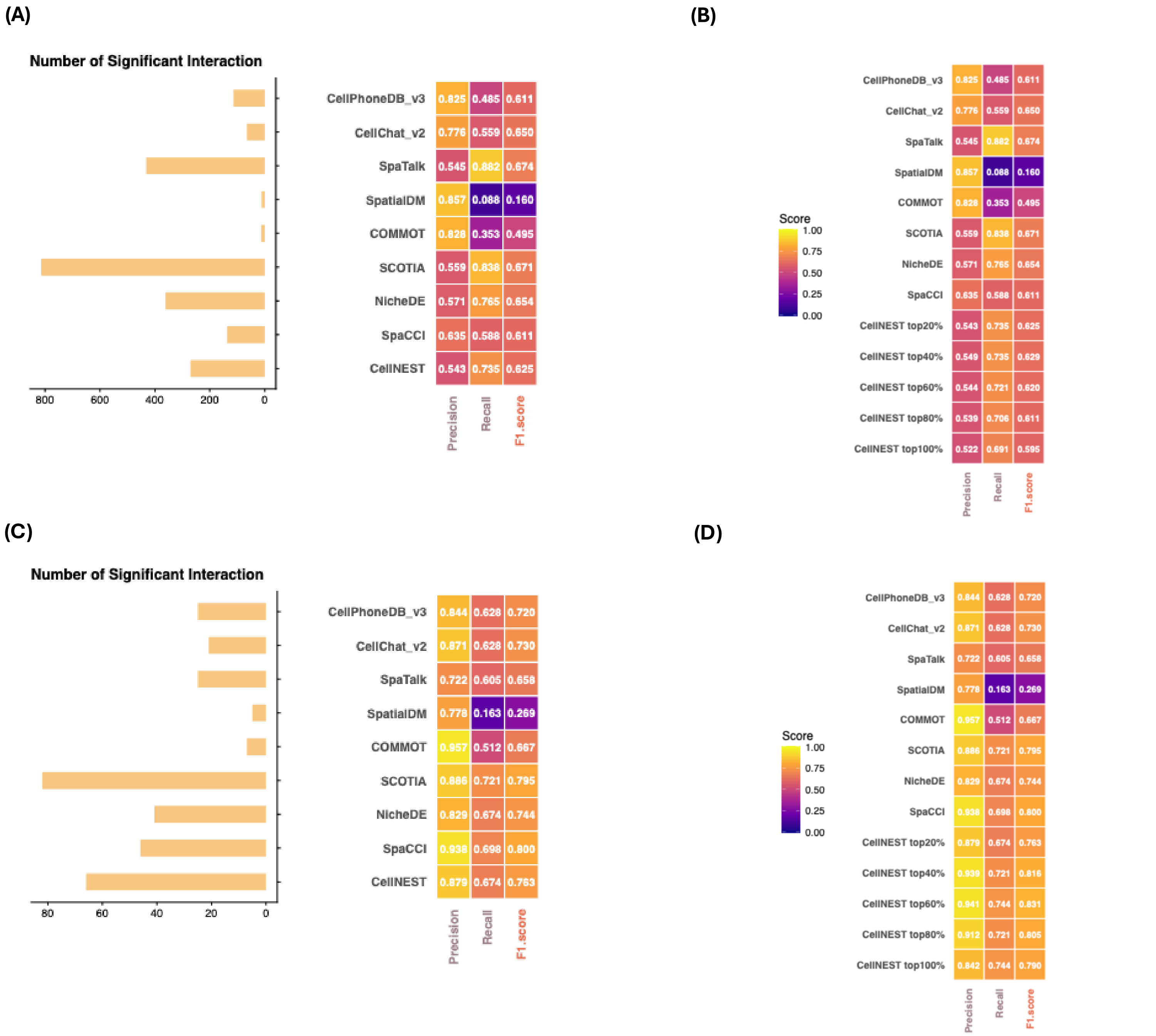

Fig. S38 | Evaluation of biological relevance using methods' native and common ligand–receptor databases in Xenium 5K FFPE lung cancer dataset. (A) Biological relevance evaluated using methods' native L-R databases. Bar plots show the number of significant cell–cell interactions inferred by each method, and heatmaps report precision, recall, and F1 score for biologically meaningful interactions in the Xenium 5K FFPE lung cancer dataset. All methods except CellNEST identify significant interactions based on method-specific statistical testing and p-value thresholds, where CellNEST use the top 20% representative rank cutoff is shown in this panel. (B) Sensitivity of biological relevance to CellNEST cutoff selection under native-DB. The same evaluation as in (A) is shown while varying the CellNEST inclusion threshold by selecting the top 20%, 40%, 60%, 80%, or 100% of ranked interactions. Results from other methods remain unchanged. (C) Biological relevance evaluated using a common (intersected) L-R database. The same analyses as in (A) are repeated after restricting all methods to a shared L-R database. Bar plots summarize the number of retained interactions, and heatmaps report precision, recall, and F1 score. Restricting to a common-DB reduces the total number of detectable interactions for several methods. (D) Sensitivity of biological relevance to CellNEST cutoff selection under the common-DB. As in (B), CellNEST performance is evaluated across multiple ranked-interaction cutoffs under the common-DB setting.

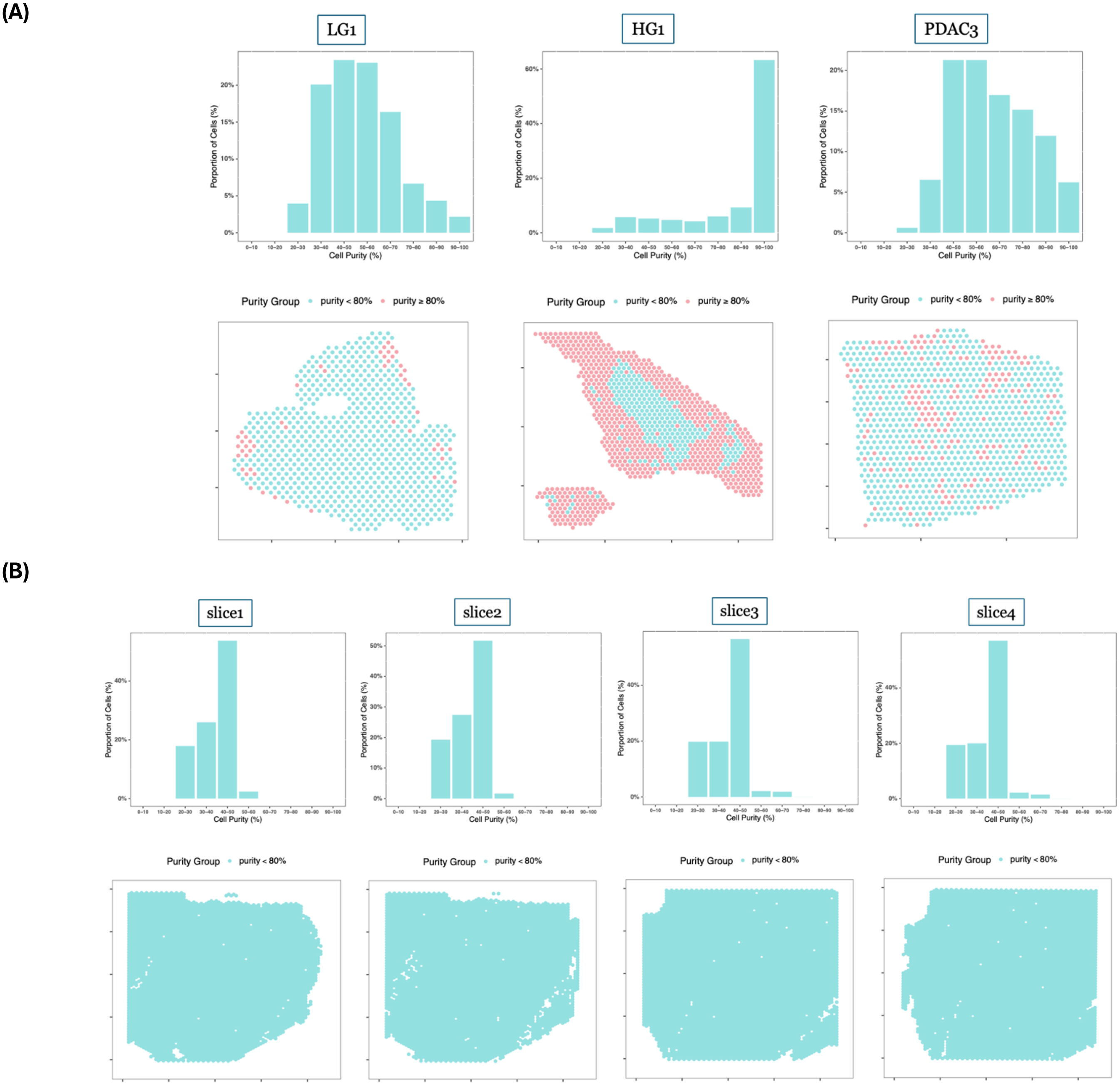

Fig. S39 | Assessment of spot purity in 10x Visium IPMN and DLPFC datasets.
(A) Distribution and spatial organization of spot purity in the 10x Visium IPMN dataset.
Top panels show histograms of spot purity for representative IPMN samples from low-grade (LG1), high-grade (HG1), and associated cancer (PDAC3) groups. Spot purity is defined as the proportion of the dominant cell type within each Visium spot. Bottom panels display the spatial distribution of spots, colored by purity group (purity < 80% versus purity ≥ 80%), illustrating the spatial heterogeneity of mixed-cell and high-purity spots across samples.

(B) Distribution and spatial organization of spot purity in the 10x Visium DLPFC dataset.
Histograms summarize spot purity distributions across four cortical slices (slice1–slice4), and corresponding spatial plots show the locations of spots colored by purity group. In contrast to IPMN samples, DLPFC slices exhibit predominantly low-purity spots, reflecting extensive cell-type mixing in brain tissue at Visium resolution.

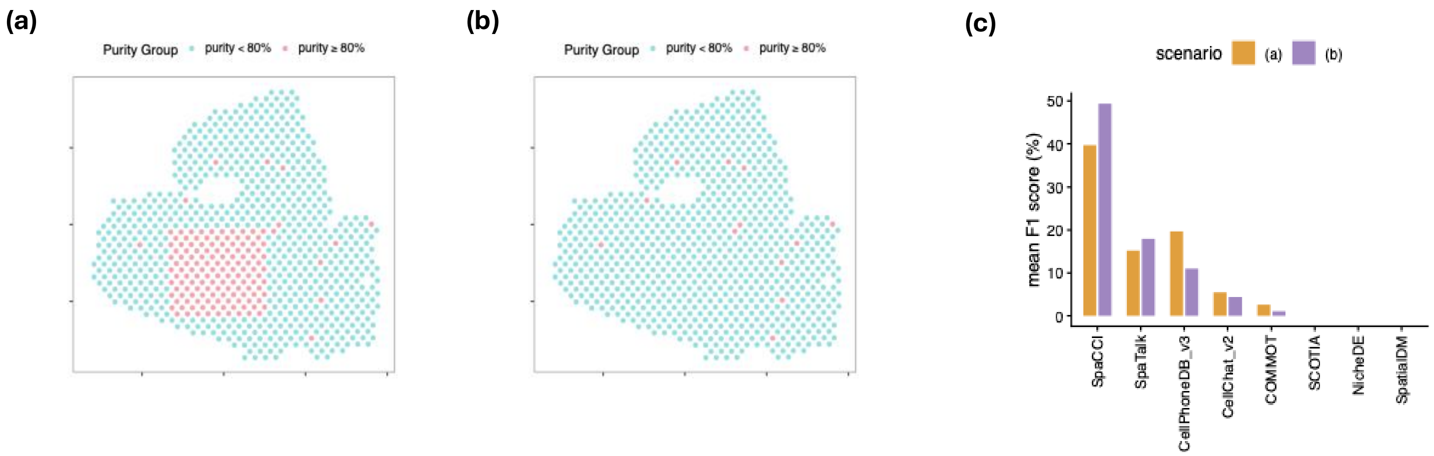

Fig. S40 | Spatial distribution and inference impact of spot purity in controlled spot-level simulations. Each point represents a spatial spot, colored by whether the dominant cell type accounts for ≥80% of the spot (high-purity, pink) or <80% (mixed, blue). (a) high-purity simulation, in which the central interaction region is enriched for high-purity spots. (b) mixed-purity simulation, in which the same region has approximately 50% purity. (c) mean F1 scores for each method under controlled spot-level simulations with a high-purity interaction region (a) and a mixed-purity interaction region (b).
